# Broad-spectrum resistance to bacterial blight in rice using genome editing

**DOI:** 10.1038/s41587-019-0267-z

**Published:** 2019-10-28

**Authors:** Ricardo Oliva, Chonghui Ji, Genelou Atienza-Grande, José C. Huguet-Tapia, Alvaro Perez-Quintero, Ting Li, Joon-Seob Eom, Chenhao Li, Hanna Nguyen, Bo Liu, Florence Auguy, Coline Sciallano, Van T. Luu, Gerbert S. Dossa, Sébastien Cunnac, Sarah M. Schmidt, Inez H. Slamet-Loedin, Casiana Vera Cruz, Boris Szurek, Wolf B. Frommer, Frank F. White, Bing Yang

**Affiliations:** 1 International Rice Research Institute, Metro Manila, Philippines; 2 Division of Plant Sciences, Bond Life Sciences Center, University of Missouri, Columbia, MO USA; 3 Department of Plant Pathology, University of Florida, Gainesville, FL USA; 4 IRD, CIRAD, Université Montpellier, IPME, Montpellier, France; 5 Department of Genetics, Development and Cell Biology, Iowa State University, Ames, IA USA; 6 Institute for Molecular Physiology and Cluster of Excellence on Plant Sciences (CEPLAS), Heinrich Heine Universität Düsseldorf and Max Planck Institute for Plant Breeding Research, Köln, Germany; 7 Erfurt University of Applied Sciences, Erfurt, Germany; 8 Institute of Transformative Bio-Molecules (WPI-ITbM), Nagoya University, Aichi, Japan; 9 Donald Danforth Plant Science Center, St. Louis, MO USA; 10 Present Address: College of Agriculture and Food Science, University of the Philippines Los Baños, Los Baños, Philippines; 11 Present Address: Department of Bioagricultural Sciences and Pest Management, Colorado State University, Fort Collins, CO USA

**Keywords:** Biotechnology, Genetics, Molecular biology, Physiology, Plant sciences

## Abstract

Bacterial blight of rice is an important disease in Asia and Africa. The pathogen, *Xanthomonas oryzae* pv. *oryzae* (*Xoo*), secretes one or more of six known transcription-activator-like effectors (TALes) that bind specific promoter sequences and induce, at minimum, one of the three host sucrose transporter genes *SWEET11*, *SWEET13* and *SWEET14*, the expression of which is required for disease susceptibility. We used CRISPR–Cas9-mediated genome editing to introduce mutations in all three *SWEET* gene promoters. Editing was further informed by sequence analyses of TALe genes in 63 *Xoo* strains, which revealed multiple TALe variants for *SWEET13* alleles. Mutations were also created in *SWEET14*, which is also targeted by two TALes from an African *Xoo* lineage. A total of five promoter mutations were simultaneously introduced into the rice line Kitaake and the elite mega varieties IR64 and Ciherang-Sub1. Paddy trials showed that genome-edited *SWEET* promoters endow rice lines with robust, broad-spectrum resistance.

## Abstract (web summary)

Genome editing of three *SWEET* gene promoters endows rice with resistance to all *Xanthomonas* bacterial blight strains tested.

## Main

*Xanthomonas oryzae* pv. *oryzae* (*Xoo*) is the etiological agent of bacterial blight disease in rice. The disease is most severe in southeast Asia but is increasingly damaging in west African countries, and results in substantial yield loss^1^. TALes from *Xoo* are injected by a type III secretion system into plant cells and recognize effector-binding elements (EBEs) in cognate *SWEET* host gene promoters, which results in induction of *SWEET* genes and production of sugars that enable disease susceptibility in rice^2,3^. An array of central repeats, which are 34–35 amino acids long, are present in each TALe and interact with EBEs via two repeat variable di-residues (RVDs) at the 12th and 13th position of each repeat^4,5^. Aberrant repeats, longer than 35 amino acids, are hypothesized to allow looping out of the repeat to accommodate alternate sequence binding for a particular TALe^6^.

Bacterial blight depends on TALe-mediated induction of at least one member of a family of sugar-transporter genes. Although rice has more than 20 *SWEET* genes, only those of clade III are reported to be induced by *Xoo*^7–10^. Although all five of the known clade III *SWEET* genes in rice can function as susceptibility genes for bacterial blight, only three are known to be targeted in nature^10^. More specifically, *SWEET11* expression is induced by strains encoding the TALe PthXo1, *SWEET13* by PthXo2 and *SWEET14* by any one of several TALes, namely AvrXa7, PthXo3, TalC and TalF (originally Tal5) ^7,9–15^ (Table 1). Effectors of *Xoo* that target clade III *SWEET* genes are referred to as major TALes owing to their strong virulence effect.

**Table 1:** Known EBEs in *SWEET* promoters

Host gene	TALe	EBE sequence
*SWEET11*	PthXo1	GCATCTCCCCCTACTGTACACCAC
*SWEET13*	PthXo2(A)	ATAAAAGCACCACAACTCCCTT
	PthXo2(A)	ATATAAGCACCACAACTCCCTT
	PthXo2B	ATAAAGCACCACAACTCCCTTC
	PthXo2C	ATAAAGCACCACAACTCCCTTC
*SWEET14*	TalC	CATGCATGTCAGCAGCTGGTCAT
	PthXo3	ATATAAACCCCCTCCAACCAGGTGCTAAG
	AvrXa7	ATAAACCCCCTCCAACCAGGTGCTAA
	TalF	AAGCTCATCAAGCCTTCA

Naturally occurring resistance has arisen as the result of nucleotide polymorphisms in EBEs of *SWEET* promoters. EBE alleles of *SWEET11* that are not recognized by PthXo1 are collectively referred to as the recessive resistance gene *xa13*. Rice varieties containing *xa13* are resistant to strains that solely depend on PthXo1 for *SWEET* induction. Most *indica* rice varieties carry a *SWEET13* allele that contains four adenines in the EBE for PthXo2, and rice lines carrying this allele are susceptible to PthXo2-dependent strains^12^. A rare exception is the recessive resistance allele *xa25*, which contains three adenines in the EBE for *SWEET13* in the *indica* cultivar Minghui 63, conferring resistance to strains that depend solely on PthXo2^16^. A similar recessive resistance allele in *japonica* rice varieties is equally effective against strains relying on PthXo2 (ref. ^12^). Additional naturally occurring recessive EBE polymorphisms that confer resistance to strains carrying PthXo2, and the overlapping EBEs for PthXo3, TalF and AvrXa7 have subsequently been identified in the promoters of *SWEET13* and *SWEET14*, respectively, in germplasm collections^17,18^.

Rice susceptibility genes are good targets for genome editing for disease resistance. TALe-mediated susceptibility is particularly modifiable. For instance, transcription-activator-like effector nuclease (TALEN)-directed mutations in *SWEET14* created lines resistant to strains carrying PthXo3/AvrXa7 (refs. ^19,20^). Importantly, characterization of TALes and their cognate EBEs indicates that a limited set of EBEs are targeted by extant *Xoo* populations. At the same time, relatively few strains have been assessed for TALe gene content, and optimized genome editing requires knowledge of the TALe variants present in bacterial populations. Targeting both prevalent and rare TALes should increase the durability of resistance. In addition, the development of better tools for TALe characterization, which is a challenging and laborious procedure owing to their repetitive DNA sequences and presence of multiple copies per genome, would facilitate identification of novel TALes and determination of gene-editing strategies^21–23^.

Here we report a detailed characterization of 63 *Xoo* strains from diverse geographic areas which includes genome sequence, the TALes present and virulence assays. Building on the understanding of TALes and their *SWEET* gene target EBEs, we developed a strategy to use CRISPR–Cas9 genome editing to engineer broad and durable resistance to bacterial blight in rice. An accompanying manuscript^24^ reports the development of a disease management diagnostic tool kit for customized deployment of the resistant lines we report here.

## Diversity of TALes in 63 *Xoo* strains

**Fig. 1:**
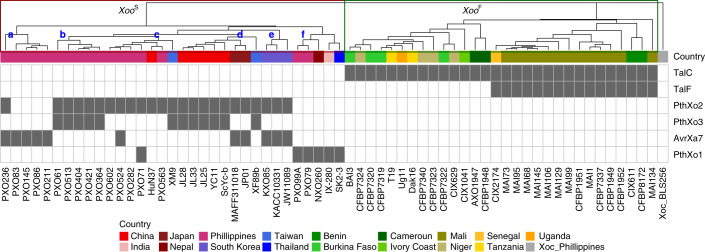
Distribution of *SWEET*-inducing TALes in *Xoo* strains. Heat map indicates presence (gray) or absence of TALes shown or predicted to bind to *SWEET* promoters in fully sequenced *Xoo* genomes. Origin is indicated. TALes were grouped by DisTAL (Supplementary Fig. 1). Six groups containing previously characterized *SWEET*-inducing TALes are shown. A parsimony tree (top) that is based on whole-genome SNPs obtained using kSNP is shown, highlighting the two main *Xoo* lineages: *Xoo*^S^ (Asia) and *Xoo*^F^ (Africa). Six sublineages were defined for *Xoo*^S^, and are indicated in bold blue letters (a–f). Seventy-three percent of branches had support values over 0.9 as determined by FastreeMP implemented in kSNP3.

An assessment of the diversity of the major TALes in extant strains of *Xoo* was obtained by analyzing *Xoo* genome sequence data and virulence assays on rice lines with *SWEET* promoter polymorphisms. TALe gene content was derived from an analysis of complete genome sequences of 33 strains from Asia (*Xoo*^S^) and 30 strains from Africa (*Xoo*^F^), which were either in databases or newly sequenced (Supplementary Table 1). Whole-genome, single nucleotide polymorphism (SNP)-based parsimony trees clearly separated *Xoo*^S^ from *Xoo*^F^ genomes, revealing two distinct evolutionary lineages (Fig. 1). Trees based on alignments of repeat regions of 856 TALes revealed two major clusters (Supplementary Fig. 1). Expansion and diversification of TALe loci were more frequent in *Xoo*^S^ (18–21 TALes per genome) than in *Xoo*^F^ genomes (nine TALes per genome). Expansion and diversification of TALe loci is also reflected by the number of *SWEET-*gene-inducing TALes, of which there are 13 variants (unique RVD sequences) in *Xoo*^S^ strains and three variants in *Xoo*^F^ strains (Supplementary Fig. 2).

TALes predicted to induce *SWEET* genes (major TALes) were present in all *Xoo* strains, regardless of origin (Fig. 1). Indeed, all strains possessed one or more close homologs of the known major TALe groups (PthXo1, PthXo2 and PthXo3/AvrXa7). PthXo3/AvrXa7 is treated as a single class owing to the closely overlapping EBEs in the *SWEET14* promoter. Asian strains had approximately equal numbers of PthXo2 (targeting *SWEET13*) and PthXo3/AvrXa7 *(SWEET14*) (24 versus 23, respectively; Fig. 1). Most Asian strains had multiple major TALes with a combination of PthXo2 with either PthXo3 or AvrXa7 (Fig. 1). None of the strains contained both PthXo3 and AvrXa7 (Fig. 1). In African lineages, the two known major TALes of *Xoo* are TalC and TalF, which each induce *SWEET14* using non-overlapping EBEs^10,13^. Half of the analyzed African strains had both TalC and TalF, while none had TalF alone (Fig. 1). Overall, we found six non-overlapping EBEs in three *SWEET* promoters, if we treat the EBEs of PthXo2 variants that occur in some rice varieties as a single EBE (Table 1). These results support the hypothesis that activation of *SWEET* genes was a critical evolutionary step that occurred independently in different geographic regions during *Xoo* adaptation to rice.

## Diversity of TALes from infection trials

**Fig. 2:**
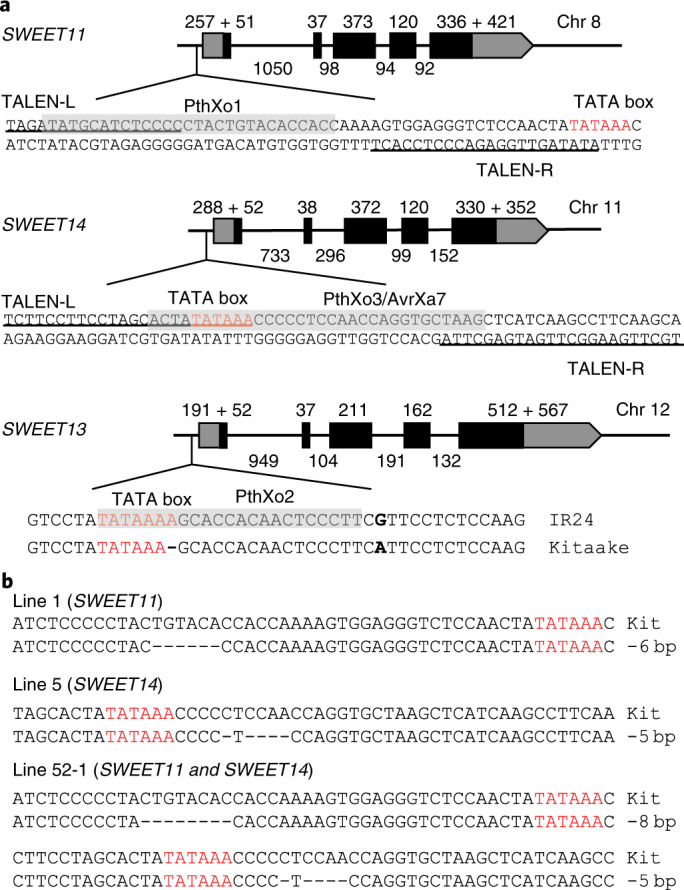
TALEN-induced variants and natural *SWEET* variants to assess prevalence of major TALes in 105 *Xoo* strains. **a**, Schematic gene structures of *SWEET11* and *SWEET14* with promoter sequences targeted by respective TALes shaded and with TALEN-bound sites underlined. Two natural variants of *SWEET13* in two rice varieties are shown. The EBE for the respective TALe is highlighted in gray. **b**, Genotypes of three Kitaake-derived lines for disease assays. All lines have the Kitaake allele for *SWEET13*.

To estimate the prevalence and variation of TALes in geographically diverse *Xoo* strains, a collection of 105 *Xoo* strains was screened against the *japonica* rice variety Kitaake and derivative Kitaake lines carrying mutations in the three *SWEET* genes normally targeted by TALes. Kitaake contains a natural variant allele of *SWEET13* that is not recognized by PthXo2^12^. Strains were also screened against lines with alternate alleles of *SWEET11* and *SWEET14* generated by TAL effector nucleases (TALEN) both singly and in combination (Fig. 2 and Supplementary Table 2).

Ten of 105 *Xoo* strains were not virulent on the *japonica* reference line Kitaake. These ten strains were virulent on an *indica* reference line IR24 (Supplementary Fig. 3). IR24 contains few known resistance genes and serves as a recurrent susceptible parent line for breeding diverse near-isogenic resistance gene lines against bacterial blight^25^. These strains may be solely dependent on PthXo2 for induction of the IR24 *indica* allele of *SWEET13*, as the EBEs in the other *SWEET* gene promoters are the same in Kitaake and IR24. Alternatively, Kitaake may have a resistance gene that is effective against the ten strains. These ten strains were excluded from further screening.

Of the remaining 95 Xoo strains, 18 were not virulent on edited rice line 1, which is defective in the EBE targeted by PthXo1 in *SWEET11*, and 69 strains were not virulent on line 5, which is defective in the EBEs of AvrXa7 and PthXo3 within *SWEET14* (Fig. 2 and Supplementary Table 2). Eighty-seven strains (18 + 69) were also not virulent on line 52-1, which harbors mutant alleles of both *SWEET11* and *SWEET14*, while the remaining eight strains (95 – 87) were virulent on line 52-1 (Fig. 2 and Supplementary Table 2). These findings indicate that 87 strains from the screen rely on major TALes that target known EBEs of *SWEET11* and *SWEET14*, whereas eight ‘deviant’ strains have different virulence abilities and, presumably, different TALes. These eight strains originated from the Philippines (PXO61, PXO364, PXO404, PXO421 and PXO513), the Korean peninsula (JW89011 and KXO85) and the African continent (AXO1947) (Supplementary Table 2). The virulence assay could not distinguish the deviant strains that, individually, carry both *avrXa7*/*pthXo3*-like and *pthXo2*-like or *pthXo1* and *pthXo2*-like TALe genes.

## Characterization of deviant *Xoo* strains

Genome sequencing and TALe content analysis of the seven deviant Asian strains identified *pthXo3* in the five Philippine strains (PXO61, PXO364, PXO404, PXO421 and PXO513) and *avrXa7* in the two Korean strains (JW89011 and KXO85) as judged by the similarity of RVDs (Fig. 1, Supplementary Fig. 2 and Supplementary Table 3). *pthXo1* was not found in any of the deviant strains. However, on the basis of similarity of the RVDs, all seven genomes had one of two variant *pthXo2*-like genes (Fig. 1 and Supplementary Table 3). One variant, named PthXo2B, is present in all five strains from the Philippines, while PthXo2C is present in the two Korean strains. All three PthXo2-related effectors have repetitive domains of 22 RVDs. PthXo2B differs in eight RVDs, while PthXo2C contains seven variant RVDs as compared to PthXo2 (Supplementary Fig. 4a). Both PthXo2B and PthXo2C were predicted to bind to the same EBE (TATAAAGCACCACAACTCCCTTC) within the *SWEET13* promoter of Kitaake and other *japonica* varieties. The predicted EBEs of PthXo2B and PthXo2C differ from the EBE for PthXo2 of *SWEET13* in IR24, which is representative of most *indica* alleles (Supplementary Fig. 4b). Realigning RVDs with their respective EBEs and accounting for the shift in sequence owing to the single ‘A’ variation, the differences in RVD order between PthXo2 and PthXo2B or PthXo2C are reduced to four and five variant RVDs, respectively (Supplementary Fig. 4b). In addition to differences in the RVDs, PthXo2B and PthXo2C have 36-amino-acid repeats at RVD 9 and 12 relative to PthXo2. Atypical repeats have been found to accommodate alternative EBE binding in the major TALe AvrXa7 (ref. ^6^). The differences between PthXo2B and PthXo2C and the presence of PthXo3 or AvrXa7 reflect the overall separation of the sublineages from the Philippines and Korea (Fig. 1; sublineages b and e, respectively).

**Fig. 3:**
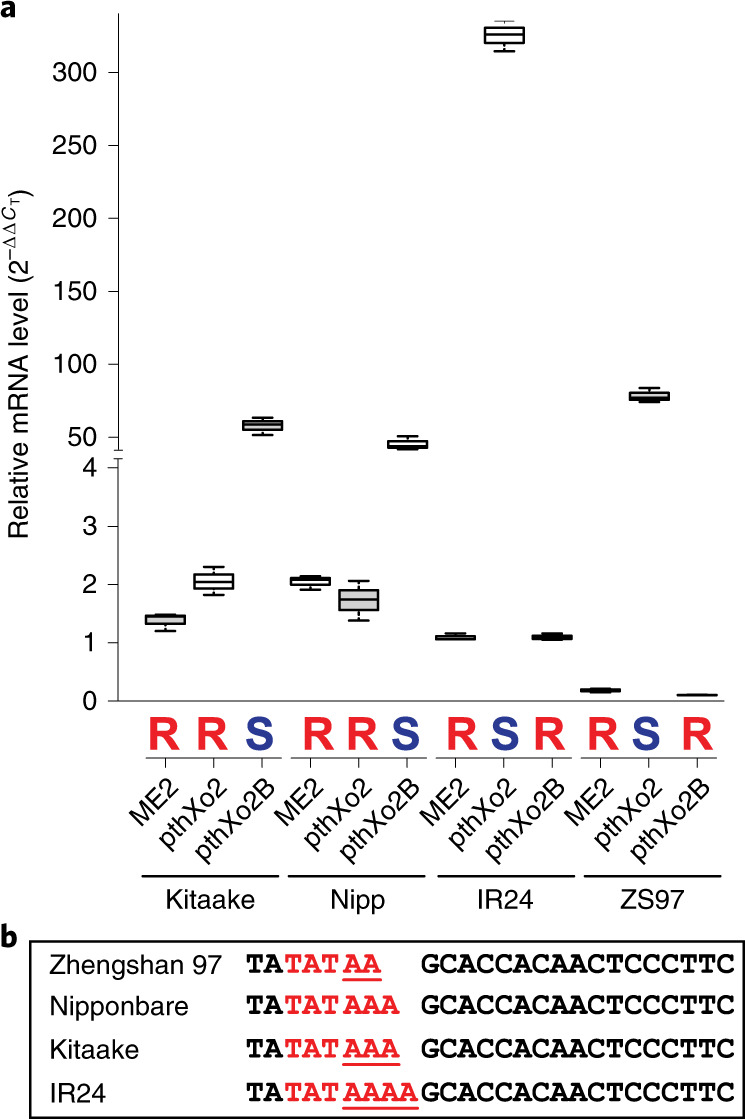
Functional analysis of PthXo2 homologs in four rice varieties. The genes *pthXo2* and *pthXo2B* were introduced into ME2 (a non-virulent derivative of PXO99^A^, lacking *pthXo1)*. **a**, *SWEET13* induction in four rice varieties by *Xoo* strains (rice actin 1 as control). Data are plotted using BoxPlotR (see Methods); center lines show medians, box limits indicate the 25th and 75th percentiles. The experiment was repeated three times independently with comparable results. Bold letters above strains indicate susceptibility score (red R, resistance; blue S, susceptibility). Actual data for resistance or susceptibility are shown in Supplementary Fig. 5. ZS, Zhengshan. **b**, Nucleotide variations (underlined) in EBE regions for PthXo2-related TALes in selected rice varieties. The predicted TATA box is highlighted in red.

*Xoo* strains with PthXo2 alone are incompatible on Kitaake-derived lines owing to the inability to induce the *japonica* allele of *SWEET13*^12^. The presence of *pthXo2B* or *pthXo2C* could explain the virulence phenotypes of the seven deviant strains on Kitaake and the double-edited Kitaake line 52-1. Both effectors are predicted to enable induction of the *japonica* allele of *SWEET13*. To test this possibility, the candidate gene for PthXo2B from PXO61 was cloned and introduced into strain PXO99^A^ME2 (hereafter ME2), which is not pathogenic on any of the tested rice lines owing to a null mutation in the sole major TALe gene *pthXo1* of PXO99^A^ (ref. ^9^). The *japonica* allele of *SWEET13* was induced in Kitaake and Nipponbare only upon inoculation with ME2 carrying *pthXo2B*, while relative expression in the *indica* allele in variety IR24 was low upon inoculation with ME2 or ME2 carrying PthXo2B (Fig. 3a). The *indica* allele of *SWEET13* in IR24 was induced upon inoculation with ME2 harboring PthXo2 (Fig. 3a). Lines in which *SWEET13* was induced scored as susceptible based on lesion length, and lines without *SWEET13* expression scored as resistant (Supplementary Fig. 5). An additional rice line (Zhengshan 97) carrying a different polymorphism in the EBE region for PthXo2-related effectors, namely a two A nucleotide deletion as compared to the *indica* allele, was also tested (Fig. 3b). The *SWEET13* allele in Zhengshan 97 behaved similarly to the IR24 allele and scored as susceptible to ME2 with PthXo2 and resistant to ME2 alone or with PthXo2B (Fig. 3 and Supplementary Fig. 5).

## Stacking edited EBEs for broad-spectrum resistance to *Xoo*

We tested whether stacking multiple mutations in the EBEs of three *SWEET* promoters known to be targeted by major TALes would provide resistance to most, if not all, *Xoo* strains in a single rice line. Using a multiplex CRISPR editing approach (Supplementary Figs. 6–8), a series of mutant lines were generated from Kitaake (Supplementary Table 4). The EBE mutant lines were first challenged with ME2 transformants, each carrying a gene for either PthXo1, PthXo2B, AvrXa7, TalC or TalF. The lines with the respective promoter mutations were resistant to ME2 derivatives carrying the corresponding TALes (Supplementary Table 5). When challenged with eleven field strains, including the seven RVD-variant *Xoo* strains that can induce *SWEET13*, one edited Kitaake line (11.1.3) was resistant to all strains (Supplementary Table 5). The 11.1.3 line conferred moderate resistance to the *Xoo*^F^ lineage strain AXO1947, which, of the known TALes, only contains TalC, suggesting the presence of another unknown effector. A similar observation was obtained upon inoculation of *Xoo*^F^ strain MAI1, which has both TalF and TalC (Fig. 1). Parallel tests using *SWEET* knockout mutants showed that AXO1947 likely depends on both *SWEET13* and *SWEET14* (ref. ^24^).

## Genome-edited *Xoo*-resistant mega varieties

**Fig. 4:**
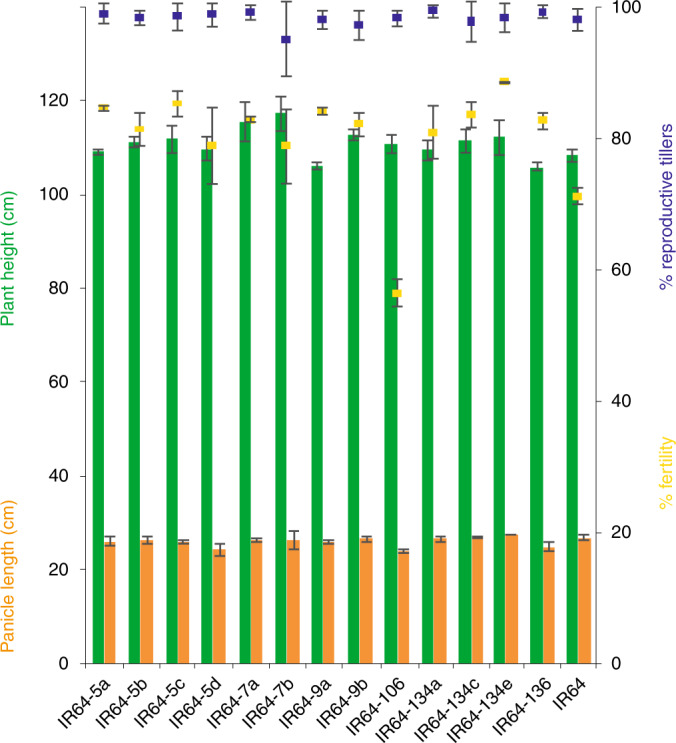
Agronomic traits in selected genome-edited mega variety lines as compared to the parental controls. Performance of edited IR64-IRS1132 lines in the T3 generation (left axis: plant height in green, panicle length in orange; right axis: percentage fertility in yellow and percentage of reproductive tillers in blue) relative to parental controls (IR64) (*n* = 15, no significant difference between IR64 and edited lines except fertility for line IR64-106, *P* *<* 0.01). Microfield experiments in screenhouses for agronomic trait assessments were conducted using randomized complete block design with three replicates. Trials were done in a single season. Data are presented in Supplementary Fig. 10.

**Fig. 5:**
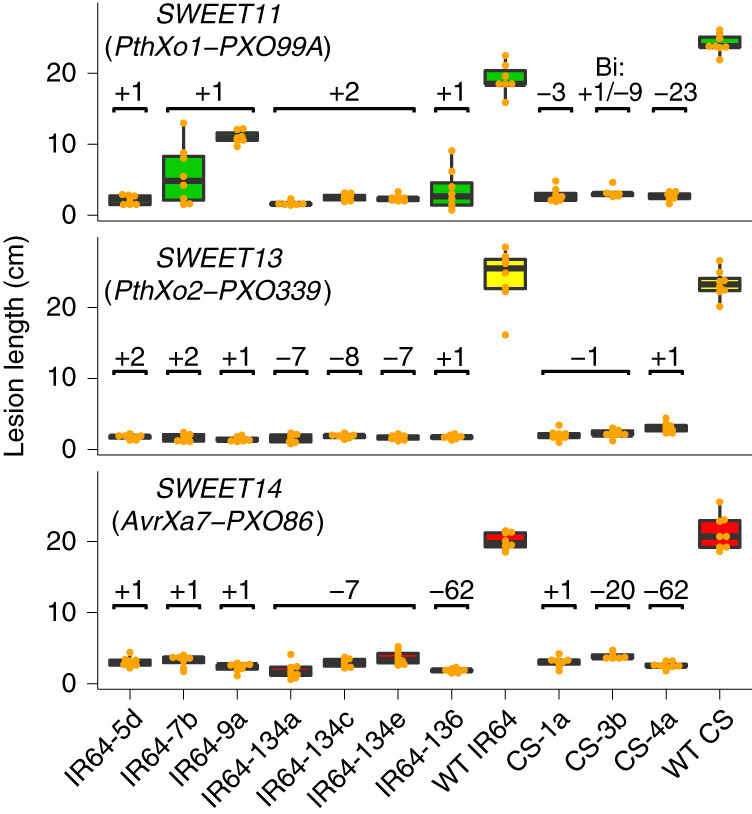
Resistance of *SWEET* promoter edited IR64 and Ciherang-Sub1 lines. Reactions of selected mutant lines of T3 IR64-IRS1132 and T2 Ciherang-Sub1-IRS1132 lines as compared to parental IR64 and Ciherang-Sub1 controls to infections with *pthXo1-*, *pthXo2-* and *avrXa7*-dependent *Xoo* strains (PXO99, PXO339 and PXO86, respectively). Different sequence alterations in the target *SWEET* promoters of edited lines resulted in varying levels of resistance to corresponding *Xoo* strains. Lesion lengths (cm) were measured 14 d after infection of specified plants. Types of mutations are indicated. Phenotyping experiments were conducted with four replicates per strain and two plants per replicate (*n* = 8), and between three and six inoculated leaf samples were scored per plant. All tested lines carried the same mutations in both alleles, except those marked as ‘Bi’ (biallelic, that is, two different mutations in EBE). Center lines show medians; box limits indicate 25th and 75th percentiles determined in the R software package; whiskers extend 1.5 times the interquartile range from 25th and 75th percentiles. Means for all edited lines are significantly different from controls (Dunnett’s test; *P* < 2 × 10^−^^16^). A two-sided test was used.

**Fig. 6:**
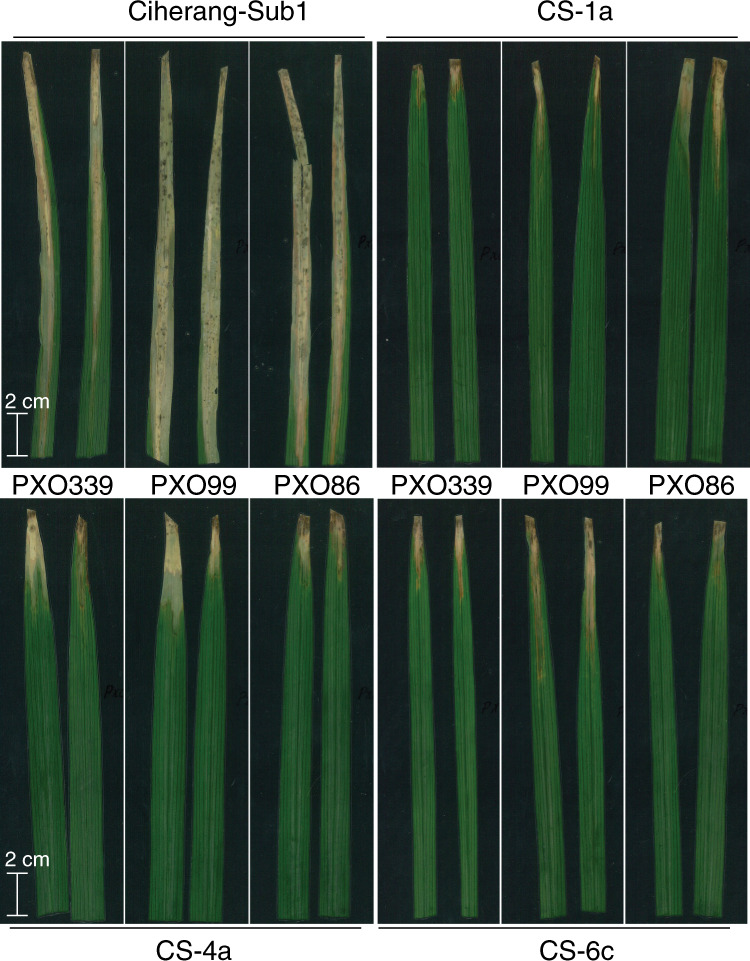
Resistance of three genome-edited Ciherang-Sub1 lines to three representative *Xoo* strains. Appearance of lesions resulting from clipping leaf tips using scissors that had been dipped into bacterial cultures of three different *Xoo* strains (PXO399, PXO99 and PXO86, as indicated), for three genome-edited Ciherang-Sub1 mutant lines CS-1a, CS-4a and CS-6c (T2 generation) as compared to the parental control (Ciherang-Sub1). The experiment was repeated twice independently.

Rice mega varieties, lines cultivated over one million hectares, are semidwarf and high-yielding lines that usually perform uniformly across multiple environments^26^. If the combined *SWEET* editing events tested here do not affect the normal physiological function of the genes, we predict that the same combinations would not impair agronomic traits in rice mega varieties, and would thus be useful for rice breeding programs. Genome editing by CRISPR–Cas9 was used to target *SWEET* promoters in two rice mega varieties, IR64 and Ciherang-Sub1 (Supplementary Fig. 9). Key agronomic traits were measured in confined paddy experiments. Before trials, the CRISPR-edited *SWEET* promoters of all mutant lines were sequenced, and lines were selected on the basis of the diversity of mutations in the respective EBEs (Supplementary Table 6). Overall, 13 IR64-IRS1132 and 18 Ciherang-Sub1-IRS1132 *Cas9*-free lines, spanning 30 combinations of EBE mutations in *SWEET11*, *SWEET13* and *SWEET14* were tested (Supplementary Table 7). Agronomic assessments and multivariant analysis of height, panicle length, number of reproductive tillers and fertility rate indicated that most lines performed similarly to wild-type parents (Fig. 4 and Supplementary Fig. 10). Only line IR64-106 showed phenotypic differences regarding yield, panicle length and fertility (reduced by 10% under paddy conditions). A range of lines with different insertions, deletions and substitutions in *SWEET11* (*n* = 6), *SWEET13* (*n* = 8) and *SWEET14* (*n* = 7) were selected, assuming that larger EBE differences will be less likely to match TALe variation in pathogen populations. Distinct combinations of these 21 *SWEET* variants were distributed across nine rice lines. The nine lines were challenged with tester *Xoo* strains harboring single major TALes. All nine lines were resistant to all *Xoo* strains tested^24^ (Figs. 5 and 6). Seven variants of *SWEET13*, ranging from two-nucleotide insertions (+2) to a seven-nucleotide deletion (−7), conferred resistance. Line IR64-9a carried only SNPs in *SWEET11*, which were insufficient to abrogate PthXo1 function (Fig. 5).

## Discussion

We set out to evaluate our hypothesis that editing known *SWEET* EBEs will endow rice with broad resistance to bacterial blight. The success of our approach depended on extant *Xoo* strains fitting the profile of the relatively few *Xoo* strains that have been genetically characterized. Indeed, analysis of a broad collection of 63 *Xoo* strains found that all strains targeted, or were predicted to target, *SWEET11*, *SWEET13* and *SWEET14*, as previously reported^8,10,27^. Furthermore, most *Xoo* strains, if not all, target some combination of six short EBE sequences present in the promoters of the three *SWEET* genes. We applied multiplexed CRISPR–Cas9 genome editing to systematically interfere with *SWEET* gene induction at all known major TALe EBEs and engineer Kitaake rice resistant to all currently known strains of *Xoo*. Kitaake is an excellent cultivar for proof-of-concept owing to high regeneration and rapid flowering cycle. The lines generated here will serve as diagnostic tools for rapid evaluation of the virulence of novel *Xoo* isolates^24^. Although Kitaake can be used for breeding resistance genes into Japanese and Chinese varieties, the *japonica* cultivar is not optimal as breeding material in large parts of southeast Asia and Africa, which use mainly *indica* varieties. Therefore, we also applied the same editing approach in two mega varieties, IR64^26^ and Ciherang-Sub1^28^. The edited mega variety lines grew normally, without yield suppression, and were resistant to three representative strains of bacterial blight carrying the known major TALes.

To devise an efficient genome editing strategy it was important to identify a panoply of TALes and cognate EBEs to guide engineering of broadly applicable bacterial blight resistance. Sequence analysis of the seven deviant *Xoo* strains from Asia and relatedness of TALes with close, but not identical, RVDs led us to propose that variants of PthXo2 target variant EBEs in *SWEET13*. These seven Asian strains retained virulence on the first-generation of genome-edited EBE lines in the Kitaake background, indicating that the variant strains either carry another major TALe targeting an undefined susceptibility gene or a variant major TALe targeting *SWEET11*, *SWEET13* or *SWEE14* at an alternate EBE. The two newly identified *pthXo2* members (*pthXo2B* and *pthXo2C*) were predicted to target the *SWEET13* promoters found in many *japonica* varieties, including Kitaake and Nipponbare. Indeed, PthXo2B conferred virulence to ME2, a strain lacking major TALe genes, on both Kitaake and Nipponbare, concomitantly with *SWEET13* induction. Thus, the presence of PthXo2B or PthXo2C in the deviant strains is sufficient to explain their virulence on the TALEN-edited lines. Ultimately, several lines with CRISPR-mediated edits at the PthXo2B-cognate EBE of Kitaake were resistant to *pthXo2B*-containing strains, indicating that the Kitaake allele for *SWEET13* was responsible for the susceptibility of line 52-1. Recent testing of all TALe genes of the strain PXO61 did not reveal any new major TALe other than PthXo3 and PthXo2B^29^. *Xoo*^S^ strains carrying PthXo2B were obtained in the Philippines, where several bacterial blight outbreaks occurred. This sublineage is characterized as local races 1 and 9d, which have not been reported in other countries. At the same time, the Philippine archipelago may offer a safe geographic reservoir for less prevalent strains. Both strains from the Korean peninsula possess PthXo2C. The sampling of Korean strains was limited, and we are unable to state whether genes for PthXo2C are the rule or the exception. What is clear is that different variants of PthXo2 family are present in the global *Xoo* population, suggesting an ongoing adaptation to nucleotide variations in the *SWEET13* promoter^12,16,17^.

African strains were shown to possess less TALe diversity and fall into two sublineages. All strains harbor the major virulence TALe, TalC, which targets *SWEET14*. Members of the second sublineage have TalC and TalF, the latter also targeting *SWEET14*. Another feature of the African strains is that loss of TalC function results in significantly reduced lesion length^13^. *Xoo* strains relying solely on TalC retained full virulence on Kitaake lines mutated in the TalC EBE within *SWEET14* (ref. ^19^).

We show here that mutation of the TalC EBE in the promoter of *SWEET14* in Kitaake led to only moderate resistance to the African strain AXO1947. By contrast, double knock out of *SWEET13* and *SWEET14* resulted in complete resistance^24^. Consistent with previous studies^19,30^, the dependence of African *Xoo* strains on *SWEET* gene expression to cause symptoms appears more complicated than anticipated. Alternatively, lesion length may not be strictly correlated with ecological fitness or disease severity, and variation in lesion length, particularly at lower value ranges, may be due to variant host defense responses to diverse pathogen lineages.

Broad resistance to bacterial blight at the *SWEET* promoters will not prevent adaptation of the pathogen, and the durability of this approach will depend on the ability of *Xoo* populations to adapt to recessive resistance alleles. As noted, TALe genes have limited diversity, and, when diversity is present, it comprises minor variations. We propose that creating polymorphisms that are larger than a single nucleotide change (ideally modifying the whole EBE) in the EBEs of *SWEET* genes is advisable. These changes will still capture minor TALe variants of the major, for example, for PthXo2 and AvrXa7/PthXo3. The promoter editing will also likely thwart TALe adaptation, assuming that ease of adaptation to new binding sites is inversely related to the number of novel nucleotides in the target sequence. EBEs must meet certain structural requirements for TALe binding within the available sequences of the susceptibility gene promoters. Another consideration is that breeders will likely combine these recessive resistance alleles with other locally effective resistance genes and these combinations will likely severely reduce disease pressure, further delaying strain adaptation.

A diagnostic kit that can both identify the promoter variants best suited to defeat the local pathogen population and monitor variant strain emergence is presented in an accompanying publication^24^. Breeders and pathologists can work together to provide selected lines to farmers, with an overall strategy of deploying multiple lines in a region to disfavor the evolution of novel strains.

Multiplex targeting using CRISPR–Cas9 has created the ability to simultaneously edit all EBEs present in any single rice line. However, this method is limited owing to the constraint of possible edits and problems with the delivery of editing complexes. For example, the most efficient genome editing is via DNA-mediated delivery, which confers regulatory concerns on edited plants. Genome editors such as CRISPR–Cpf1 (also called Cas12a, which allows for larger mutations), potent ribonucleoprotein (RNP) delivery and transgene-free genome editing may mitigate these limitations and improve the durability of disease resistance.

In conclusion, by using a combination of systematic analyses of diverse *Xoo* strains, an understanding of *SWEET* genes and genome editing, we were able to engineer broad-spectrum resistance in Kitaake and two mega varieties IR64 and Ciherang-Sub1. Genome editing can result in off-target effects and the transformation process often leads to mutations and somaclonal variation^31,32^, but on- and off-target analysis of rice genomes in NCBI with the Cas9 guide RNAs revealed that all the predicted off-target sites lack PAM sequences (an essential component for Cas9 recognition) and contain mismatches larger than 4 nucleotides to the guide sequences within the single guide RNA (sgRNA) genes (Supplementary Table 8). Furthermore, off-target mutations (if they are present), and other mutations derived from tissue culture and the regeneration process, will be eliminated in crosses during breeding. Full genome sequencing will be needed to identify possible off-target mutations and to ensure that the lines do not contain any T-DNA. Finally, our preliminary analysis of agronomic traits, while promising, is insufficient for direct deployment. Extensive field trials will be necessary to ensure performance of these edited resistant lines.

## Methods

### Plant material, bacterial strains, medium and growth conditions

Rice varieties used here were *Oryza sativa* L. ssp. *japonica* Kitaake^12^, and *Oryza sativa* L. ssp. *indica* IR24 (ref. ^12^), IR64 (ref. ^26^) and Ciherang-Sub1 (ref. ^28^). Rice plants were grown in growth chambers at 30 °C for a 12-h light period and at 28 °C for a 12-h dark period, with 60–75% relative humidity in the Yang laboratory, or under small-scale field environment in a screenhouse (28 °C ± 7 °C day and 23 °C ± 4 °C night; 80–85% relative humidity) at IRRI. *Escherichia coli* strains were grown in LB medium supplemented with appropriate antibiotics at 37 °C. *Agrobacterium tumefaciens* strains were grown at 30 °C in the dark. All *Xoo* strains were grown at 28 °C in TSA (10 g l^−1^ tryptone, 10 g l^−1^ sucrose and 1 g l^−1^ glutamic acid). Antibiotics were used at the following concentrations if required: 100 μg ml^−1^ ampicillin; 10 µg ml^−1^ cephalexin; 25 μg ml^−1^ chloramphenicol; 25 μg ml^−1^ kanamycin; 100 μg ml^−1^ spectinomycin; and 10 µg ml^−1^ tetracycline.

### *Xoo* genome sequencing and assembly

Total genomic DNA of *Xoo* strains was isolated using MagAttract HMW DNA Mini Kit (QIAGEN). DNA samples were sequenced using a single molecule real-time (SMRT-Pacbio) platform, P4-C6 chemistry. For each strain, at least two SMRT cells were used, generating around 180× coverage per genome. De novo assembly was conducted using the hierarchical assembly pipeline (HGAP) implemented in Canu v.1.5 software^33^. Raw PacBio reads were mapped against the resulting contigs using blasR aligner (https://github.com/PacificBiosciences/blasr) and corrections were conducted with the variant-caller software utilizing the arrow algorithm (https://github.com/PacificBiosciences/GenomicConsensus).

Genome annotation and gene prediction were conducted using the Prokka annotation pipeline^34^ and the NCBI Prokaryotic Genome Annotation (PGAP)^35^. Genome sequences were deposited in the GenBank under BioProjects PRJNA497307 and PRJNA497605. Additional reported and publicly available genomes in GenBank were retrieved and used for comparisons (Supplementary Table 1).

### Phylogenetic and comparative genomic analyses of *Xoo* strains

Phylogenetic analysis was conducted by identifying pan-genome SNPs in oligonucleotides of length *k* = 31 and generating parsimony trees on the basis of these SNPs, as implemented in the program kSNP (10.1093/bioinformatics/btv271).

### TALe content analysis in *Xoo*

TALe annotation was conducted using the AnnoTALE software^36^. Additional annotation and comparisons were performed using Artemis genome browser and Artemis Comparative Tool^37,38^. A neighbor-joining tree based on the alignments of repeat regions of TALes was obtained using DisTAL^39^. Target prediction for TALes was conducted with Talvez^40^; the target used was sequences in the promoter regions (1 kb upstream from the translation start site) of the rice *japonica* Nipponbare genome version deposited in Phytozome database V11 and of the *indica* variety IR64 (v.CSHL1.0)^41^.

### CRISPR–Cas9 design and constructs

For gene editing in Kitaake, the polycistronic tRNA–guide RNA (gRNA) system was used to generate multiple sgRNAs with different target sequences by flanking the sgRNAs with a tRNA precursor sequence^42^. Six intermediate vectors were constructed, pTLN-tgRNA-1 to T6, for six individual tRNA–gRNA units. A double-stranded DNA oligonucleotide for each site was produced by annealing two complementary oligonucleotides (24–25 nt) (Supplementary Table 9). Six double-stranded oligonucleotides were individually inserted into the *BsmB*I-digested pTLN-tgRNA-1 to T6 (Supplementary Fig. 7). The DNA sequence of positive clones was confirmed by Sanger sequencing (Supplementary Table 10). All six tRNA–gRNA units were transferred into another intermediate vector named pENTR4-U6.1P-ccdB using the Golden Gate ligation method (Supplementary Fig. 8). The gRNA cassettes were finally mobilized to pBY02-ZmUbiP-OsCas9 (Supplementary Fig. 9) by using Gateway LR Clonase (Thermo Fisher Scientific) as described^43^. For gene editing in IR64 and Ciherang-Sub1, four gRNA genes (IRS1132; Supplementary Fig. 9), targeting PthXo1 EBE in *SWEET11*, PthXo2 EBE in *SWEET13*, or TalC EBE or AvrXa7 EBE in *SWEET14*, were constructed as described^14^.

### Generation of rice mutant lines

*Agrobacterium* strains containing the respective CRISPR constructs were used for genome editing in Kitaake, IR64 and Ciherang-Sub1. Individual transformants were selected, propagated and regenerated into whole plants (T0) as described^44,45^. Leaf tissues collected from individual samples were homogenized in liquid nitrogen, and genomic DNA was isolated following the CTAB method. Initial screening for transformants (T0) was done via PCR of the phosphotransferase transgene (*hpt*) with primers HptF and HptR. Nuclease surveyor assay using Cel1 enzyme was employed in T0 plants to preselect lines with possible sequence alterations in the target regions. We selected 20 mono- and biallelic events for IR64 and sequenced the EBE regions using site-specific PCR amplicons obtained with primer pair 8NKpI-F5 and 8N3-R for *SWEET11*, primer pair 12N3-F1 and 12N3-R1 for *SWEET13* and primer pair 11N3-F2 and 11N3-R for *SWEET14*. To detect the CRISPR transgenes, paired primers OsCas9-F and OsCas9-R were used for Cas9, primers g8N3-F and g12N3-R for gRNA genes targeting *SWEET11* and *SWEET13*, and primers g11N3-F and g11N3-R for gRNA genes targeting *SWEET14*. These primer pairs were also used for selection of candidate mutant lines in the advanced generations (T1 to T2). Primer information is provided in Supplementary Table 9. Nineteen gRNA-gene-free and Cas9-free IR64 T1 plants were selected and further analyzed. Thirty plants for each of the 19 IR64 T2 lines were phenotyped for resistance to *Xoo* strains PXO339, PXO99 and PXO86, and target promoter regions of the candidate lines were sequenced to confirm mutations. Seeds of T2 plants were bulked (from 30 plants per line), phenotyped for *Xoo* resistance and analyzed for agronomic traits as described below. Seeds from 13 individual T3 plants were advanced to T4 on the basis of three criteria: (1) mutation type, (2) consistent resistance to the three *Xoo* strains and (3) seed count. For Ciherang-Sub1, we obtained seven independent transformation events. Eighteen Cas9-free T1 plants were further analyzed for resistance and amplicon sequencing as described above up to the T3 generation.

### Evaluation for agronomic traits of the rice mutant lines

We expected that mutant lines would show potential changes in agronomic traits owing to disruptive expression of the *SWEET* genes, off-target mutations across the genome or somaclonal variation. To assess the performance of the selected mutant lines, four agronomic characters in IR64-IRS1132 and Ciherang-Sub1-IRS1132 lines were assessed under paddy screenhouse conditions in a randomized block design experiment with three replications. At maturity, the plant height, panicle length, percentage of reproductive tillers (number of tillers with panicle/total number of panicles per plant) and percentage fertility (number of filled grains/total number of grains) were measured.

### Disease assays

Fully expanded leaves of rice plants (6–8 weeks old) were inoculated using a leaf-tip clipping method. *Xoo* stocks, preserved in a −80 °C freezer, were streaked out on TSA plates supplemented with appropriate antibiotics and incubated at 28 °C for 2–4 d. The cells were harvested from the plates and resuspended in sterilized distilled water (optical density at 600 nm of 0.5, ~10^8^ colony-forming units per milliliter). Scissor blades were immersed in *Xoo* suspension and used to clip about 2 cm from the leaf tip. The lesion lengths were measured 14 d after inoculation. Lesions were measured from each test plant. Lesion length measurements ≤ 5 cm were scored as resistant (R), 6–10 cm as moderately resistant (MR), 11–14 cm as moderately susceptible (MS) and ≥15 cm as susceptible (S). Three replications with about ten leaves from two to five plants per replicate were inoculated per strain.

### Statistical analysis

Data are plotted using BoxPlotR (http://shiny.chemgrid.org/boxplotr/) or are presented as mean ± s.e.m. Numbers of measurements are specified in figure legends or in graphs. One-way analysis of variance (ANOVA) was conducted on all measurements. Tukey’s honestly significant difference test was used for post-ANOVA pairwise tests for significance (set at *P* < 0.05), or, alternatively, the two-sided Dunnett’s test was used. Exact *P* values, statistical tests used and sample numbers (*n*) can be found in figure legends or in graphs.

### Reporting Summary

Further information on research design is available in the Nature Research Reporting Summary linked to this article.

## Online content

Any methods, additional references, Nature Research reporting summaries, source data, statements of code and data availability and associated accession codes are available at 10.1038/s41587-019-0267-z.

## Integrated supplementary information

Supplementary Figure 1 Independent origin of *SWEET*-inducing TAL effectors. Neighbor-joining tree based on DisTAL distances (based on alignments of TALe repeats) between all TALes from fully sequenced *Xoo* genomes. Each tip represents a single TALe. Color of the tips indicates country of isolation of the corresponding strain. Groups were defined by cutting the tree at a DisTAL distance of 4. Nodes corresponding to groups containing previously described *SWEET*-inducing TALes are highlighted in cyan with dashed squares. Two main *Xoo* lineages: *Xoo*^S^ and *Xoo*^F^ are indicated with bold lines. Blue bar indicates scale according to DisTAL distance.

Supplementary Figure 2 RVD sequences from TALes that target *SWEET* promoters. *SWEET*-binding predictions were made for all sequenced TALes from *Xoo*^S^ and *Xoo*^F^ strains. The known TALes (856) from 63 *Xoo* strains (Supplementary Table 1) were screened for binding to *SWEET* promoters using the software Talvez. In the regions predicted to bind to *SWEET* promoters, the amino acids responsible for binding (Repeat Variable Di-residues, RVDs) were identified, these are the 12^th^ and 13^th^ amino acids in each (~34 amino acids long) TALe repeat, and aligned with their predicted promoter Effector Binding Elements (EBEs). Each square represents an RVD, and the colors indicate the predicted relative binding affinity of each RVD to their matching nucleotide (1, orange, being a perfect match), as used in the program Talvez. Squares shown as “0” indicate the zero repeat, a non-canonical motif in the N-terminal region that is predicted to preferably bind to “T”. A unique sequence for each identified *SWEET*-inducing TALe variant is shown. The IDs to the left indicate representative strains that contain the RVD sequence. The letters in parenthesis (A to P) identify each variant. To the right of the figure are all other strains containing each variant as indicated. Negative numbers in the lower left indicate the distance of the shown sequence to the translation start site of the *SWEET* gene. Aberrant repeats were not looped out for these predictions. For simplicity, the two non-overlapping EBE regions in the *SWEET14* promoter are shown separately. PthXo2B and PthXo2C had higher prediction scores for the promoter of *SWEET13* in Nipponbare (v. MSU7), while PthXo2B and other PthXo2 versions are shown aligned to the IR64 (v. CSHL 1.0) *SWEET13* promoter allele. IDs of the genes shown in the corresponding genomes are: LOC_Os11g31190 (*SWEET14*_Nipponbare), LOC_Os12g29220 (*SWEET13*_Nipponbare), LOC_Os08g42350 (*SWEET11*_Nipponbare), maker-scaffold_793-pred_gff_Fgenesh-gene-0.10 (*SWEET13*_IR64).

Supplementary Figure 3 Virulence of *Xoo* strains on *O. sativa* ssp*. japonica* cv. Kitaake and *O. sativa* ssp*. indica* IR24. Lesion length caused by 10 *Xoo* strains in Kitaake (gray box) and IR24 (blue box). Each measurement was derived from young fully-expanded leaves of five rice plants. Center lines show the medians; box limits indicate 25th and 75th percentiles as determined by R software; data points (numbers below individual bars) are plotted as open circles (BoxPlotR; http://shiny.chemgrid.org/boxplotr/). Means for Kitaake are significantly different from means for IR24 (*p*<0.01). *P* values are shown under bars for IR24; one-way ANOVA. The experiment was repeated twice independently with similar results.

Supplementary Figure 4 The PthXo2 family of TAL effectors. **(a)** Alignment of the RVDs of PthXo2-related effectors from the seven variant *Xoo*^*S*^ strains, using the single amino acid residue code. RVDs of PthXo2 are shown on the last row for comparison. RVDs that differ from corresponding RVDs in PthXo2 are in red font. RVDs from aberrant repeats (36 aa) are shaded. **(b)** Adjusted alignment of the PthXo2 family members and the predicted EBEs of *indica* and *japonica* alleles of *SWEET13* in rice cultivars IR24 *(indica*) and Kitaake *(japonica*). Spaces are added to emphasize the similarities between the RVDs of the PthXo2-related effectors in relation to the corresponding EBEs. Stretches of identical RVDs are highlighted in blue.

Supplementary Figure 5 Virulence on different rice varieties by PthXo2 and PthXo2B. Lesion lengths in four rice lines caused by ME2 with or without TALe genes *pthXo2* and *pthXo2B*. The measurements were plotted using BoxPlotR (http://shiny.chemgrid.org/boxplotr/). Center lines show medians; box limits indicate 25th and 75th percentiles; data points (numbers of points shown below individual bars) as open circles. *P* values are shown in graph, one-way ANOVA. Experiments were repeated three times independently with comparable results.

Supplementary Figure 6 Guide RNA design. Six guide RNA genes were designed and constructed to mutate five known TALe EBEs in three *SWEET* promoters. Bold letters beneath shaded TALes are their target EBEs in *SWEET* promoters. Arrows indicate Cas9/gRNA cleavages sites at their respective binding sites.

Supplementary Figure 7 Sequence information of tRNA-gRNA constructs. Six gBlock fragments synthesized by IDT (Integrated DNA Technologies, Inc., Iowa, USA) were inserted into the vector pTLN by *Xba*I and *Xho*I (in green and box). The dots (…) are sequences in pTLN not shown. The orientation of individual components is in order of rice glycine tRNA (in blue), gRNA scaffold (in pink) and MS2 stem-loop (in orange). Overhangs (shaded in yellow) generated by digestion of *BsmB*I (underlined) are identical in six plasmids. However, overhands (shaded in gray) generated by digestion of *Bsa*I (double underlined) are designed for assembly of the tRNA-gRNA units through Golden Gate reaction.

Supplementary Figure 8 Sequence information of tRNA-gRNA recipient vector. The intermediate vector pENTR4-U6.1P-ccdB/chl constructed as the recipient vector for tRNA-gRNA contains two Gateway recombination sequences (in blue), rice U6 promoter (in green), two *Bsa*I (double underlined) sites. The cassettes of *ccdB* (in orange) and chl (chloramphenicol resistant) gene (in red) were constructed to facilitate the Golden Gate assembly of multiple tRNA-gRNA units.

Supplementary Figure 9 Map of the CRISPR/Cas9 construct IRS1132 for simultaneous editing of four EBEs in three *SWEET* gene promoters in rice.

Supplementary Figure 10 Agronomic traits in selected genome-edited mega variety lines compared to the parental controls. Performance of edited IR64-IRS1132 lines in the T3 generation (plant height, panicle length; % fertility and % reproductive tillers) relative to parental controls (IR64) control (n=15). Significant differences are denoted with asterisks (two-sided Dunnett’s test; *p* < 0.05 (*), *p* < 0.01 (**), *p* < 0.001 (***)). Micro-field experiments for agronomic trait assessments were conducted in a single season using Randomized Complete Block Design with three replicates. Center lines show medians; box limits indicate 25th and 75th percentiles as determined by R software.

## Supplementary information

Supplementary Materials Supplementary Figs. 1–10, Supplementary Tables 1–11 and Supplementary Notes 1 and 2

Reporting Summary

## Data Availability

Links to genome sequencing data are available in Supplementary Table 3 and at BioProjects PRJNA497605 and PRJNA497307. Materials will be made available for non-profit research under a material-transfer agreement (Supplementary Note 1) and a standard material-transfer agreement (SMTA) that complies with the International Treaty on Plant Genetic Resources for Food and Agriculture (IT PGRFA) for IR64 or Ciherang-Sub1 lines (Supplementary Note 2). We aim at obtaining freedom-to-operate for use by low-income farmers and will work with breeders to make the materials available to subsistence farmers. For commercial applications, license agreements will be negotiated that are compliant with national and international law regarding biosafety, import and export, exchange of biological resources, intellectual property and seed systems. Profits will be used to support dissemination to subsistence farmers. Edited IR64- and Ciherang-Sub1-based materials can be obtained from R.O., edited Kitaake lines from B.Y., translational reporter lines from W.B.F. Distribution of *Xoo* strains may be restricted, owing to regulations on *X. oryzae* as a Select Agent by the US government, because of the Nagoya protocol, or because some strains were donated from other groups; these groups should be contacted directly (Supplementary Table 11).
