# Supplementary figures and images for: Broad-spectrum resistance to bacterial blight in rice using genome editing
Source: Nat Biotechnol. 2019 Oct 28;37(11):1344–50. doi: 10.1038/s41587-019-0267-z (PMC6831514; doi:10.1038/s41587-019-0267-z)

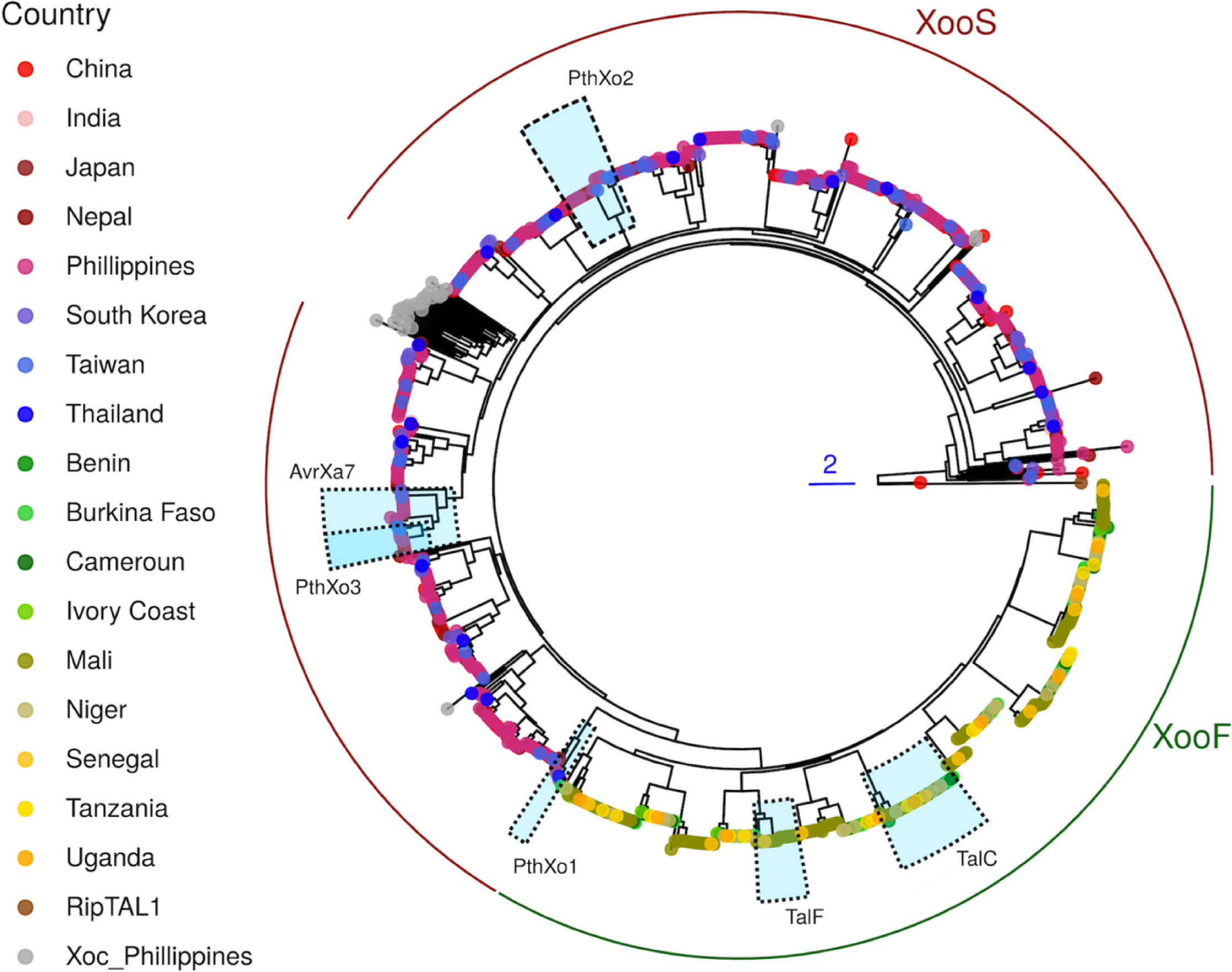

Supplement: Independent origin of SWEET-inducing TAL effectors. — Neighbor-joining tree based on DisTAL distances (based on alignments of TALe repeats) between all TALes from fully sequenced Xoo genomes. Each tip represents a single TALe. Color of the tips indicates country of isolation of the corresponding strain. Groups were defined by cutting the tree at a DisTAL distance of 4. Nodes corresponding to groups containing previously described SWEET-inducing TALes are highlighted in cyan with dashed squares. Two main Xoo lineages: XooS and XooF are indicated with bold lines. Blue bar indicates scale according to DisTAL distance. [file 41587_2019_267_Fig7_ESM.jpg]

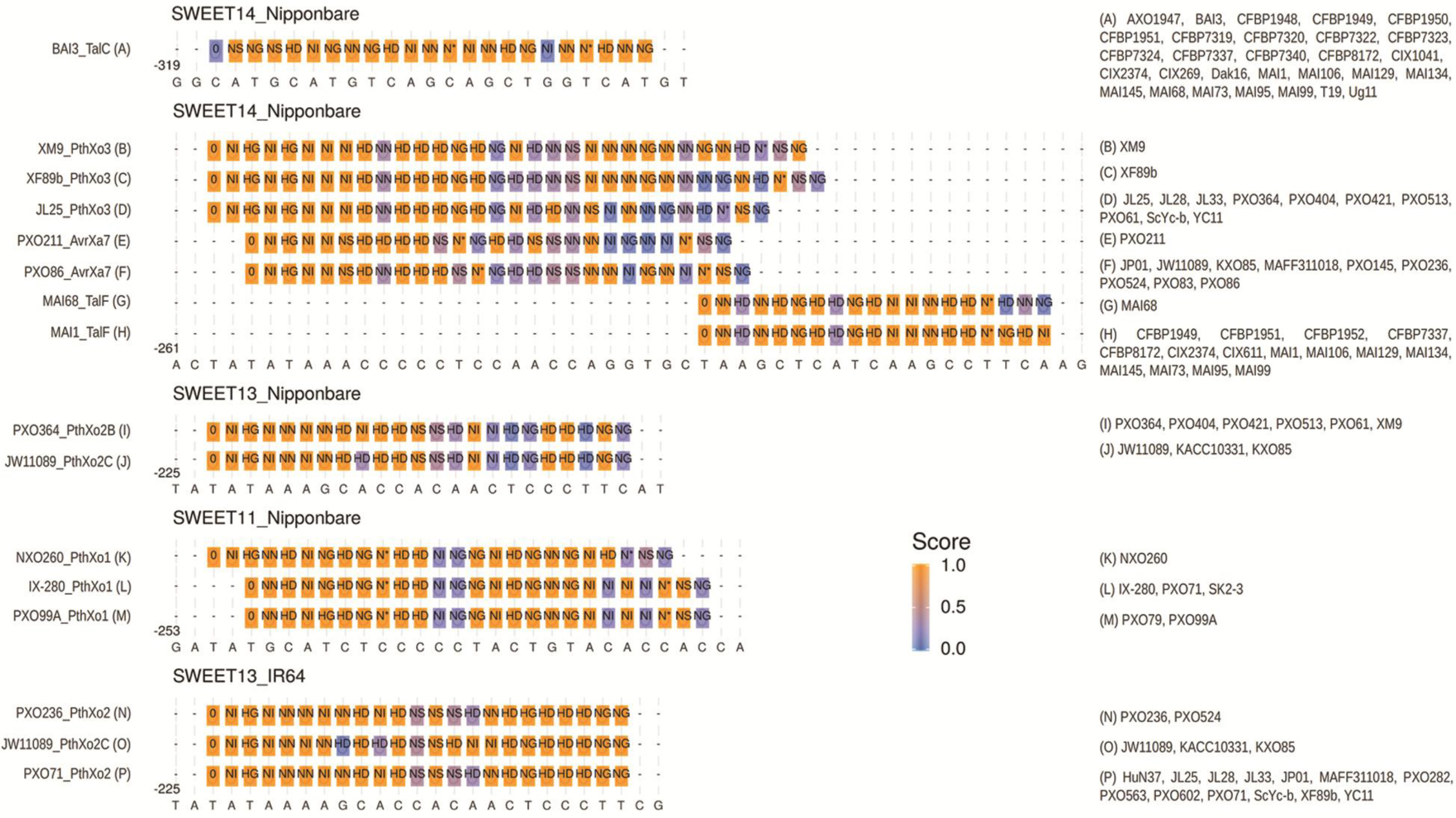

Supplement: RVD sequences from TALes that target SWEET promoters. — SWEET-binding predictions were made for all sequenced TALes from XooS and XooF strains. The known TALes (856) from 63 Xoo strains (Supplementary Table 1) were screened for binding to SWEET promoters using the software Talvez. In the regions predicted to bind to SWEET promoters, the amino acids responsible for binding (Repeat Variable Di-residues, RVDs) were identified, these are the 12th and 13th amino acids in each (~34 amino acids long) TALe repeat, and aligned with their predicted promoter Effector Binding Elements (EBEs). Each square represents an RVD, and the colors indicate the predicted relative binding affinity of each RVD to their matching nucleotide (1, orange, being a perfect match), as used in the program Talvez. Squares shown as “0” indicate the zero repeat, a non-canonical motif in the N-terminal region that is predicted to preferably bind to “T”. A unique sequence for each identified SWEET-inducing TALe variant is shown. The IDs to the left indicate representative strains that contain the RVD sequence. The letters in parenthesis (A to P) identify each variant. To the right of the figure are all other strains containing each variant as indicated. Negative numbers in the lower left indicate the distance of the shown sequence to the translation start site of the SWEET gene. Aberrant repeats were not looped out for these predictions. For simplicity, the two non-overlapping EBE regions in the SWEET14 promoter are shown separately. PthXo2B and PthXo2C had higher prediction scores for the promoter of SWEET13 in Nipponbare (v. MSU7), while PthXo2B and other PthXo2 versions are shown aligned to the IR64 (v. CSHL 1.0) SWEET13 promoter allele. IDs of the genes shown in the corresponding genomes are: LOC_Os11g31190 (SWEET14_Nipponbare), LOC_Os12g29220 (SWEET13_Nipponbare), LOC_Os08g42350 (SWEET11_Nipponbare), maker-scaffold_793-pred_gff_Fgenesh-gene-0.10 (SWEET13_IR64). [file 41587_2019_267_Fig8_ESM.jpg]

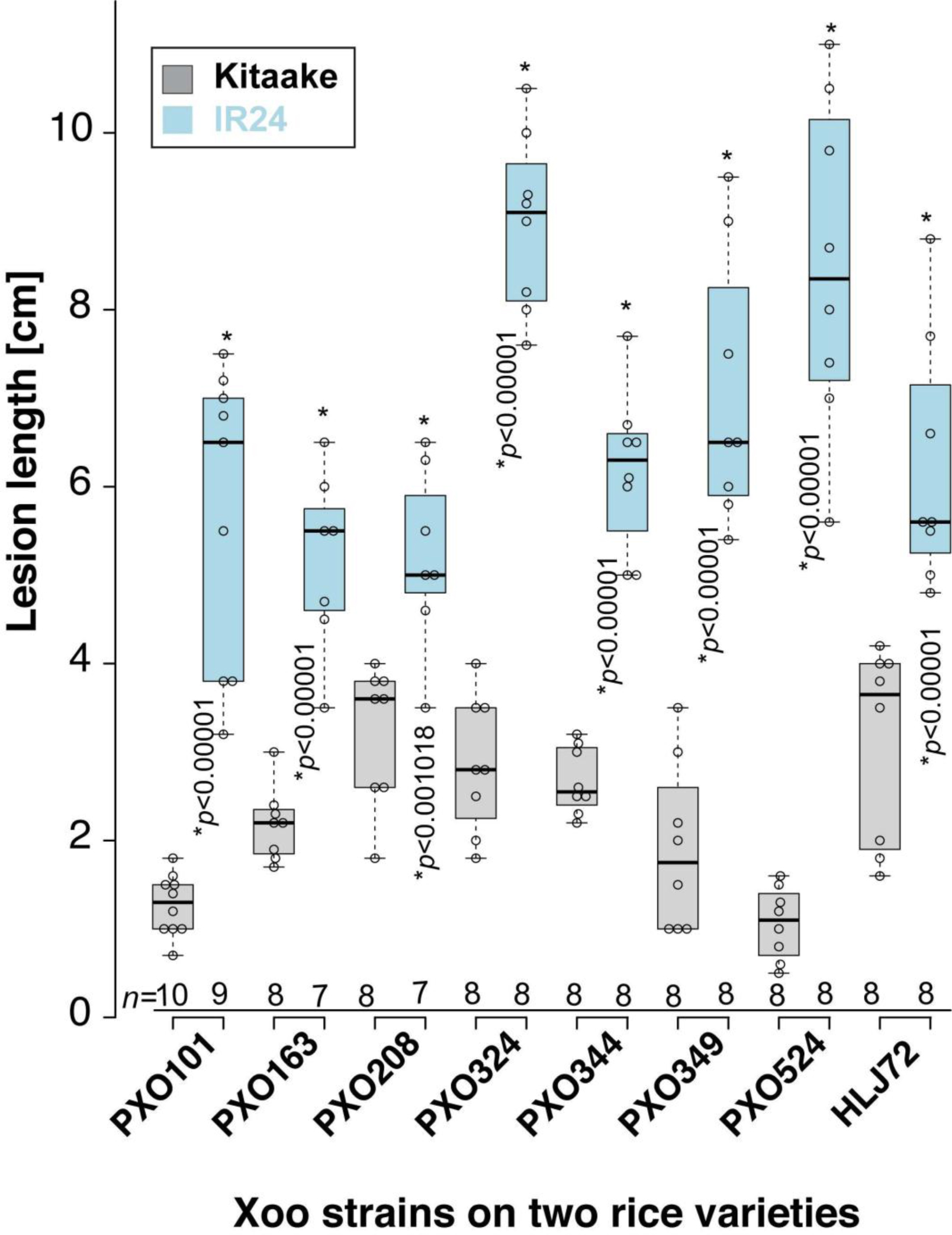

Supplement: Virulence of Xoo strains on O. sativa ssp. japonica cv. Kitaake and O. sativa ssp. indica IR24. — Lesion length caused by 10 Xoo strains in Kitaake (gray box) and IR24 (blue box). Each measurement was derived from young fully-expanded leaves of five rice plants. Center lines show the medians; box limits indicate 25th and 75th percentiles as determined by R software; data points (numbers below individual bars) are plotted as open circles (BoxPlotR; http://shiny.chemgrid.org/boxplotr/). Means for Kitaake are significantly different from means for IR24 (p<0.01). P values are shown under bars for IR24; one-way ANOVA. The experiment was repeated twice independently with similar results. [file 41587_2019_267_Fig9_ESM.jpg]

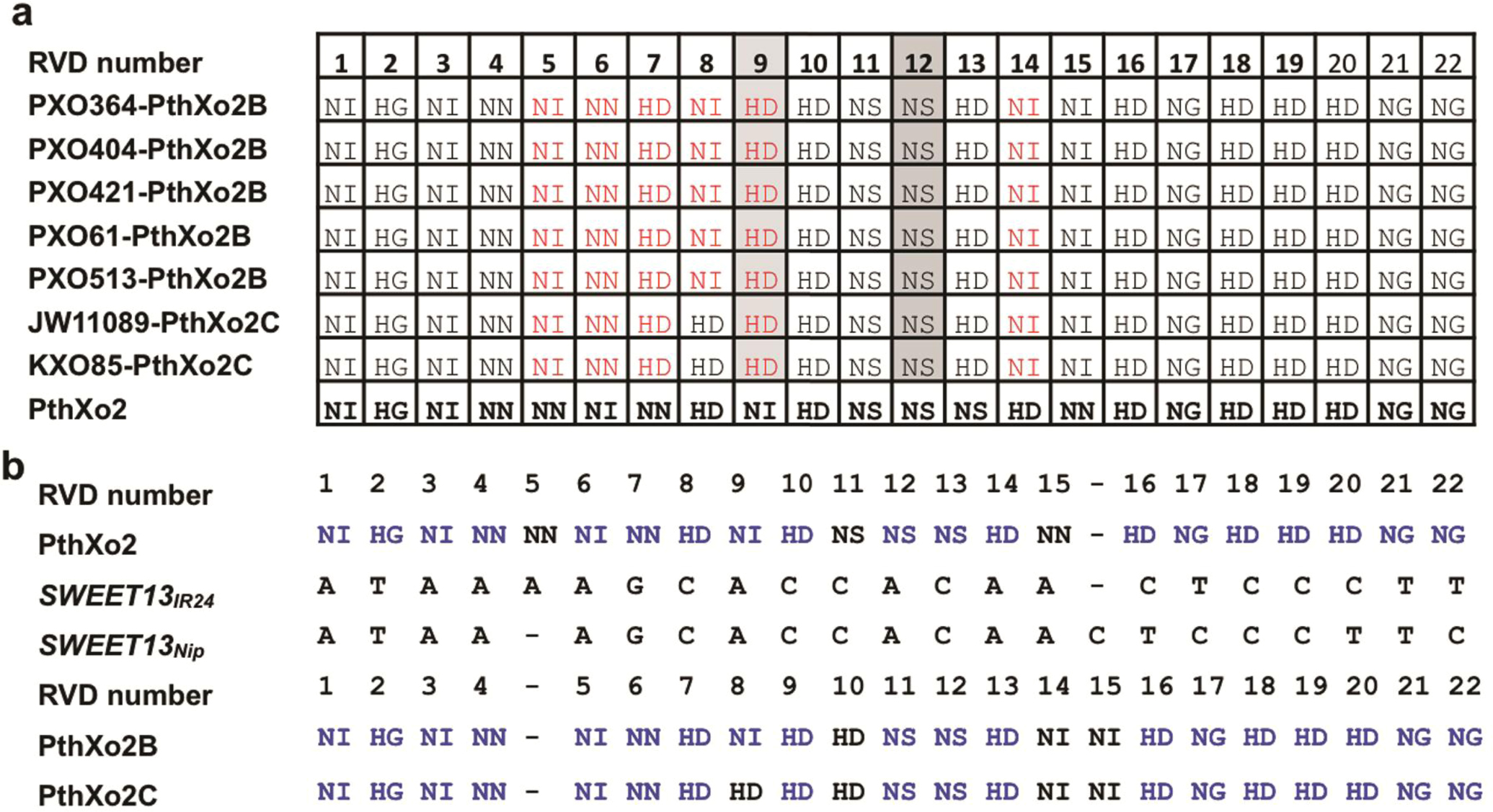

Supplement: The PthXo2 family of TAL effectors. — (a) Alignment of the RVDs of PthXo2-related effectors from the seven variant XooS strains, using the single amino acid residue code. RVDs of PthXo2 are shown on the last row for comparison. RVDs that differ from corresponding RVDs in PthXo2 are in red font. RVDs from aberrant repeats (36 aa) are shaded. (b) Adjusted alignment of the PthXo2 family members and the predicted EBEs of indica and japonica alleles of SWEET13 in rice cultivars IR24 (indica) and Kitaake (japonica). Spaces are added to emphasize the similarities between the RVDs of the PthXo2-related effectors in relation to the corresponding EBEs. Stretches of identical RVDs are highlighted in blue. [file 41587_2019_267_Fig10_ESM.jpg]

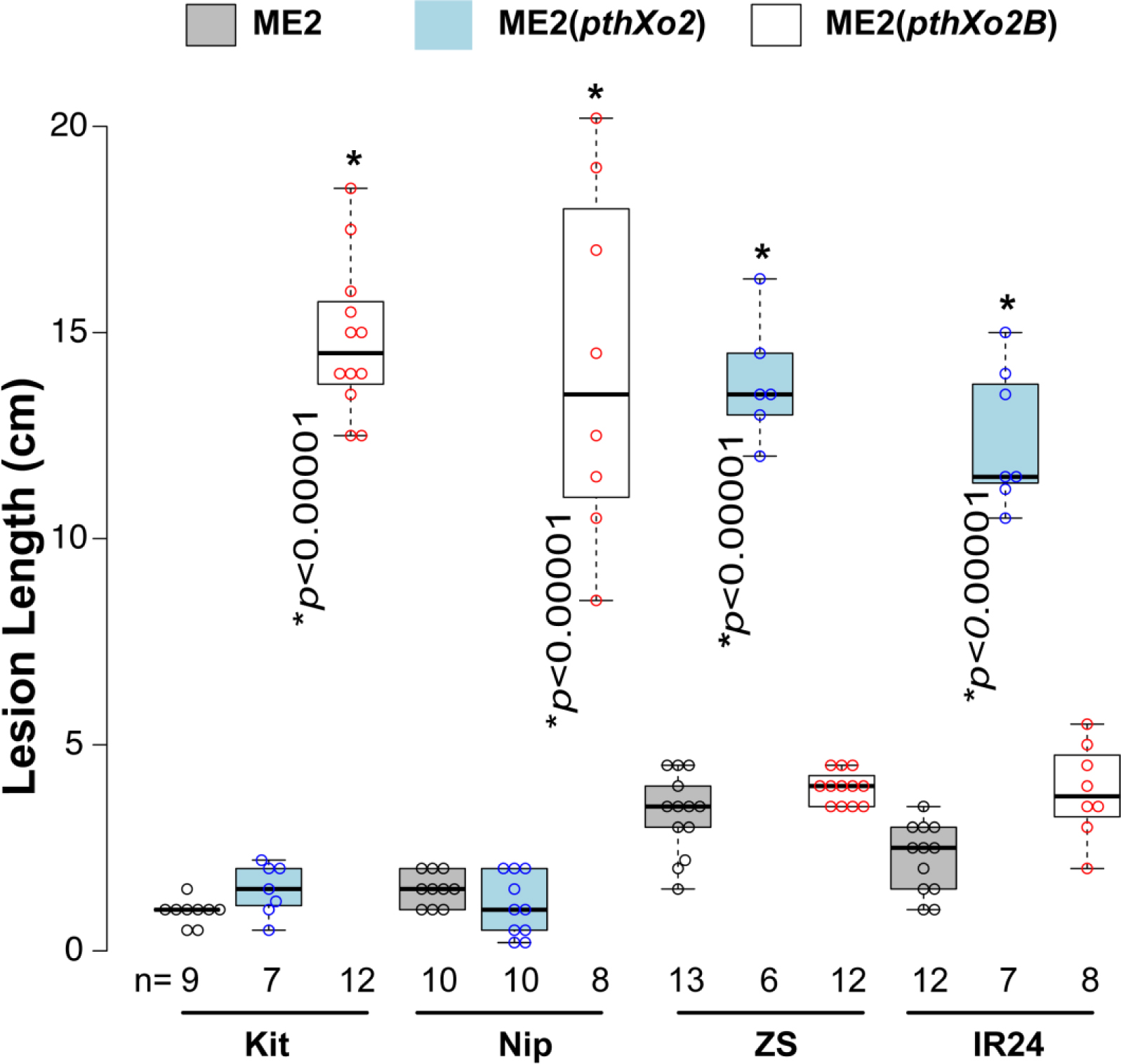

Supplement: Virulence on different rice varieties by PthXo2 and PthXo2B. — Lesion lengths in four rice lines caused by ME2 with or without TALe genes pthXo2 and pthXo2B. The measurements were plotted using BoxPlotR (http://shiny.chemgrid.org/boxplotr/). Center lines show medians; box limits indicate 25th and 75th percentiles; data points (numbers of points shown below individual bars) as open circles. P values are shown in graph, one-way ANOVA. Experiments were repeated three times independently with comparable results. [file 41587_2019_267_Fig11_ESM.jpg]

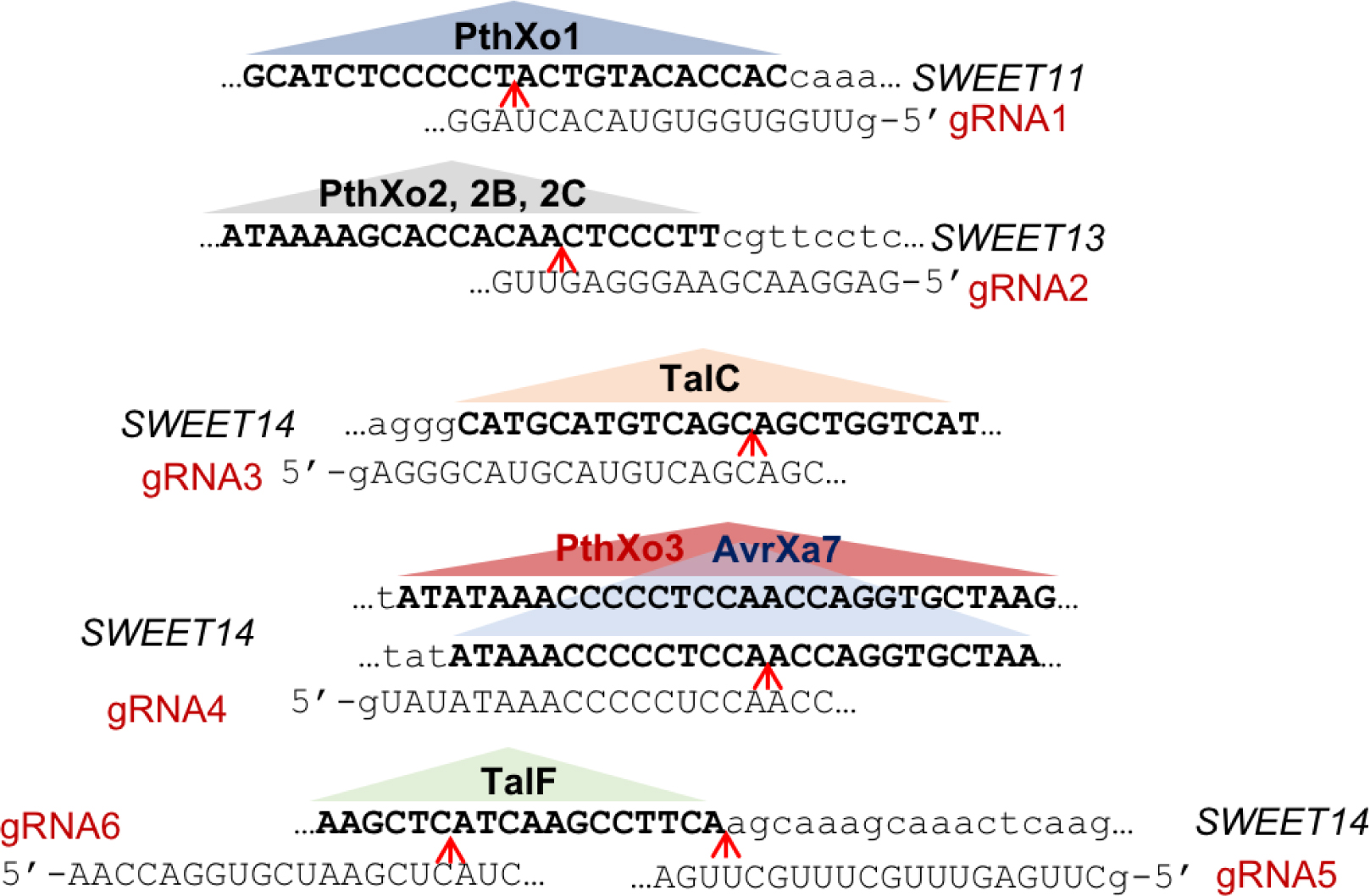

Supplement: Guide RNA design. — Six guide RNA genes were designed and constructed to mutate five known TALe EBEs in three SWEET promoters. Bold letters beneath shaded TALes are their target EBEs in SWEET promoters. Arrows indicate Cas9/gRNA cleavages sites at their respective binding sites. [file 41587_2019_267_Fig12_ESM.jpg]

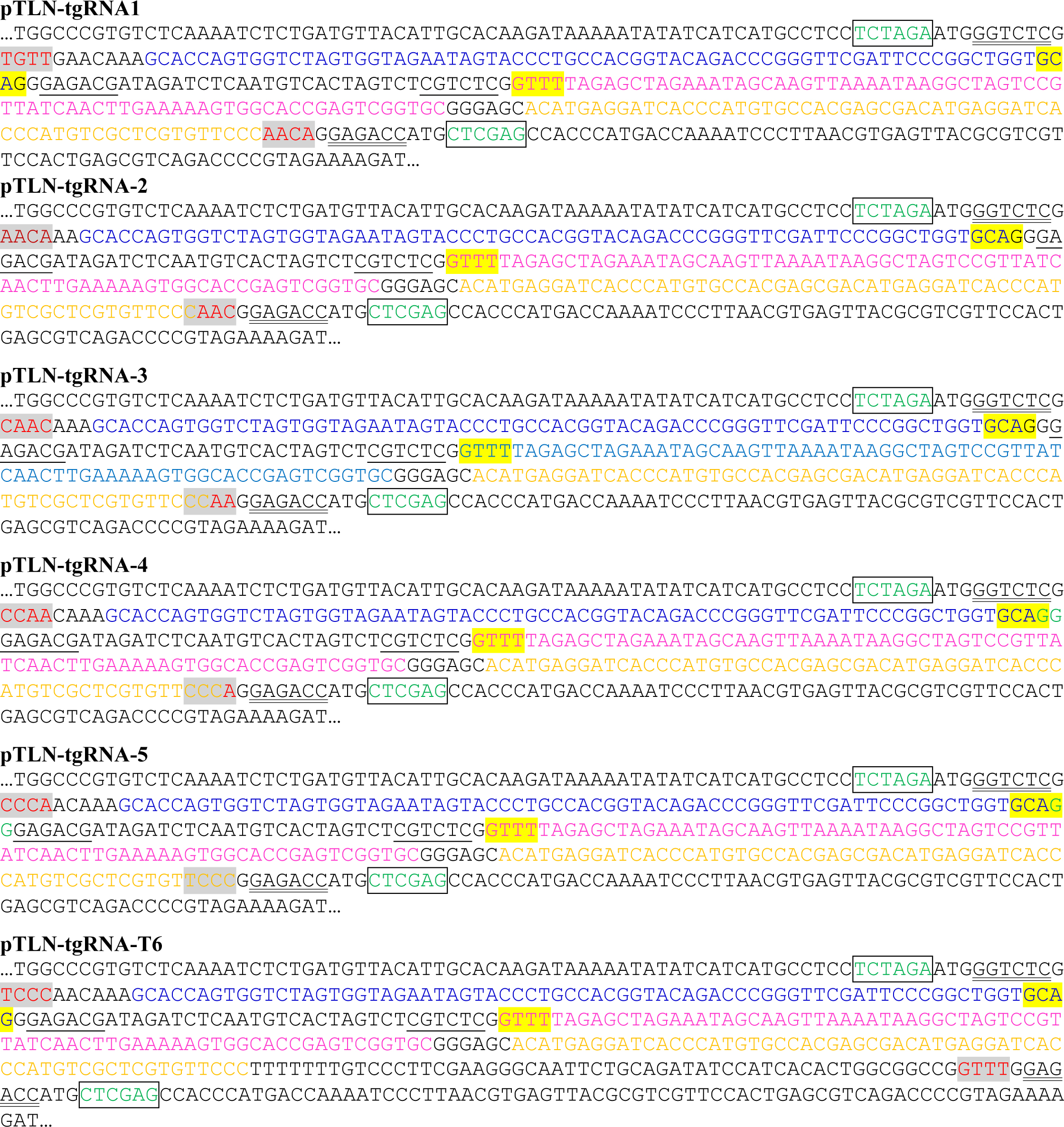

Supplement: Sequence information of tRNA-gRNA constructs. — Six gBlock fragments synthesized by IDT (Integrated DNA Technologies, Inc., Iowa, USA) were inserted into the vector pTLN by XbaI and XhoI (in green and box). The dots (…) are sequences in pTLN not shown. The orientation of individual components is in order of rice glycine tRNA (in blue), gRNA scaffold (in pink) and MS2 stem-loop (in orange). Overhangs (shaded in yellow) generated by digestion of BsmBI (underlined) are identical in six plasmids. However, overhands (shaded in gray) generated by digestion of BsaI (double underlined) are designed for assembly of the tRNA-gRNA units through Golden Gate reaction. [file 41587_2019_267_Fig13_ESM.jpg]

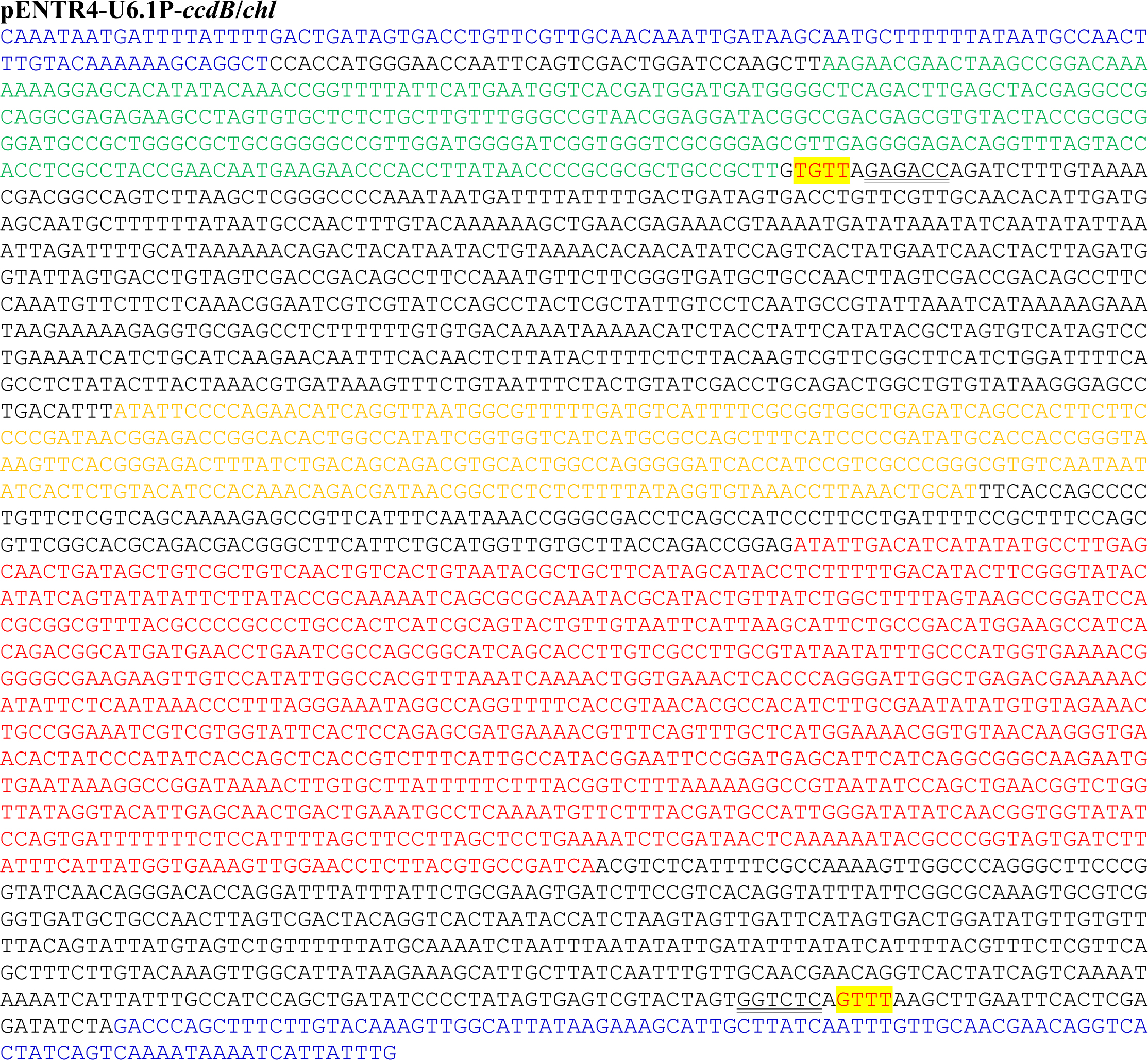

Supplement: Sequence information of tRNA-gRNA recipient vector. — The intermediate vector pENTR4-U6.1P-ccdB/chl constructed as the recipient vector for tRNA-gRNA contains two Gateway recombination sequences (in blue), rice U6 promoter (in green), two BsaI (double underlined) sites. The cassettes of ccdB (in orange) and chl (chloramphenicol resistant) gene (in red) were constructed to facilitate the Golden Gate assembly of multiple tRNA-gRNA units. [file 41587_2019_267_Fig14_ESM.jpg]

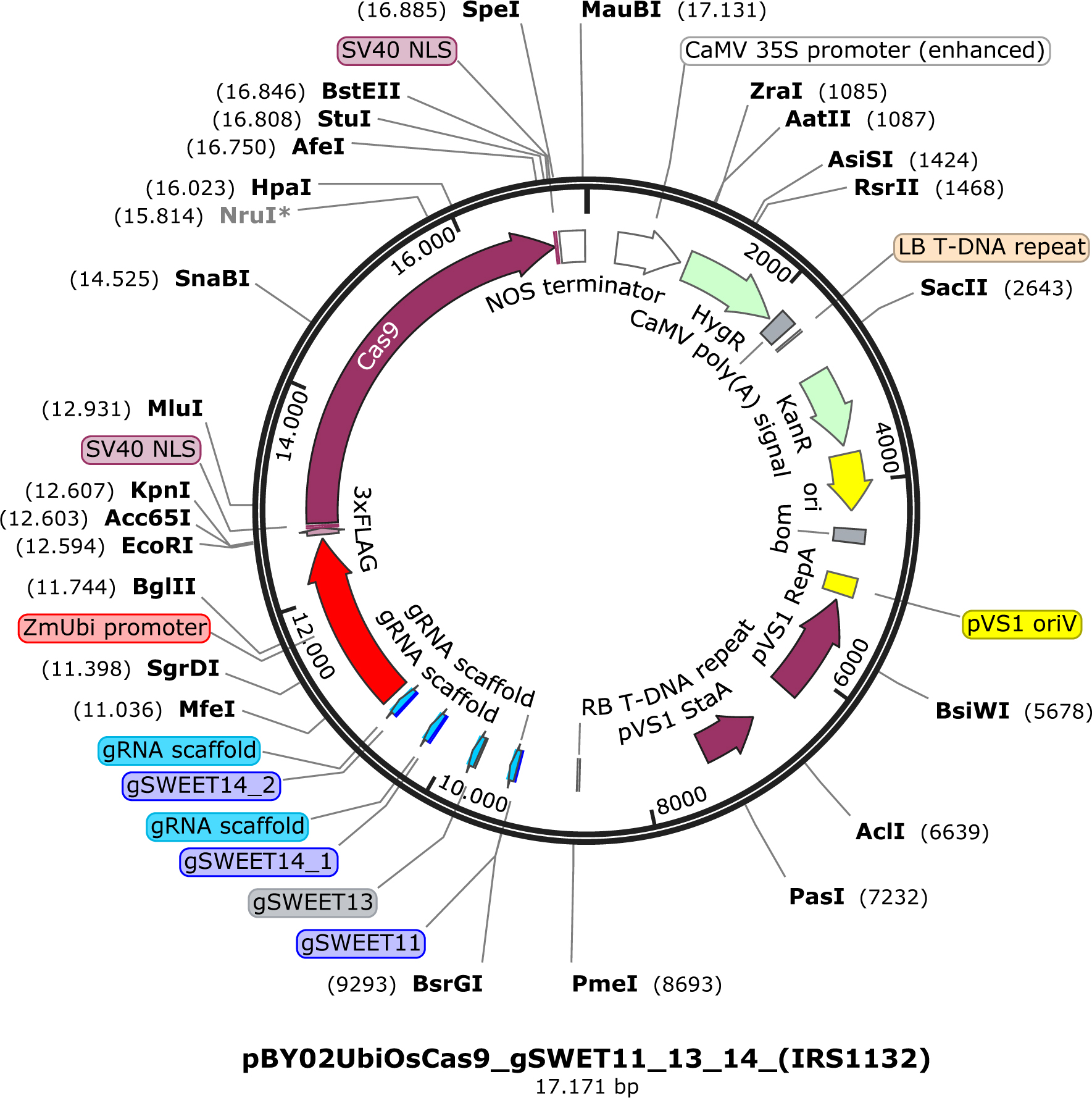

Supplement: Supplementary file 9 — Map of the CRISPR/Cas9 construct IRS1132 for simultaneous editing of four EBEs in three SWEET gene promoters in rice. [file 41587_2019_267_Fig15_ESM.jpg]

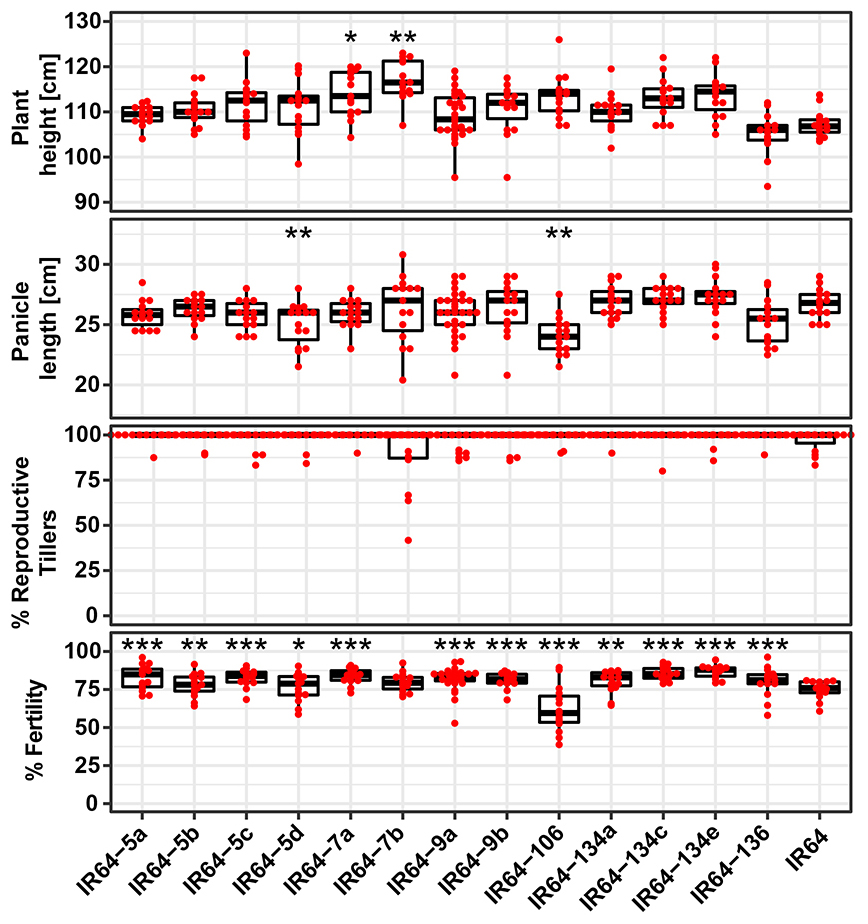

Supplement: Agronomic traits in selected genome-edited mega variety lines compared to the parental controls. — Performance of edited IR64-IRS1132 lines in the T3 generation (plant height, panicle length; % fertility and % reproductive tillers) relative to parental controls (IR64) control (n=15). Significant differences are denoted with asterisks (two-sided Dunnett’s test; p < 0.05 (*), p < 0.01 (**), p < 0.001 (***)). Micro-field experiments for agronomic trait assessments were conducted in a single season using Randomized Complete Block Design with three replicates. Center lines show medians; box limits indicate 25th and 75th percentiles as determined by R software. [file 41587_2019_267_Fig16_ESM.jpg]
