# Supplementary material for: Broad-spectrum resistance to bacterial blight in rice using genome editing
Source: Nat Biotechnol. 2019 Oct 28;37(11):1344–50. doi: 10.1038/s41587-019-0267-z (PMC6831514; doi:10.1038/s41587-019-0267-z)
Supplement: Supplementary file 11 — Supplementary Figs. 1–10, Supplementary Tables 1–11 and Supplementary Notes 1 and 2 [file 41587_2019_267_MOESM1_ESM.pdf]

nature biotechnology

SUPPLEMENTARY INFORMATION
https://doi.org/10.1038/s41587-019-0267-z

In the format provided by the authors and unedited.

Broad-spectrum resistance to bacterial blight in rice using genome editing

Ricardo Oliva[1,12*], Chonghui Ji[2,12], Genelou Atienza-Grande[1,10,12], José C. Huguet-Tapia[3,12], Alvaro Perez-Quintero[4,11,12], Ting Li[5], Joon-Seob Eom[6], Chenhao Li[2], Hanna Nguyen[1], Bo Liu[2], Florence Auguy[4], Coline Sciallano[4], Van T. Luu[6], Gerbert S. Dossa[7], Sébastien Cunnac[4], Sarah M. Schmidt[6], Inez H. Slamet-Loedin[1], Casiana Vera Cruz[1], Boris Szurek[4], Wolf B. Frommer[6,8*], Frank F. White[3] and Bing Yang[2,9*]

[1]International Rice Research Institute, Metro Manila, Philippines. [2]Division of Plant Sciences, Bond Life Sciences Center, University of Missouri, Columbia, MO, USA. [3]Department of Plant Pathology, University of Florida, Gainesville, FL, USA. [4]IRD, CIRAD, Université Montpellier, IPME, Montpellier, France. [5]Department of Genetics, Development and Cell Biology, Iowa State University, Ames, IA, USA. [6]Institute for Molecular Physiology and Cluster of Excellence on Plant Sciences (CEPLAS), Heinrich Heine Universität Düsseldorf and Max Planck Institute for Plant Breeding Research, Köln, Germany. [7]Erfurt University of Applied Sciences, Erfurt, Germany. [8]Institute of Transformative Bio-Molecules (WPI-ITbM), Nagoya University, Aichi, Japan. [9]Donald Danforth Plant Science Center, St. Louis, MO, USA. [10]Present address: College of Agriculture and Food Science, University of the Philippines Los Baños, Los Baños, Philippines. [11]Present address: Department of Bioagricultural Sciences and Pest Management, Colorado State University, Fort Collins, CO, USA. [12]These authors contributed equally: Ricardo Oliva, Chonghui Ji, Genelou Atienza-Grande, José C. Huguet-Tapia, Alvaro Perez-Quintero.
*e-mail: r.oliva@irri.org; frommew@hhu.de; yangbi@missouri.edu

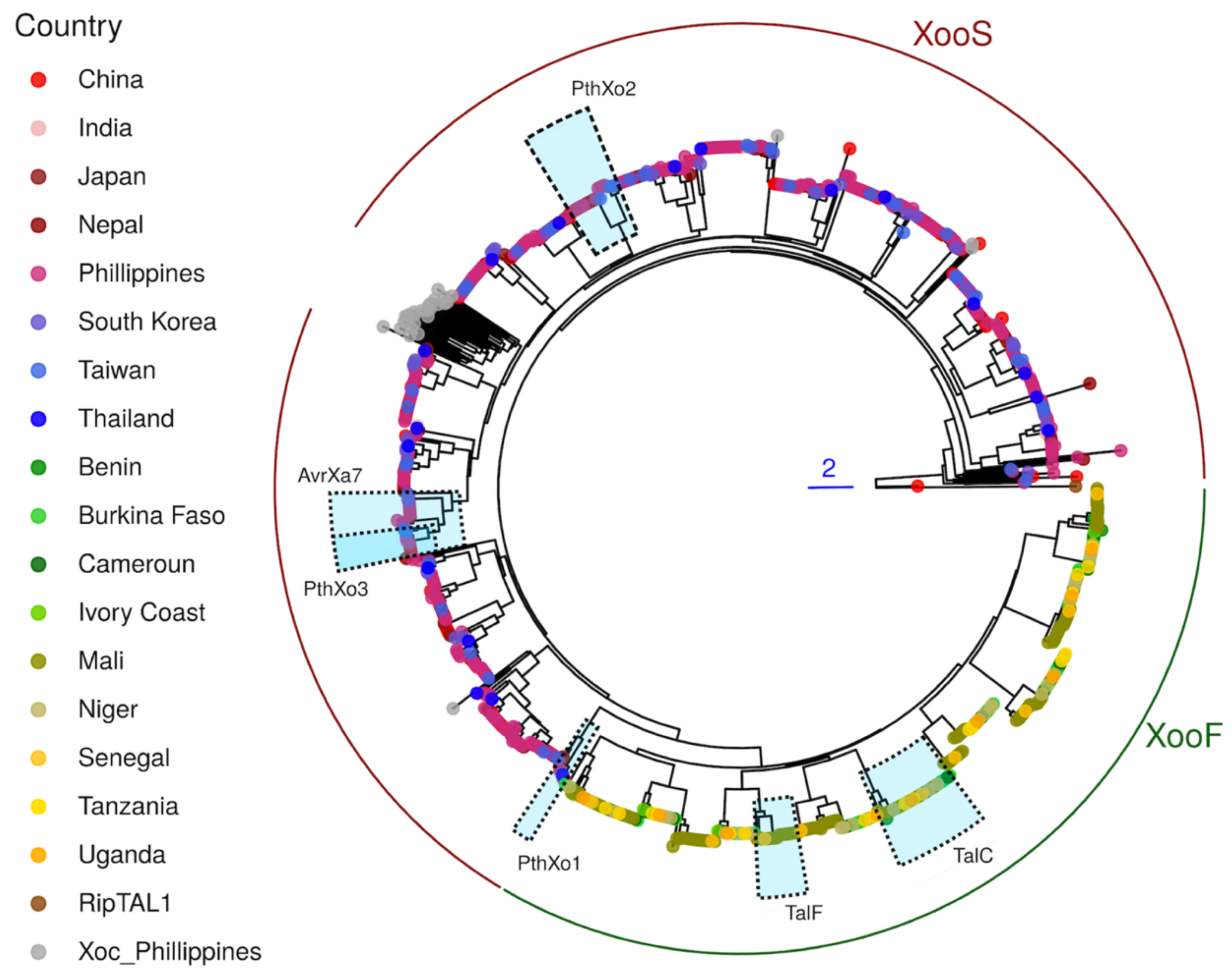

Supplementary Figure 1

Independent origin of *SWEET*-inducing TAL effectors

Neighbor-joining tree based on DisTAL distances (based on alignments of TALe repeats) between all TALes from fully sequenced *Xoo* genomes. Each tip represents a single TALe. Color of the tips indicates country of isolation of the corresponding strain. Groups were defined by cutting the tree at a DisTAL distance of 4. Nodes corresponding to groups containing previously described *SWEET*-inducing TALes are highlighted in cyan with dashed squares. Two main *Xoo* lineages: *Xoo*^{S} and *Xoo*^{F} are indicated with bold lines. Blue bar indicates scale according to DisTAL distance.

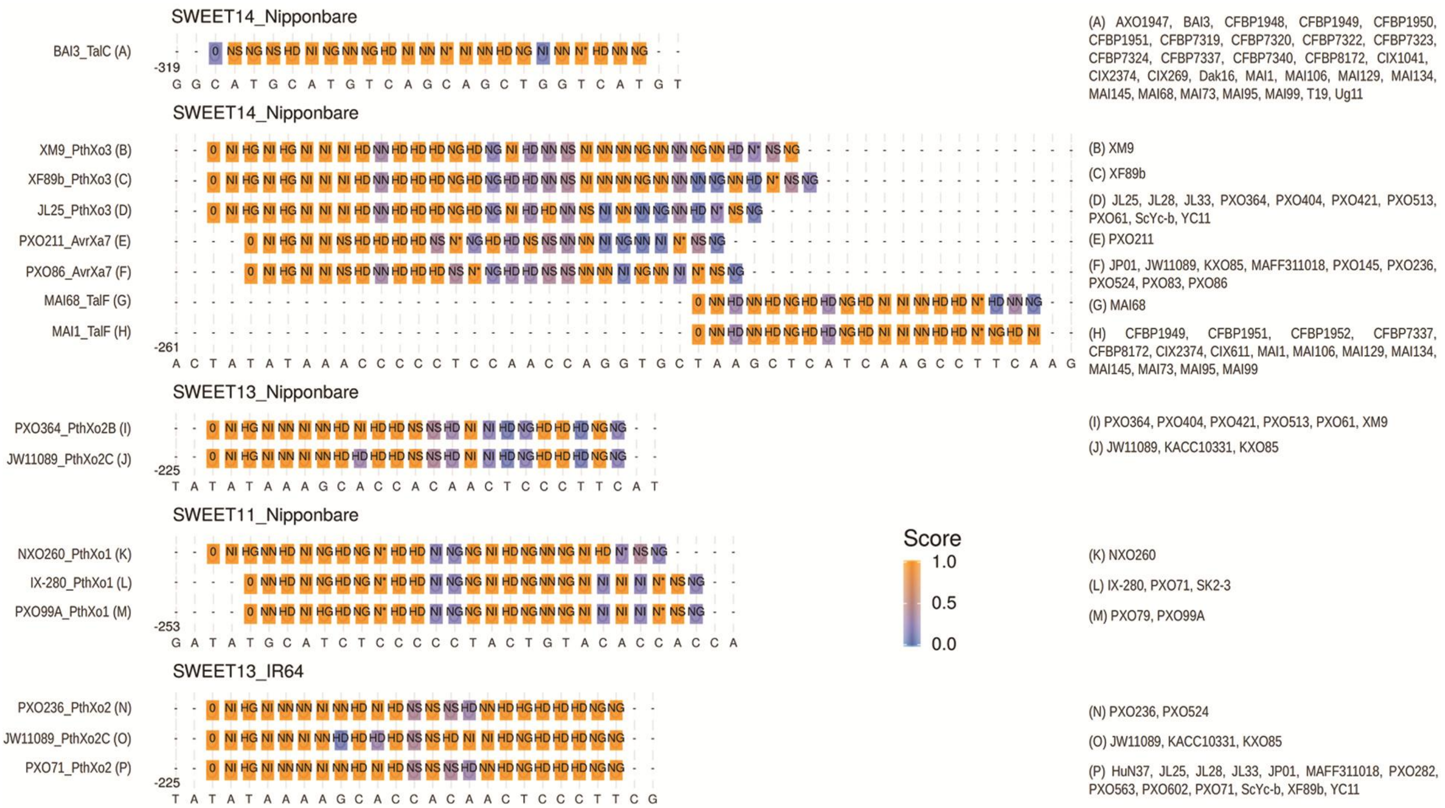

Supplementary Figure 2

RVD sequences from TALes that target *SWEET* promoters

SWEET-binding predictions were made for all sequenced TALes from *Xoo*^{S} and *Xoo*^{F} strains. The known TALes (856) from 63 *Xoo* strains (Supplementary Table 1) were screened for binding to *SWEET* promoters using the software Talvez. In the regions predicted to bind to *SWEET* promoters, the amino acids responsible for binding (Repeat Variable Di-residues, RVDs) were identified, these are the 12th and 13th amino acids in each (~34 amino acids long) TALe repeat, and aligned with their predicted promoter Effector Binding Elements (EBEs). Each square represents an RVD, and the colors indicate the predicted relative binding affinity of each RVD to their matching nucleotide (1, orange, being a perfect match), as used in the program Talvez. Squares shown as “0” indicate the zero repeat, a non-canonical motif in the N-terminal region that is predicted to preferably bind to “T”. A unique sequence for each identified *SWEET*-inducing TALe variant is shown. The IDs to the left indicate representative strains that contain the RVD sequence. The letters in parenthesis (A to P) identify each variant. To the right of the figure are all other strains containing each variant as indicated. Negative numbers in the lower left indicate the distance of the shown sequence to the translation start site of the *SWEET* gene. Aberrant repeats were not looped out for these predictions. For simplicity, the two non-overlapping EBE regions in the *SWEET14* promoter are shown separately. PthXo2B and PthXo2C had higher prediction scores for the promoter of *SWEET13* in Nipponbare (v. MSU7), while PthXo2B and other PthXo2 versions are shown aligned to the IR64 (v. CSHL 1.0) *SWEET13* promoter allele. IDs of the genes shown in the corresponding genomes are: LOC_Os11g31190 (*SWEET14*_Nipponbare), LOC_Os12g29220 (*SWEET13*_Nipponbare), LOC_Os08g42350 (*SWEET11*_Nipponbare), maker-scaffold_793-pred_gff_Fgenesh-gene-0.10 (*SWEET13*_IR64).

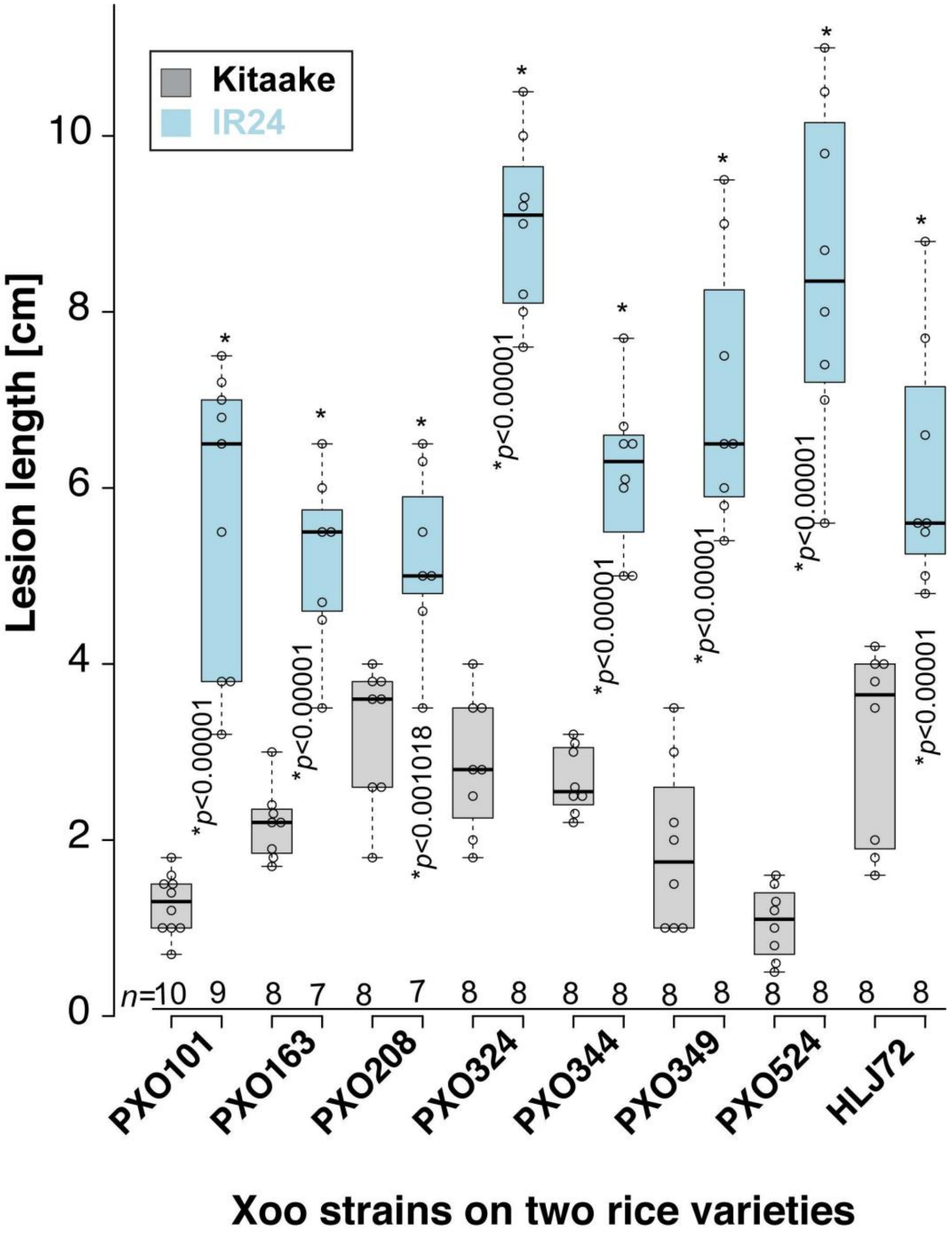

Supplementary Figure 3

Virulence of *Xoo* strains on *O. sativa* ssp. *japonica* cv. Kitaake and *O. sativa* ssp. *indica* IR24

Lesion length caused by 10 *Xoo* strains in Kitaake (grey box) and IR24 (blue box). Each measurement was derived from young fully-expanded leaves of five rice plants. Center lines show the medians; box limits indicate 25th and 75th percentiles as determined by R software; data points (numbers below individual bars) are plotted as open circles (BoxPlotR; http://shiny.chemgrid.org/boxplotr/). Means for Kitaake are significantly different from means for IR24 (p<0.01). P values are shown under bars for IR24; one-way ANOVA. The experiment was repeated twice independently with similar results.

a

RVD number	1	2	3	4	5	6	7	8	9	10	11	12	13	14	15	16	17	18	19	20	21	22
PXO364-PthXo2B	NI	HG	NI	NN	NI	NN	HD	NI	HD	HD	NS	NS	HD	NI	NI	HD	NG	HD	HD	HD	NG	NG
PXO404-PthXo2B	NI	HG	NI	NN	NI	NN	HD	NI	HD	HD	NS	NS	HD	NI	NI	HD	NG	HD	HD	HD	NG	NG
PXO421-PthXo2B	NI	HG	NI	NN	NI	NN	HD	NI	HD	HD	NS	NS	HD	NI	NI	HD	NG	HD	HD	HD	NG	NG
PXO61-PthXo2B	NI	HG	NI	NN	NI	NN	HD	NI	HD	HD	NS	NS	HD	NI	NI	HD	NG	HD	HD	HD	NG	NG
PXO513-PthXo2B	NI	HG	NI	NN	NI	NN	HD	NI	HD	HD	NS	NS	HD	NI	NI	HD	NG	HD	HD	HD	NG	NG
JW11089-PthXo2C	NI	HG	NI	NN	NI	NN	HD	HD	HD	HD	NS	NS	HD	NI	NI	HD	NG	HD	HD	HD	NG	NG
KXO85-PthXo2C	NI	HG	NI	NN	NI	NN	HD	HD	HD	HD	NS	NS	HD	NI	NI	HD	NG	HD	HD	HD	NG	NG
PthXo2	NI	HG	NI	NN	NN	NI	NN	HD	NI	HD	NS	NS	NS	HD	NN	HD	NG	HD	HD	HD	NG	NG

b

RVD number	1	2	3	4	5	6	7	8	9	10	11	12	13	14	15	-	16	17	18	19	20	21	22
PthXo2	NI	HG	NI	NN	NN	NI	NN	HD	NI	HD	NS	NS	NS	HD	NN	-	HD	NG	HD	HD	HD	NG	NG
SWEET13$_{IR24}$	A	T	A	A	A	A	G	C	A	C	C	A	C	A	A	-	C	T	C	C	C	T	T
SWEET13$_{Nip}$	A	T	A	A	-	A	G	C	A	C	C	A	C	A	A	C	T	C	C	C	T	T	C
RVD number	1	2	3	4	-	5	6	7	8	9	10	11	12	13	14	15	16	17	18	19	20	21	22
PthXo2B	NI	HG	NI	NN	-	NI	NN	HD	NI	HD	HD	NS	NS	HD	NI	NI	HD	NG	HD	HD	HD	NG	NG
PthXo2C	NI	HG	NI	NN	-	NI	NN	HD	HD	HD	HD	NS	NS	HD	NI	NI	HD	NG	HD	HD	HD	NG	NG

Supplementary Figure 4

The PthXo2 family of TAL effectors

(a) Alignment of the RVDs of PthXo2-related effectors from the seven variant *Xoo*^{S} strains, using the single amino acid residue code. RVDs of PthXo2 are shown on the last row for comparison. RVDs that differ from corresponding RVDs in PthXo2 are in red font. RVDs from aberrant repeats (36 aa) are shaded. **(b)** Adjusted alignment of the PthXo2 family members and the predicted EBEs of *indica* and *japonica* alleles of *SWEET13* in rice cultivars IR24 (*indica*) and Kitaake (*japonica*). Spaces are added to emphasize the similarities between the RVDs of the PthXo2-related effectors in relation to the corresponding EBEs. Stretches of identical RVDs are highlighted in blue.

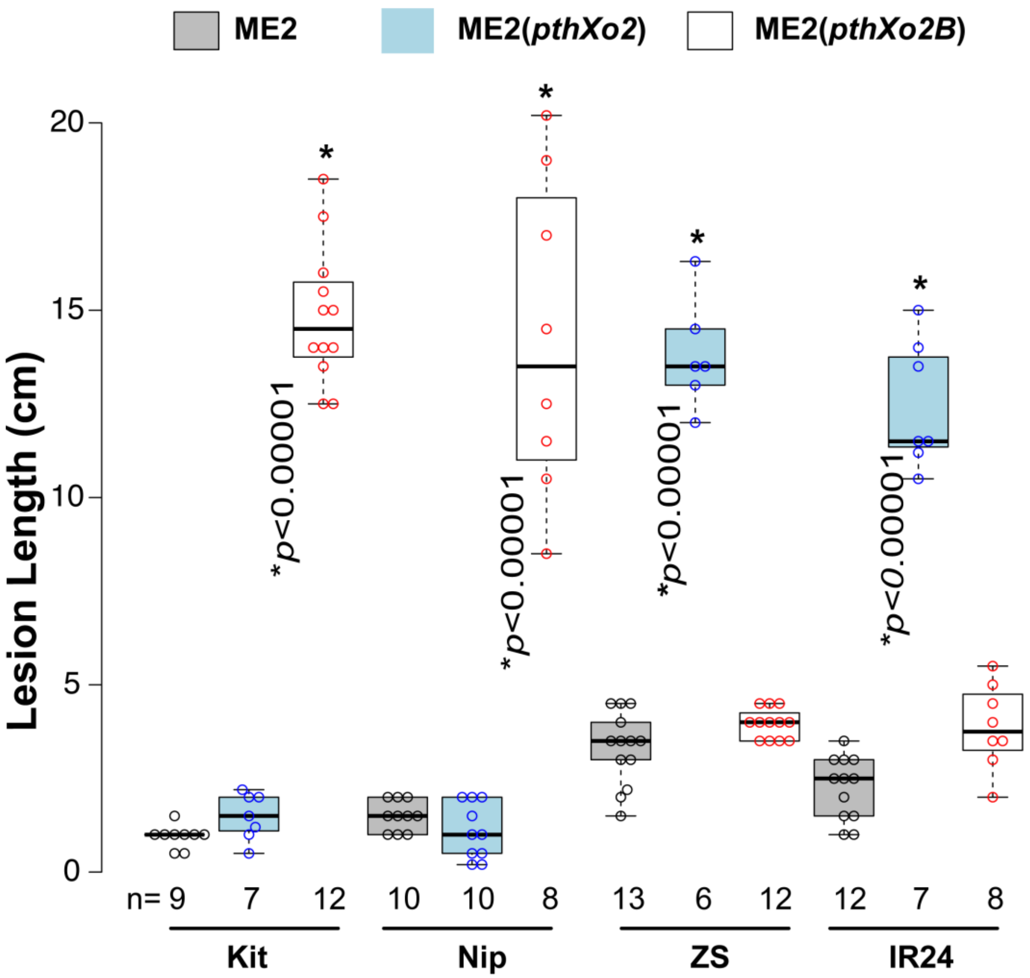

Supplementary Figure 5

Virulence on different rice varieties by PthXo2 and PthXo2B

Lesion lengths in four rice lines caused by ME2 with or without TALe genes *pthXo2* and *pthXo2B*. The measurements were plotted using BoxPlotR (http://shiny.chemgrid.org/boxplotr/). Center lines show medians; box limits indicate 25th and 75th percentiles; data points (numbers of points shown below individual bars) as open circles. *P* values are shown in graph, one-way ANOVA. Experiments were repeated three times independently with comparable results.

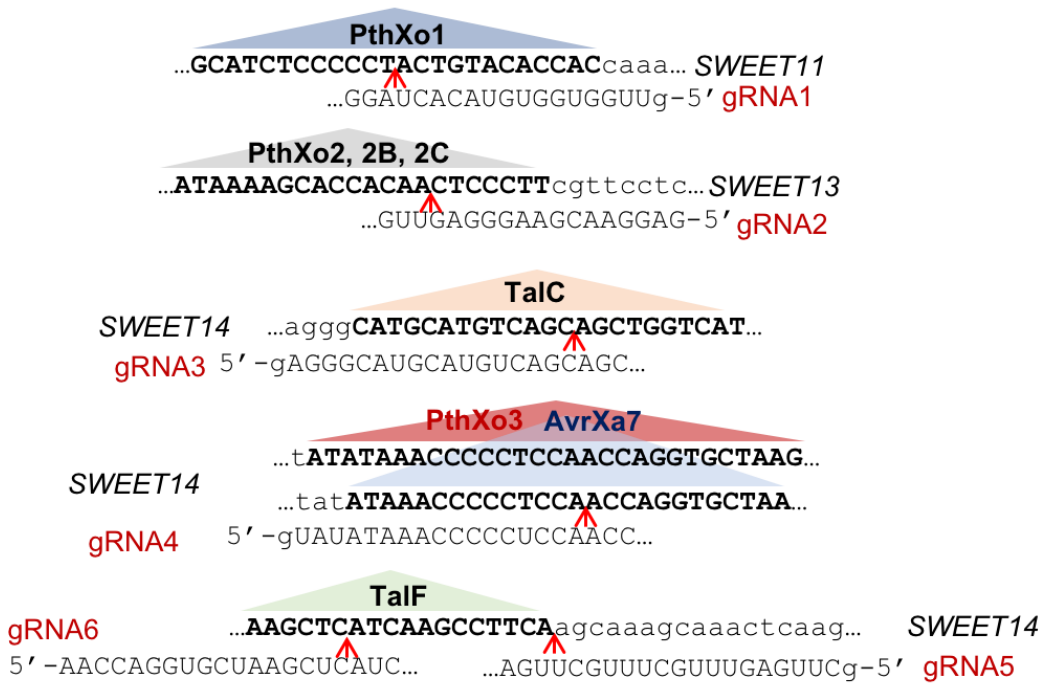

Supplementary Figure 6

Guide RNA design

Six guide RNA genes were designed and constructed to mutate five known TALe EBEs in three *SWEET* promoters. Bold letters beneath shaded TALes are their target EBEs in *SWEET* promoters. Arrows indicate Cas9/gRNA cleavages sites at their respective binding sites.

pTLN-tgRNA1

…TGGCCCGTGTCTCAAAATCTCTGATGTTACATTGCACAAGATAAAAATATATCATCATGCCTCCTCTAGAATGGGTCTCG
TGTTGAACAAAGCACCAGTGGTCTAGTGGTAGAATAGTACCCTGCCACGGTACAGACCCGGGTTCGATTCCCGGCTGGTGC
AGGGAGACGATAGATCTCAATGTCACTAGTCTCGTCTCGGTTTTAGAGCTAGAAATAGCAAGTTAAAATAAGGCTAGTCCG
TTATCAACTTGAAAAAGTGGCACCGAGTCGGTGCGGGAGCACATGAGGATCACCCATGTGCCACGAGCGACATGAGGATCA
CCCATGTCGCTCGTGTTCCCAACAGGAGACCATGCTCGAGCCACCCATGACCAAAATCCCTTAACGTGAGTTACGCGTCGT
TCCACTGAGCGTCAGACCCCGTAGAAAAGAT…

pTLN-tgRNA-2

…TGGCCCGTGTCTCAAAATCTCTGATGTTACATTGCACAAGATAAAAATATATCATCATGCCTCCTCTAGAATGGGTCTCG
AACAAAGCACCAGTGGTCTAGTGGTAGAATAGTACCCTGCCACGGTACAGACCCGGGTTCGATTCCCGGCTGGTGCAGGGA
GACGATAGATCTCAATGTCACTAGTCTCGTCTCGGTTTTAGAGCTAGAAATAGCAAGTTAAAATAAGGCTAGTCCGTTATC
AACTTGAAAAAGTGGCACCGAGTCGGTGCGGGAGCACATGAGGATCACCCATGTGCCACGAGCGACATGAGGATCACCCAT
GTCGCTCGTGTTCCCAACGGAGACCATGCTCGAGCCACCCATGACCAAAATCCCTTAACGTGAGTTACGCGTCGTTCCACT
GAGCGTCAGACCCCGTAGAAAAGAT…

pTLN-tgRNA-3

…TGGCCCGTGTCTCAAAATCTCTGATGTTACATTGCACAAGATAAAAATATATCATCATGCCTCCTCTAGAATGGGTCTCG
CAACAAAGCACCAGTGGTCTAGTGGTAGAATAGTACCCTGCCACGGTACAGACCCGGGTTCGATTCCCGGCTGGTGCAGGG
AGACGATAGATCTCAATGTCACTAGTCTCGTCTCGGTTTTAGAGCTAGAAATAGCAAGTTAAAATAAGGCTAGTCCGTTAT
CAACTTGAAAAAGTGGCACCGAGTCGGTGCGGGAGCACATGAGGATCACCCATGTGCCACGAGCGACATGAGGATCACCCA
TGTCGCTCGTGTTCCCAAGGAGACCATGCTCGAGCCACCCATGACCAAAATCCCTTAACGTGAGTTACGCGTCGTTCCACT
GAGCGTCAGACCCCGTAGAAAAGAT…

pTLN-tgRNA-4

…TGGCCCGTGTCTCAAAATCTCTGATGTTACATTGCACAAGATAAAAATATATCATCATGCCTCCTCTAGAATGGGTCTCG
CCAACAAAGCACCAGTGGTCTAGTGGTAGAATAGTACCCTGCCACGGTACAGACCCGGGTTCGATTCCCGGCTGGTGCAGG
GAGACGATAGATCTCAATGTCACTAGTCTCGTCTCGGTTTTAGAGCTAGAAATAGCAAGTTAAAATAAGGCTAGTCCGTTA
TCAACTTGAAAAAGTGGCACCGAGTCGGTGCGGGAGCACATGAGGATCACCCATGTGCCACGAGCGACATGAGGATCACCC
ATGTCGCTCGTGTTCCCAGGAGACCATGCTCGAGCCACCCATGACCAAAATCCCTTAACGTGAGTTACGCGTCGTTCCACT
GAGCGTCAGACCCCGTAGAAAAGAT…

pTLN-tgRNA-5

…TGGCCCGTGTCTCAAAATCTCTGATGTTACATTGCACAAGATAAAAATATATCATCATGCCTCCTCTAGAATGGGTCTCG
CCCAACAAAGCACCAGTGGTCTAGTGGTAGAATAGTACCCTGCCACGGTACAGACCCGGGTTCGATTCCCGGCTGGTGCAG
GGAGACGATAGATCTCAATGTCACTAGTCTCGTCTCGGTTTTAGAGCTAGAAATAGCAAGTTAAAATAAGGCTAGTCCGTT
ATCAACTTGAAAAAGTGGCACCGAGTCGGTGCGGGAGCACATGAGGATCACCCATGTGCCACGAGCGACATGAGGATCACC
CATGTCGCTCGTGTTCCCGGAGACCATGCTCGAGCCACCCATGACCAAAATCCCTTAACGTGAGTTACGCGTCGTTCCACT
GAGCGTCAGACCCCGTAGAAAAGAT…

pTLN-tgRNA-T6

…TGGCCCGTGTCTCAAAATCTCTGATGTTACATTGCACAAGATAAAAATATATCATCATGCCTCCTCTAGAATGGGTCTCG
TCCCAACAAAGCACCAGTGGTCTAGTGGTAGAATAGTACCCTGCCACGGTACAGACCCGGGTTCGATTCCCGGCTGGTGCA
GGGAGACGATAGATCTCAATGTCACTAGTCTCGTCTCGGTTTTAGAGCTAGAAATAGCAAGTTAAAATAAGGCTAGTCCGT
TATCAACTTGAAAAAGTGGCACCGAGTCGGTGCGGGAGCACATGAGGATCACCCATGTGCCACGAGCGACATGAGGATCAC
CCATGTCGCTCGTGTTCCCTTTTTTTGTCCCTTCGAAGGGCAATTCTGCAGATATCCATCACACTGGCGGCCGGTTTGGAG
ACCATGCTCGAGCCACCCATGACCAAAATCCCTTAACGTGAGTTACGCGTCGTTCCACTGAGCGTCAGACCCCGTAGAAAA
GAT…

Supplementary Figure 7

Sequence information of tRNA-gRNA constructs

Six gBlock fragments synthesized by IDT (Integrated DNA Technologies, Inc., Iowa, USA) were inserted into the vector pTLN by *Xba*I and *Xho*I (in green and box). The dots (…) are sequences in pTLN not shown. The

orientation of individual components is in order of rice glycine tRNA (in blue), gRNA scaffold (in pink) and MS2 stem-loop (in orange). Overhangs (shaded in yellow) generated by digestion of *BsmB*I (underlined) are identical in six plasmids. However, overhands (shaded in gray) generated by digestion of *Bsa*I (double underlined) are designed for assembly of the tRNA-gRNA units through Golden Gate reaction.

pENTR4-U6.1P-*ccdB*/*chl*

CAAATAATGATTTTATTTTGACTGATAGTGACCTGTTCGTTGCAACAAATTGATAAGCAATGCTTTTTTATAATGCCAACT
TTGTACAAAAAAGCAGGCTCCACCATGGGAACCAATTCAGTCGACTGGATCCAAGCTTAAGAACGAACTAAGCCGGACAAA
AAAAGGAGCACATATACAAACCGGTTTTATTCATGAATGGTCACGATGGATGATGGGGCTCAGACTTGAGCTACGAGGCCG
CAGGCGAGAGAAGCCTAGTGTGCTCTCTGCTTGTTTGGGCCGTAACGGAGGATACGGCCGACGAGCGTGTACTACCGCGCG
GGATGCCGCTGGGCGCTGCGGGGGCCGTTGGATGGGGATCGGTGGGTCGCGGGAGCGTTGAGGGGAGACAGGTTTAGTACC
ACCTCGCCTACCGAACAATGAAGAACCCACCTTATAACCCCGCGCGCTGCCGCTTGTGTTAGAGACCAGATCTTTGTAAAA
CGACGGCCAGTCTTAAGCTCGGGCCCCAAATAATGATTTTATTTTGACTGATAGTGACCTGTTCGTTGCAACACATTGATG
AGCAATGCTTTTTTATAATGCCAACTTTGTACAAAAAAGCTGAACGAGAAACGTAAAATGATATAAATATCAATATATTAA
ATTAGATTTTGCATAAAAAACAGACTACATAATACTGTAAAACACAACATATCCAGTCACTATGAATCAACTACTTAGATG
GTATTAGTGACCTGTAGTCGACCGACAGCCTTCCAAATGTTCTTCGGGTGATGCTGCCAACTTAGTCGACCGACAGCCTTC
CAAATGTTCTTCTCAAACGGAATCGTCGTATCCAGCCTACTCGCTATTGTCCTCAATGCCGTATTAAATCATAAAAAGAAA
TAAGAAAAAGAGGTGCGAGCCTCTTTTTTGTGTGACAAAATAAAAACATCTACCTATTCATATACGCTAGTGTCATAGTCC
TGAAAATCATCTGCATCAAGAACAATTTCACAACTCTTATACTTTTCTCTTACAAGTCGTTCGGCTTCATCTGGATTTTCA
GCCTCTATACTTACTAAACGTGATAAAGTTTCTGTAATTTCTACTGTATCGACCTGCAGACTGGCTGTGTATAAGGGAGCC
TGACATTTATATTCCCCAGAACATCAGGTTAATGGCGTTTTTGATGTCATTTTCGCGGTGGCTGAGATCAGCCACTTCTTC
CCCGATAACGGAGACCGGCACACTGGCCATATCGGTGGTCATCATGCGCCAGCTTTCATCCCCGATATGCACCACCGGGTA
AAGTTCACGGGAGACTTTATCTGACAGCAGACGTGCACTGGCCAGGGGGATCACCATCCGTCGCCCGGGCGTGTCAATAAT
ATCACTCTGTACATCCACAAACAGACGATAACGGCTCTCTCTTTTATAGGTGTAAACCTTAAACTGCATTTCACCAGCCCC
TGTTCTCGTCAGCAAAAGAGCCGTTCATTTCAATAAACCGGGCGACCTCAGCCATCCCTTCCTGATTTTCCGCTTTCCAGC
GTTCGGCACGCAGACGACGGGCTTCATTCTGCATGGTTGTGCTTACCAGACCGGAGATATTGACATCATATATGCCTTGAG
CAACTGATAGCTGTCGCTGTCAACTGTCACTGTAATACGCTGCTTCATAGCATACCTCTTTTTGACATACTTCGGGTATAC
ATATCAGTATATATTCTTATACCGCAAAAATCAGCGCGCAAATACGCATACTGTTATCTGGCTTTTAGTAAGCCGGATCCA
CGCGGCGTTTACGCCCCGCCCTGCCACTCATCGCAGTACTGTTGTAATTCATTAAGCATTCTGCCGACATGGAAGCCATCA
CAGACGGCATGATGAACCTGAATCGCCAGCGGCATCAGCACCTTGTCGCCTTGCGTATAATATTTGCCCATGGTGAAAACG
GGGGCGAAGAAGTTGTCCATATTGGCCACGTTTAAATCAAAACTGGTGAAACTCACCCAGGGATTGGCTGAGACGAAAAAC
ATATTCTCAATAAACCCTTTAGGGAAATAGGCCAGGTTTTCACCGTAACACGCCACATCTTGCGAATATATGTGTAGAAAC
TGCCGGAAATCGTCGTGGTATTCACTCCAGAGCGATGAAAACGTTTCAGTTTGCTCATGGAAAACGGTGTAACAAGGGTGA
ACACTATCCCATATCACCAGCTCACCGTCTTTCATTGCCATACGGAATTCCGGATGAGCATTCATCAGGCGGGCAAGAATG
TGAATAAAGGCCGGATAAAACTTGTGCTTATTTTTCTTTACGGTCTTTAAAAAGGCCGTAATATCCAGCTGAACGGTCTGG
TTATAGGTACATTGAGCAACTGACTGAAATGCCTCAAAATGTTCTTTACGATGCCATTGGGATATATCAACGGTGGTATAT
CCAGTGATTTTTTTCTCCATTTTAGCTTCCTTAGCTCCTGAAAATCTCGATAACTCAAAAAATACGCCCGGTAGTGATCTT
ATTTCATTATGGTGAAAGTTGGAACCTCTTACGTGCCGATCAACGTCTCATTTTCGCCAAAAGTTGGCCCAGGGCTTCCCG
GTATCAACAGGGACACCAGGATTTATTTATTCTGCGAAGTGATCTTCCGTCACAGGTATTTATTCGGCGCAAAGTGCGTCG
GGTGATGCTGCCAACTTAGTCGACTACAGGTCACTAATACCATCTAAGTAGTTGATTCATAGTGACTGGATATGTTGTGTT
TTACAGTATTATGTAGTCTGTTTTTTATGCAAAATCTAATTTAATATATTGATATTTATATCATTTTACGTTTCTCGTTCA
GCTTTCTTGTACAAAGTTGGCATTATAAGAAAGCATTGCTTATCAATTTGTTGCAACGAACAGGTCACTATCAGTCAAAAT
AAAATCATTATTTGCCATCCAGCTGATATCCCCTATAGTGAGTCGTACTAGTGGTCTCAGTTTAAGCTTGAATTCACTCGA
GATATCTAGACCCAGCTTTCTTGTACAAAGTTGGCATTATAAGAAAGCATTGCTTATCAATTTGTTGCAACGAACAGGTCA
CTATCAGTCAAAATAAAATCATTATTTG

Supplementary Figure 8

Sequence information of tRNA-gRNA recipient vector

The intermediate vector pENTR4-U6.1P-ccdB/chl constructed as the recipient vector for tRNA-gRNA contains two Gateway recombination sequences (in blue), rice U6 promoter (in green), two *Bsa*I (double underlined) sites. The cassettes of *ccdB* (in orange) and chl (chloramphenicol resistant) gene (in red) were constructed to facilitate the Golden Gate assembly of multiple tRNA-gRNA units.

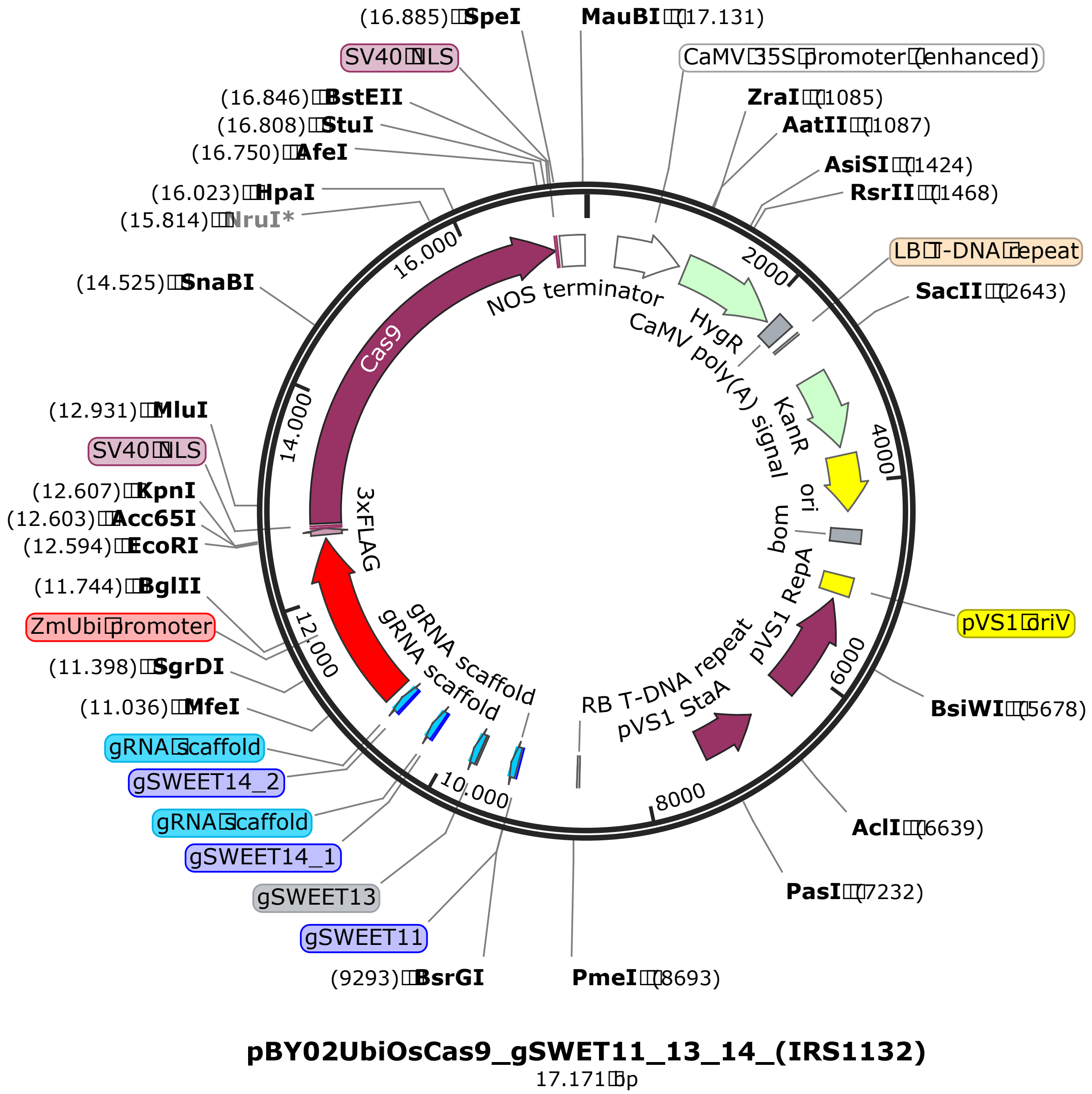

Supplementary Figure 9

Map of the CRISPR/Cas9 construct IRS1132 for simultaneous editing of four EBEs in three *SWEET* gene promoters in rice.

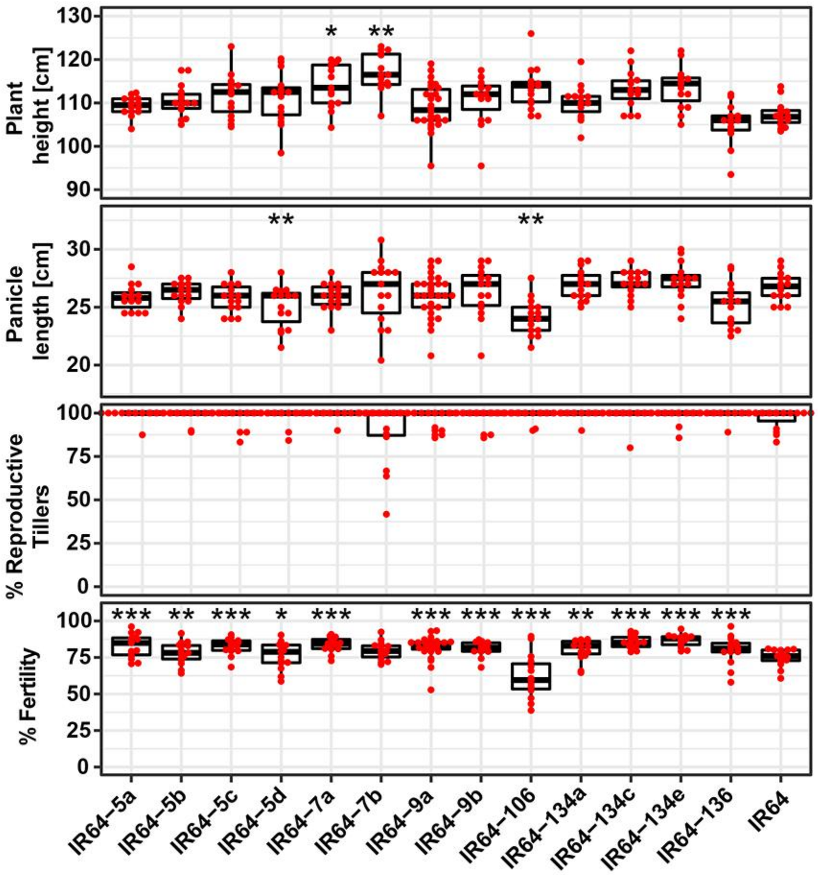

Supplementary Figure 10

Agronomic traits in selected genome-edited mega variety lines compared to the parental controls

Performance of edited IR64-IRS1132 lines in the T3 generation (plant height, panicle length; % fertility and % reproductive tillers) relative to parental controls (IR64) control (n=15). Significant differences are denoted with asterisks (two-sided Dunnett's test; $p < 0.05$ (*), $p < 0.01$ (**), $p < 0.001$ (***)). Micro-field experiments for agronomic trait assessments were conducted in a single season using Randomized Complete Block Design with three replicates. Center lines show medians; box limits indicate 25th and 75th percentiles as determined by R software.

Supplementary Table 1. List of all sequenced *Xanthomonas oryzae* pv *oryzae* (*Xoo*) strains used for analysis. Thirty-one strains were sequenced in this study, while thirty-six were previously published.

Strain	Country	Pathovar	Continent	Accession number
Xoc_BLS256	PHL	Xoc	Asia	GCA_000168315.3
BAI3	BF	Xoo	Africa	GCA_003031385.1
AXO1947	Cameroun	Xoo	Africa	GCA_001466505.1
MAI1	Mali	Xoo	Africa	GCA_003031365.1
MAI106	Mali	Xoo	Africa	GCA_002850135.1
MAI129	Mali	Xoo	Africa	GCA_002850155.1
MAI134	Mali	Xoo	Africa	GCA_002850175.1
MAI145	Mali	Xoo	Africa	GCA_002850095.1
MAI68	Mali	Xoo	Africa	GCA_002850115.1
MAI73	Mali	Xoo	Africa	GCA_002850075.1
MAI95	Mali	Xoo	Africa	GCA_002850195.1
MAI99	Mali	Xoo	Africa	GCA_002850215.1
HuN37	China	Xoo	Asia	GCA_003382775.1
IX-280	India	Xoo	Asia	GCA_003427055.1
JL25	China	Xoo	Asia	GCA_003382795.1
JL28	China	Xoo	Asia	GCA_003382815.1
JL33	China	Xoo	Asia	GCA_003382835.1
JP01	Japan	Xoo	Asia	GCA_003382855.1
KACC10331	SK	Xoo	Asia	AE013598.1
MAFF311018	Japan	Xoo	Asia	GCA_000010025.1
PXO79	PHL	Xoo	Asia	GCA_003382895.1
PXO145	PHL	Xoo	Asia	GCA_001746615.1
PXO211	PHL	Xoo	Asia	GCA_001746635.1
PXO236	PHL	Xoo	Asia	GCA_001746655.1
PXO524	PHL	Xoo	Asia	GCA_001746695.1
PXO563	PHL	Xoo	Asia	GCA_001746715.1
PXO602	PHL	Xoo	Asia	GCA_001746735.1
PXO61	PHL	Xoo	Asia	CP033187 *
PXO71	PHLs	Xoo	Asia	GCA_001746595.1
PXO83	PHL	Xoo	Asia	GCA_001518895.1
PXO86	PHL	Xoo	Asia	GCA_000948075.1
PXO99[A]	PHL	Xoo	Asia	GCA_000019585.2
ScYc-b	China	Xoo	Asia	GCA_003932055.1
SK2-3	Thailand	Xoo	Asia	GCA_003428965.1
XF89b	Taiwan	Xoo	Asia	GCA_002023005.1
XM9	Taiwan	Xoo	Asia	GCA_003522605.1
YC11	China	Xoo	Asia	GCA_003382935.1
CFBP1948	Cameroun	Xoo	Africa	CP033185 *
CFBP1949	Mali	Xoo	Africa	CP033184 *
CFBP1951	Mali	Xoo	Africa	CP033183 *
CFBP1952	Mali	Xoo	Africa	CP033182 *
CFBP7319	BF	Xoo	Africa	CP033181 *
CFBP7320	BF	Xoo	Africa	CP033186 *

CFBP7322	BF	Xoo	Africa	CP033179 *
CFBP7323	Niger	Xoo	Africa	CP033178 *
CFBP7324	Niger	Xoo	Africa	CP033177 *
CFBP7337	Mali	Xoo	Africa	CP033175 *
CFBP7340	Niger	Xoo	Africa	CP033174 *
CFBP8172	Benin	Xoo	Africa	CP033173 *
CIX1041	IC	Xoo	Africa	SIHU00000000 *
CIX2374	Senegal	Xoo	Africa	CP036377 *
CIX611	Benin	Xoo	Africa	SIHV00000000 *
CIX629	Niger	Xoo	Africa	SIHW00000000 *
Dak16	Tanzania	Xoo	Africa	CP033172 *
T19	Tanzania	Xoo	Africa	CP033171 *
Ug11	Uganda	Xoo	Africa	CP033170 *
JW11089	SK	Xoo	Asia	CP033193 *
KXO85	SK	Xoo	Asia	CP033197 *
NXO260	Nepal	Xoo	Asia	CP033192 *
PXO282	PHL	Xoo	Asia	CP01367
PXO364	PHL	Xoo	Asia	CP033191 *
PXO404	PHL	Xoo	Asia	CP033190 *
PXO421	PHL	Xoo	Asia	CP033189 *
PXO513	PHL	Xoo	Asia	CP033188 *

* Sequences generated in this manuscript, PHL, The Philippines; BF, Burkina Faso; IC, Ivory Coast; SK, South Korea

Supplementary Table 2. Disease assay on Kitaake and Kitaake-derived, TALEN-induced SWEET promoter mutants

NO.	Strain	Origin	Kitaake	*sweet11* (Line #1)	*sweet14* (Line #5)	*sweet11;sweet14* (Line #52-1)
			Lesion length (cm)			
1	PXO61	PHL	14.5±1.4	13.3±0.7	6.8±1.9	7.4±1.2
2	PXO69	PHL	15.6±3.2	3.1±0.7	8.6±2.7	2.7±1.3
3	PXO70	PHL	13.0±1.6	1.9±1.0	6.4±1.3	2.2±1.1
4	PXO71	PHL	15.3±1.7	2.4±0.9	10.0±2.5	0.9±0.6
5	PXO79	PHL	9.0±2.4	8.9±2.1	2.1±0.6	1.9±0.9
6	PXO86	PHL	12.0±2.4	13.2±3.6	2.3±1.2	2.7±0.9
7	PXO99	PHL	14.0±4.6	1.8±1.0	8.4±2.4	2.1±1.3
8	PXO101	PHL	2.6±0.4	No Test	No Test	No Test
9	PXO102	PHL	7.8±1.3	9.8±2.1	1.4±0.4	1.7±0.2
10	PXO112	PHL	13.1±2.5	12.9±2.1	4.2±0.6	2.1±0.9
11	PXO123	PHL	13.5±1.6	3.1±1.1	6.7±2.3	0.9±0.3
12	PXO124	PHL	9.7±2.0	2.5±0.8	9.1±1.8	1.1±0.4
13	PXO125	PHL	10.8±1.4	2.1±0.9	6.9±2.7	1.9±1.0
14	PXO127	PHL	6.1±1.3	2.7±1.1	6.9±1.6	2.9±1.3
15	PXO128	PHL	6.9±0.9	1.8±0.6	9.1±2.5	0.7±0.3
16	PXO129	PHL	12.9±2.9	3.3±1.0	7.8±2.5	1.2±0.9
17	PXO144	PHL	7.2±1.3	2.2±0.7	8.4±3.4	1.5±0.9
18	PXO145	PHL	11.0±1.8	8.7±1.9	4.4±1.0	1.6±1.1
19	PXO148	PHL	16.2±3.3	11.8±3.2	2.1±1.0	2.2±1.2
20	PXO163	PHL	2.1±0.3	No Test	No Test	No Test
21	PXO190	PHL	12.1±2.3	11.9±2.6	2.3±1.0	2.1±0.8
22	PXO192	PHL	16.1±2.7	13.7±3.1	3.3±1.0	1.6±0.4
23	PXO198	PHL	14.3±3.6	11.5±3.1	1.7±0.6	1.6±0.5
24	PXO200	PHL	13.5±1.6	12.8±2.5	2.1±0.8	2.4±1.2
25	PXO202	PHL	12.1±3.9	9.8±1.9	1.3±0.6	1.7±0.6
26	PXO203	PHL	7.5±1.7	8.5±1.8	3.1±1.1	1.8±0.7
27	PXO205	PHL	14.4±4.0	12.8±1.7	4.1±1.9	2.3±1.2
28	PXO208	PHL	3.2±0.6	No Test	No Test	No Test
29	PXO210	PHL	13.0±5.3	11.8±3.2	1.9±1.0	2.4±0.8
30	PXO211	PHL	5.9±1.7	7.3±1.8	2.3±0.5	0.8±0.3
31	PXO212	PHL	12.6±2.9	11.7±2.2	1.3±0.5	1.9±1.0
32	PXO220	PHL	11.8±1.7	2.7±0.8	10.4±3.3	1.3±0.7
33	PXO236	PHL	12.6±4.4	10.3±2.3	1.7±0.7	1.3±0.9
34	PXO240	PHL	13.3±3.1	11.3±2.5	1.6±0.7	1.3±0.9
35	PXO250	PHL	12.4±3.6	12.1±1.7	0.8±0.3	2.5±1.3
36	PXO280	PHL	19.9±2.5	14.7±3.2	1.2±0.4	1.8±0.9
37	PXO285	PHL	15.2±3.2	12.4±1.9	3.1±1.1	1.8±1.1
38	PXO312	PHL	14.5±2.7	10.7±3.5	3.2±1.8	1.7±1.0

39	PXO324	PHL	4.1±0.6	No Test	No Test	No Test
40	PXO338	PHL	13.3±4.7	12.3±2.3	1.7±1.2	2.2±1.2
41	PXO339	PHL	2.0±0.2	No Test	No Test	No Test
42	PXO340	PHL	16.2±4.7	12.7±3.6	2.0±1.2	1.1±0.7
43	PXO341	PHL	6.3±1.6	8.3±1.3	2.0±1.0	1.8±0.7
44	PXO342	PHL	3.0±0.9	No Test	No Test	No Test
45	PXO343	PHL	14.9±3.0	12.3±3.1	1.6±1.0	3.1±0.9
46	PXO344	PHL	2.7±0.3	No Test	No Test	No Test
47	PXO345	PHL	11.3±4.2	9.7±2.1	5.7±1.0	3.3±1.8
48	PXO346	PHL	9.4±3.6	7.9±2.4	1.5±0.7	1.8±0.5
49	PXO347	PHL	12.8±3.1	10.5±2.4	1.8±0.6	2.2±0.9
50	PXO349	PHL	1.9±0.7	No Test	No Test	No Test
51	PXO363	PHL	18.1±2.5	2.1±0.9	8.9±3.7	2.8±1.2
52	PXO364	PHL	12.6±2.0	10.3±3.1	7.6±1.9	7.3±1.7
53	PXO368	PHL	15.4±3.6	12.5±2.7	2.0±0.7	2.0±1.1
54	PXO372	PHL	16.2±3.8	13.4±2.9	3.0±1.2	1.5±0.9
55	PXO404	PHL	11.3±2.1	12.3±1.1	8.0±3.3	10.0±2.0
56	PXO421	PHL	16.1±3.9	9.3±1.9	7.4±1.6	9.6±0.8
57	PXO513	PHL	10.3±2.4	7.5±2.1	5.8±1.6	14.0±0.7
58	PXO516	PHL	9.0±1.5	8.7±2.3	4.1±0.8	2.7±1.1
59	PXO524	PHL	1.0±0.3	No Test	No Test	No Test
60	PXO563	PHL	8.2±3.0	7.8±1.1	1.8±0.6	1.9±0.8
61	PXO568	PHL	15.8±2.7	14.3±2,8	2.1±0.9	2.3±1.0
62	PXO572	PHL	12.5±3.7	13.3±1.6	2.0±1.3	2.4±1.1
63	PXO582	PHL	10.0±3.2	12.1±2.6	1.6±0.5	2.5±0.5
64	PXO589	PHL	9.9±2.3	7.5±2.1	1.4±0.6	2.7±0.9
65	PXO595	PHL	15.1±2.5	13.4±2.8	1.8±0.9	1.7±0.9
66	PXO599	PHL	18.1±3.1	14.5±2.9	4.3±1.2	3.1±0.9
67	PXO603	PHL	9.0±2.5	9.9±1.9	1.4±0.6	2.1±0.9
68	PXO604	PHL	14.5±2.8	12.5±2.4	1.4±0.9	3.1±1.2
69	PXO605	PHL	12.7±5.0	11.8±3.2	1.3±0.6	2.7±1.2
70	PXO606	PHL	13.0±2.1	10.9±2.9	1.2±0.8	4.0±1.1
71	PXO607	PHL	13.9±2.1	11.9±2.6	1.4±0.5	2.1±0.9
72	PXO608	PHL	10.3±2.7	9.7±3.1	1.9±0.4	3.2±1.1
73	PXO609	PHL	13.2±2.4	12.4±2.8	1.4±0.5	1.9±1.2
74	PXO610	PHL	13.1±2.1	12.9±2.1	2.1±1.1	1.8±1.3
75	PXO611	PHL	12.6±3.3	9.8±1.9	1.3±0.5	2.1±0.6
76	PXO612	PHL	12.4±2.1	10.5±2.9	2.7±1.2	1.7±0.7
77	C1	China	13.1±2.0	13.2±3.4	1.9±1.1	2.3±0.8
78	C3	China	10.8±2.1	9.5±2.7	1.0±0.6	1.9±0.9
79	C4	China	10.9±2.6	12.1±2.4	2.7±0.7	3.0±1.2
80	C5	China	6.6±0.2	7.3±1.5	1.9±1.0	2.4±0.8
81	C6	China	15.1±1.1	13.2±3.7	1.8±0.8	2.1±0.9

82	C7	China	8.1±2.2	7.6±1.9	2.1±0.8	2.1±0.9
83	GD1358	China	7.5±3.4	6.3±1.5	1.2±0.5	1.6±0.3
84	HLJ72	China	3.2±0.8	No Test	No Test	No Test
85	HB17	China	11.8±1.3	12.9±2.7	2.5±0.6	3.2±1.2
86	HB21	China	8.7±2.1	1.5±1.1	7.8±1.7	1.1±0.4
87	LN57	China	8.5±1.3	7.8±1.8	1.1±0.4	3.1±0.6
88	NX42	China	8.7±1.8	8.3±2.1	2.2±0.9	1.3±0.5
89	ZHE173	China	11.5±0.5	10.8±2.0	1.2±0.4	2.7±1.0
90	A3842	India	11.1±1.4	1.7±0.5	11.4±2.8	2.1±0.3
91	A3857	India	10.2±2.8	2.3±0.6	11.5±3.7	2.5±1.1
92	IX056	Indonesia	10.8±2.5	11.2±2.7	1.9±0.8	1.5±0.9
93	K202	Korea	6.1±1.1	8.1±1.2	1.5±1.0	1.9±0.9
94	KXO85	Korea	12.2±3.0	10.9±2.4	12.8±3.5	7.3±1.1
95	JW89011	Korea	15.0±2.1	11.5±2.3	9.1±1.2	9.3±3.2
96	H75373	Japan	11.9±3.0	10.4±2.3	0.6±0.2	2.2±0.8
97	JX01	Japan	12.7±3.0	11.9±2.9	1.3±0.6	1.8±0.9
98	T7174	Japan	17.3±3.0	15.5±2.9	3.4±1.0	2.0±0.9
99	NX260	Nepal	16.5±2.1	2.2±1.0	10.2±1.9	1.9±0.8
100	R-7	Thailand	8.7±3.0	7.5±2.1	1.0±0.4	1.9±1.1
101	X002	Thailand	11.0±2.4	10.8±1.9	1.7±0.3	1.3±0.5
102	Aust-2013	Australia	10.8±2.6	9.7±2.4	2.8±0.6	2.2±0.8
103	Aust-R3	Australia	12.2±2.6	10.7±2.0	1.6±0.7	2.3±0.7
104	CIAT1185	Colombia	10.3±1.7	2.3±1.3	10.3±4.0	2.8±0.9
105	AXO1947	Cameroon	12.5±2.6	10.4±1.9	10.3±3.2	13.0±2.5
Susceptibility Ratio		Susceptible	95	77	26	8
		Resistant	10	18	69	87

PHL, The Philippines. The measurements (mean ± s.e.m., n=10) of lesion length were obtained from 10 leaves (5 six-weeks old plants) 14 days after inoculation using a leaf-tip clipping inoculation method with bacterial inoculum at OD_{600}=0.5. The inoculation was repeated twice independently with similar results.

Supplementary Table 3. EBE targets of TALes from seven deviant strains

Class[a]	TALe[b]	Locus Tag[c]	Conssensus EBE	Strains[e]							Distance to TATA; Best TALVEZ Score (>6)[f]			
				PXO61	PXO421	PXO513	PXO364	PXO404	JW11089	KXO85	SWEET13_Ja[g]	SWEET13_In[h]	SWEET14	SWEET11
1	TALE3a[PXO61]	EBA26_11710	TACATAAACGCATACAAGAGAT	1	1	1	1	1	1	1			-3; 7.881	-105; 6.837
2-AvrXa7	AvrXa7JW11089		TATAAACGCCCACCCCAAGGATGACAC	0	0	0	0	0	1	1			-1; 10.381	
3	TALE2b[PXO61]	EBA26_10985	TATAAAGCAGAGCGACGATCT	1	0	0	0	0	1	1	-1; 10.492			
3	TALE2b[PXO421]		TATAAAGCAGAGCGACGAT	0	1	1	1	1	0	0	-1; 8.245			
4	TALE3b[PXO61]	EBA26_11725	TAGGAAACATGGGAATC	1	1	1	1	1	1	1			-602; 6.427	
5-PthXo2	TALE7/PthXo2BPXO61	EBA26_22640	TATAAAGCACCACAACTCCCTTC	1	1	1	1	1	0	0	-1: 11.246	-1; 6.5		
5-PthXo2	PthXo2CJW11089	EBA19_23015	TATAAAGCACCACAACTCCCTTC	0	0	0	0	0	1	1	-3: 9.637	-3: 7.115		
6	TALE4a[PXO61]	EBA26_11930	TACAAGTGACAGACCTCACC	1	0	1	1	1	0	0		-1; 7.319		
6	NA		TACAAGTGACAGACCTCACCTCTCCCCT	0	0	0	0	0	1	1		-1; 8.544		
7	TALE1a[PXO61]	EBA26_06390	TAGATTCTCTCCCT	1	1	1	1	1	1	1	194; 8.867	194; 8.867	-381; 7.038	
8-PthXo3	TALE6c/PthXo3[PXO61]	EBA26_16050	TATATAAACGCCCTCCACCGAAGGTGCCAC	1	1	1	1	1	0	0			-3; 8.558	
9	TALE6b[PXO61]	EBA26_16030	TATAATCGCCCAAGACCCTGGCAGCTAC	1	0	0	0	0	1	1				
10	TALE5a[PXO61]	EBA26_14845	TACTTTTCCCCGCCCCGT	1	1	1	1	1	0	0				-49; 7.574
10	TALE5b[PXO61]	EBA26_14865	TTACTTTCCCCGCTCACGC	1	1	1	1	1	0	0				
11	NA	EBA19_12275	TATCAGTTGCCCTCAACAT	0	0	0	0	0	1	0				
12	TALE2a[PXO61]	EBA26_10965	TAACTAGCCGGATCTCCCT	1	1	1	1	1	1	1	1; 8.089	133; 6.175		
13	TALE1b[PXO61]	EBA26_06405	TAGCTAGGGAGATCCAT	1	1	1	0	1	1	1				
14	TALE4b[PXO61]	EBA26_11945	TATATTCATCGTTTCTCCAGT	1	0	0	1	1	1	1				
14	TALE4b[PXO421]		TCATCGTTTCTCCAGT	0	1	0	0	0	0	0				
15	TALE1d[PXO61]	EBA26_06440	TCCCTCGCCCAAGCCCCACTT	1	1	1	1	1	0	0		-8; 7.401		
16	NA		TCCCTCCAAGCCCCACTT	0	0	0	0	0	0	1	191; 8.205	189; 8.205		
17	TALE1c[PXo61]	EBA26_06425	TCCGGATCATCTCTCCCAAGCCCCTGTC	1	1	1	0	1	1	1				
18	TALE6a[PXO61]	EBA26_15970	TATGTGTAGAGAGATAGACAT	1	1	1	1	1	1	1				
19	TALE4c[PXO61]	EBA26_11965	TATAGGGGGCAATCACAAACCCAC	1	1	0	1	1	0	0	-1; 6.887			
19	NA	EBA20_13110	TATAGGGGGCAATCACAAACCCAC	0	0	0	0	0	1	1				
20	TALE34c[PXO421]	EBA25_11690	TATAGGGGGCAATCCAAACCCAC	0	0	1	0	0	0	0				
21	TALE2c[PXO61]	EBA26_11005	TGCATCGCACACGCGCGGGGGGGCT	1	1	1	1	1	1	1			-363; 8.76	

a. TALe RVDs alignments from all TALes of seven variant strains were clustered and assigned to classes 1 through 22 based on similarities. Name of known TALe homolog is also included. Rows for known major TALes are highlighted in orange.
b. Name of representative TALe gene of class. NA - Not Assigned
c. NCBI Locus Tag of representative TALe gene. In process - Locus tags for PXO61 TALes will be updated.
d. Single amino acid code for 12th and 13th codons of each repeat. Asterisks represent missing amino acid based on alignment.
e. Presence or absence of TALe gene in indicated strain. 1- present (green), 0- absent
f. Distance of EBE first nucleotide from TATA box; best TALVEZ score in promoter region of indicated SWEET gene. Only scores >6 are shown.
g. SWEET13_Ja - *SWEET13* promoter from japonica subspecies (cv Kitaake)
h. SWEET13_In - *SWEET13* promoter from indica subspecies (cv IR24)

Supplementary Table 4. EBE sequences in the SWEET promoters in Kitaake and Kitaake-derived, CRISPR-induced SWEET promoter mutants. DNA sequences shaded in gray or yellow are SWEET EBEs for respective TALes. Letters in green are PAM for Cas9, TATA box is in red. Bold lower letters are insertion mutations, while dashed lines represent nucleotide deletions. Lines in bold font were subjected to disease assays (Supplementary Table S5).

SWEET11	GGTTAGATATGCATCTCCCCCTACTGTACACCACCAAAAGTGGAGGGTCTCCAACTATATAAAC (PthXo1 EBE is shaded)	Genotype
**T0 generation**		
3	GGTTAGATATGCATCTCCCCCT**a**ACTGTACACCACCAAAAGTGGAGGGTCTCCAACTATATAAAC<br>GGTTAGATATGCATCTCCCCCT**t**ACTGTACACCACCAAAAGTGGAGGGTCTCCAACTATATAAAC	+1A<br>+1T
4	GGTTAGATATGCATCTCCCCCT**a**ACTGTACACCACCAAAAGTGGAGGGTCTCCAACTATATAAAC	+1A homo
6.1	GGTTAGATATGCATCTCCCCCTACTGTACACCACCAAAAGTGGAGGG	WT
8.1	GGTTAGATATGCATCTCCCCCTACTGTACACCACCAAAAGTGGAGGG	WT
11.1	GGTTAGATATGCATCTCCCCCT**t**ACTGTACACCACCAAAAGTGGAGGG<br>GGTTAGATATGCATCTCCCCCT---GTACACCACCAAAAGTGGAGGG	+1T<br>-3bp
11.3	GGTTAGATATGCATCTCCCCCT**t**ACTGTACACCACCAAAAGTGGAGGG<br>GGTTAGATATGCATCTCCCCCT---GTACACCACCAAAAGTGGAGGG	+1T<br>-3 bp
12.2	GGTTAGATATCATCTCCCCCT**a**ACTGTACACCACCAAAAGTGGAGGG<br>GGTTAGATATCATCTCCCCCT**t**ACTGTACACCACCAAAAGTGGAGGG	+1A<br>+1T
14	GGTTAGATATGCATCTCCCCCT**a**ACTGTACACCACCAAAAGTGGAGGG	+1A homo
15.1	GGTTAGATATGCATCTCCCCCT**a**ACTGTACACCACCAAAAGTGGAGGG<br>GGTTAGATATGCATCTCCCCCT--AGTACACCACCAAAAGTGGAGGG	+1A<br>-2bp
15.2	GGTTAGATATGCATCTCCCCCT**t**ACTGTACACCACCAAAAGTGGAGGG	+1T/WT
23	GGTTAGATATGCATCTCCCCCT-CTGTACACCACCAAAAGTGGAGGG	-1A/WT
33	GGTTAGATATGCATCTCCCCCT**a**ACTGTACACCACCAAAAGTGGAGGG	+1A homo
37.1	GGTTAGATATGCATCTCCCCCT**a**ACTGTACACCACCAAAAGTGGAGGG<br>GGTTAGATATGCATCTCCCCCTA---------------AAGTGGAGGG	+1A<br>-13bp
48	GGTTAGATATGCATCTCCCCCT**a**ACTGTACACCACCAAAAGTGGAGGG	+1A homo
49	GGTTAGATATGCATCTCCCCCT**a**ACTGTACACCACCAAAAGTGGAGGG<br>GGTTAGATATGCATCTCCCCC-ACTGTACACCACCAAAAGTGGAGGG	+1A<br>-1T
51	GGTTAGATATGCATCTCCCCCT**t**ACTGTACACCACCAAAAGTGGAGGG<br>GGTTAGATATGCATCTCCCCCT**c**ACTGTACACCACCAAAAGTGGAGGG	+1T<br>+1C
58.1	GGTTAGATATCATCTCCCCCT**a**ACTGTACACCACCAAAAGTGGAGGG<br>GGTTAGATATCATCTCCCCCT**t**ACTGTACACCACCAAAAGTGGAGGG	+1A<br>+1T
62.2	GGTTAGATATCATCTCCCCCT**a**ACTGTACACCACCAAAAGTGGAGGG	+1A homo
67	GGTTAGATATCATCTCCCCCT**a**ACTGTACACCACCAAAAGTGGAGGG<br>GGTTAGATATCATCTCCCCCT------//----TGAGCCATGGCCAAGG	+1A<br>-44bp
69.2	GGTTAGATATCATCTCCCCCT**a**ACTGTACACCACCAAAAGTGGAGGG	+1A homo
**T1 generation**		
**11.1-37**	GGTTAGATATGCATCTCCCCCT---GTACACCACCAAAAGTGGAGGG	-3bp homo
**11.1-43**	GGTTAGATATGCATCTCCCCCT---GTACACCACCAAAAGTGGAGGG	-3bp homo
**11.1-45**	GGTTAGATATGCATCTCCCCCT---GTACACCACCAAAAGTGGAGGG	-3bp homo
**11.1-65**	GGTTAGATATGCATCTCCCCCT---GTACACCACCAAAAGTGGAGGG	-3bp homo
**12.2-12**	GGTTAGATATCATCTCCCCCT**t**ACTGTACACCACCAAAAGTGGAGGG	+1T homo
**12.2-50**	GGTTAGATATCATCTCCCCCT**t**ACTGTACACCACCAAAAGTGGAGGG	+1T homo
**11.1.3**	GGTTAGATATGCATCTCCCCCT---GTACACCACCAAAAGTGGAGGG	-3bp homo
SWEET13	GTCCTATATAAAGCACCACAACTCCCTTCATTCCTCTCCAAG (PthXo2B EBE is shaded)	
**T0 generation**		
3	TATATAAAGCACCACAA**a**CTCCCTTCATTCCTCTCCAAGAGTTTT<br>TATATAAAGCACCACAA-TCCCTTCATTCCTCTCCAAGAGTTTT	+1A<br>-1C
4	GTCCTATATATAAAGCACCACAA**at**CTCCCTTCATTCCTCTCCAAGAGTTTT<br>GTCCTATATATAAAGCACCACAA--------------------GAGTTTT	+2bp<br>-20bp
6.1	GTCCTATATATAAAGCACCACAACTCCCTTCATTCCTCTCCAAGAGTTTT	WT
8.1	GTCCTATATATAAAGCACCACAACTCCCTTCAATTCCTCTCCAAGAGTTTT	WT
11.1	GTCCTATATATAAAGC-------//-------CAACACATTGAACTCTTCTT<br>GTCCTATATATAAAGC----//-------------**ta**AAAATCCACAAGCATAT	-38bp<br>-740+2bp

11.3	GTCCTATATATAAAGCACCACAA**a**CTCCCTTCATTCCTCTCCAAGAGTTTT<br>GTCCTATATATAAAGC-------//-------CAACACATTGAACTCTTCTT	+1A<br>-38bp
12.2	GTCCTATATATAAAGCACCACAA**aa**CTCCCTTCATTCCTCTCCAAGAGTTTT<br>GTCCTATATATAAAGCACCACAA**a**cCTCCCTTCATTCCTCTCCAAGAGTTTT	+2AA<br>+2AC
14	GTCCTATATATAAAGCACCACAA-TCCCTTCATTCCTCTCCAAGAGTTTT	-1C/WT
15.1	GTCCTATATATAAAGCACCACAA-TCCCTTCATTCCTCTCCAAGAGTTTT	-1C/WT
15.2	GTCCTATATATAAAGCACCACAACTCCCTTCATTCCTCTCCAAGAGTTTT	WT
23	GTCCTATATATAAAGCACCACAACTCCCTTCATTCCTCTCCAAGAGTTTT	WT
33	GTCCTATATATAAAGCACCACAA**a**CTCCCTTCATTCCTCTCCAAGAGTTTT	+1A homo
37.1	GTCCTATATATAAAGCACCACAA**a**CTCCCTTCATTCCTCTCCAAGAGTTTT	+1A homo
48	GTCCTATATATAAAGCACCACAA**t**CTCCCTTCATTCCTCTCCAAGAGTTTT	+1T homo
49	GTCCTATATATAAAGCACCACAA**a**CTCCCTTCATTCCTCTCCAAGAGTTTT	+1A homo
51	GTCCTATATATAAAGCACCACAA-TCCCTTCaTTCCTCTCCAAGAGTTTT<br>GTCCTATATATAAAGCACCAC**ctacccaagagctttgctcctaccattcaagtgat**TCCCTTCaT<br>TCCTCTCCAAGAGTTTT +35	-1+35bp<br>homo
T0-58.1	GTCCTATATATAAAGCACCACAA**a**CTCCCTTCaTTCCTCTCCAAGAGTTTT<br>GTCCTATATATAAAGCACCACAA**ta**CTCCCtTCATTcCTCTcCaAgAGTTTT	+1A<br>+TA
T0-62.2	GTCCTATATATAAAGCACCACAA**t**CTCCCTTCaTTCCTCTCCAAGAGTTTT	+1T/WT
**T1 generation**		
**11.1-37**	GTCCTATATATAAAGC-------//-------CAACACATTGAACTCTTCTT<br>GTCCTATATATAAAGC----//-------------**ta**AAAATCCACAAGCATAT	-38bp<br>-740+2bp
**11.1-43**	GTCCTATATATAAAGC----//-------------**ta**AAAATCCACAAGCATAT	-740+2bp<br>homo
**T1-<br>11.1-45**	GTCCTATATATAAAGC-------//-------CAACACATTGAACTCTTCTT	-38bp<br>homo
**11.1-65**	GTCCTATATATAAAGC-------//-------CAACACATTGAACTCTTCTT	-38bp<br>homo
**12.2-12**	GTCCTATATATAAAGCACCACAA**aa**CTCCCTTCaTTCCTCTCCAAGAGTTTT	+2AA homo
**12.2-50**	GTCCTATATATAAAGCACCACAA**aa**CTCCCTTCaTTCCTCTCCAAGAGTTTT	+2AA homo
**11.1.3**	GTCCTATATATAAAGCACCACAA**aa**CTCCCTTCATTCCTCTCCAAGAGTTTT	+2AA
SWEET14	AGGGCATGCATGTCAGCAGCTGGTCATGTGTGCCTTTTCATTCCCTTCTTCCTTCCTAGCACTAT<br>ATAAACCCCCTCCAACCAGGTGCTAAGCTCATCAAGCCTTCAAGCAAAGCAAACTCAAGTAGTAG<br>CT (TalC, AvrXa7/PthXo3, TalF EBEs are shaded, respectively)	
**T0 generation**		
3	AGGGCATGCATGTCAGCAGCTGGTCATGTGTGCCTTTTCATTCCCTTCTTCCTTCCTAGCACTAT<br>ATAAACCCCCTCC-<br>ACCAGGTGCTAAGCTCATCAAGCCTTCAAGCAAAGCAAACTCAAGTAGTAGCT<br>AGGGCATGCATGTCAGCAGCTGGTCATGTGTGCCTTTTCATTCCCTTCTTCCTTCCTAGCACTAT<br>ATAAACCCCCTCCA**t**ACCAGGTGCTAAGCTCATCAAGCCTTCAAGCAAAGCAAACTCAAGTAGTA<br>GCT	WT; -1A;<br>WT<br><br>WT; +1T;<br>WT
4	AGGGCATGCATGTCAGCAGCTGGTCATGTGTGCCTTTTCATTCCCTTCTTCCTTCCTAGCACTAT<br>ATAAACCCCCTCCAACCAGGTGCTAAGCTCATCAAGCCTTCAAGCAAAGCAAACTCAAGTAGTAG<br>CT	WT; WT;<br>WT
6.1	AGGGCATGCATGTCAGC**a**AGCTGGTCATGTGTGCCTTTTCATTCCCTTCTTCCTTCCTAGCACTA<br>TATAAACCCCC----<br>ACCAGGTGCTAAGCTCATCAAGCCTTCAAGCAAAGCAAACTCAAGTAGTAGCT<br>AGGGCATGCATGTC---<br>AGCTGGTCATGTGTGCCTTTTCATTCCCTTCTTCCTTCCTAGCACTATATAAACC--------<br>---------AAGCTCATCAAGCCTTCAAGCAAAGCAAACTCAAGTAGTAGCT	+1A; -<br>4bp; WT<br><br>-3bp; -<br>16bp; -1T
8.1	AGGGCAT--------<br>GCAGCTGGTCATGTGTGCCTTTTCATTCCCTTCTTCCTTCCTAGCACTATATAAACCCCCT-<br>CAACCAGGTGCTAAGCTCATCAAGCCTTCAAGCAAAGCAAACTCAAGTAGTAGCT	-8bp<br>homo; -1C<br>homo; WT<br>homo
11.1	AGGGCATGCATGT----<br>AGCTGGTCATGTGTGCCTTTTCATTCCCTTCTTCCTTCCTAGCACTATATAAACCCCCT--<br>AACCAGGTGCTAAGCTCATCAAGCCTTCAAGCAAAGCAAACTCAAGTAGTAGCT<br>AGGGCATGCATGTCAG-----------<br>GTGTGCCTTTTCATTCCCTTCTTCCTTCCTAGCACTATATAAACCCCCTCCA**t**ACCAGGTGCTAA<br>GCTC**a**ATCAAGCCTTCAAGCAAAGCAAACTCAAGTAGTAGCT	-4bp; -<br>2bp; WT<br><br>-11bp;<br>+1T; +1A
11.3	AGGGCATGCATGT----	-4bp; -2;

	AGCTGGTCATGTGTGCCTTTTCATTCCCTTCTTCCTTCCTAGCACTATATAAACCCCCT--<br>AACCAGGTGCTAAGCTCATCAAGCCTTCAAGCAAAGCAAACTCAAGTAGTAGCT<br>AGGGCATGCATGTCAGCAGCTGGTCATGTGTGCCTTTTCATTCCCTTCTTCCTTCCTAGCACTAT<br>ATAAACCCCCTCCA**t**ACCAGGTGCTAAGCTC**a**ATCAAGCCTTCAAGCAAAGCAAACTCAAGTAGT<br>AGCT	WT<br><br>WT; +1T;<br>+1A
12.2	AGGGCATGCATGTCAGC**t**AGCTGGTCATGTGTGCCTTTTCATTCCCTTCTTCCTTCCTAGCACTA<br>TATAAACCCCCTCCA**a**ACCAGGTGCTAAGCTCATCAAGCCTTCAAGCAAAGCAAACTCAAGTAGT<br>AGCT<br>AGGGCATGCATGTCAGC---<br>---------------<br>ACCAGGTGCTAAGCTCATCAAGCCTTCAAGCAAAGCAAACTCAAGTAGTAGCT	+1T; +1A;<br>WT<br><br>-62pb; WT
14	AGGGCATGCATGTCAGCAGCTGGTCATGTGTGCCTTTTCATTCCCTTCTTCCTTCCTAGCACTAT<br>ATAAACCCCCTCCA**a**ACCAGGTGCTAAGCTCATCAAGCCTTCAAGCAAAGCAAACTCAAGTAGTA<br>GCT	WT; +1A<br>homo; WT
15.1	AGGGCATGCATGTCAGCAGCTGGTCATGTGTGCCTTTTCATTCCCTTCTTCCTTCCTAGCACTAT<br>ATAAACCCCCTCCA**a**ACCAGGTGCTAAGCTCATCAAGCCTTCAAGCAAAGCAAACTCAAGTAGTA<br>GCT<br>AGGGCATGCATGTCAGCAGCTGGTCATGTGTGCCTTTTCATTCCCTTCTTCCTTCCTAGCACTAT<br>ATAAACCCCCTCC-<br>ACCAGGTGCTAAGCTCATCAAGCCTTCAAGCAAAGCAAACTCAAGTAGTAGCT	WT; +1A;<br>WT<br><br>WT; -1A;<br>WT
15.2	AGGGCATGCATGTCAGCAGCTGGTCATGTGTGCCTTTTCATTCCCTTCTTCCTTCCTAGCACTAT<br>ATAAACCCCCTCC<br>A**t**ACCAGGTGCTAAGCTCATCAAGCCTTCAAGCAAAGCAAACTCAAGTAGTAGCT<br>AGGGCATGCATGTCAGCAGCTGGTCATGTGTGCCTTTTCATTCCCTTCTTCCTTCCTAGCACTAT<br>ATAAACCCCCTC--<br>ACCAGGTGCTAAGCTCATCAAGCCTTCAAGCAAAGCAAACTCAAGTAGTAGCT	WT; +1T;<br>WT<br><br><br>WT; -2bp;<br>WT
23	AGGGCATGCATGTCAGC**t**AGCTGGTCATGTGTGCCTTTTCATTCCCTTCTTCCTTCCTAGCACTA<br>TATAAACCCCCTCCA**a**ACCAGGTGCTAAGCTCATCAAGCCTTCAAGCAAAGCAAACTCAAGTAGT<br>AGCT<br>AGGGCATGCATGTCAGCAGCTGGTCATGTGTGCCTTTTCATTCCCTTCTTCCTTCCTAGCACTAT<br>ATAAACCCCCTCCA**t**ACCAGGTGCTAAGCTCATCAAGCCTTCAAGCAAAGCAAACTCAAGTAGTA<br>GCT	+1T; +1A;<br>WT<br><br>WT; +1T;<br>WT
33	AGGGCATGCATGTCAGCAGCTGGTCATGTGTGCCTTTTCATTCCCTTCTTCCTTCCTAGCACTAT<br>ATAAACCCCCTCCA**a**ACCAGGTGCTAAGCTCATCAAGCCTTCAAGCAAAGCAAACTCAAGTAGTA<br>GCT<br>AGGGCATGCATGTCAGC**c**AGCTGGTCATGTGTGCCTTTTCATTCCCTTCTTCCTTCCTAGCACTA<br>TATAAACCCCCTC-<br>-----------AAGCTCATCAAGCCTTCAAGCAAAGCAAACTCAAGTAGTAGCT	WT; +1A;<br>WT<br><br>+1C; -<br>12bp; WT
37.1	AGGGCATGCATGTCAGCAGCTGGTCATGTGTGCCTTTTCATTCCCTTCTTCCTTCCTAGCACTAT<br>ATAAACCCCCTCC-<br>ACCAGGTGCTAAGCTCATCAAGCCTTCAAGCAAAGCAAACTCAAGTAGTAGCT<br>AGGGCATGCATG-----<br>AGCTGGTCATGTGTGCCTTTTCATTCCCTTCTTCCTTCCTAGCACTATATAAACCCCCTCCA**t**AC<br>AGGTGCTAAGCTCATCAAGCCTTCAAGCAAAGCAAACTCAAGTAGTAGCT	-5bp; -<br>1A; WT<br><br>WT; +1T;<br>WT
48	AGGGCATGCATGTCAGCAGCTGGTCATGTGTGCCTTTTCATTCCCTTCTTCCTTCCTAGCACTAT<br>ATAAACCCCCTCC<br>-ACCAGGTGCTAAGCTCATCAAGCCTTCAAGCAAAGCAAACTCAAGTAGTAGCT<br>AGGGCATGCATG-----<br>AGCTGGTCATGTGTGCCTTTTCATTCCCTTCTTCCTTCCTAGCACTATATAAACCCCCTCCA**t**AC<br>CAGGTGCTAAGCTCATCAAGCCTTCAAGCAAAGCAAACTCAAGTAGTAGCT	WT; -1A;<br>WT<br><br>-5bp;<br>+1T; WT
49	AGGGCATGCATGTCAGCAGCTGGTCATGTGTGCCTTTTCATTCCCTTCTTCCTTCCTAGCACTAT<br>ATAAACCCCCTCC-<br>ACCAGGTGCTAAGCTCATCAAGCCTTCAAGCAAAGCAAACTCAAGTAGTAGCT<br>AGGGCATGCATGT----<br>AGCTGGTCATGTGTGCCTTTTCATTCCCTTCTTCCTTCCTAGCACTATATAAACCCCCTCCAtAC<br>CAGGTGCTAAGCTCATCAAGCCTTCAAGCAAAGCAAACTCAAGTAGTAGCT	WT; -1A;<br>WT<br><br>-4bp;<br>+1T; WT
51	AGGGCATGCATGTCAGCAGCTGGTCATGTGTGCCTTTTCATTCCCTTCTTCCTTCCTAGCACTAT<br>ATAAACCCCCTCC-<br>ACCAGGTGCTAAGCTCATCAAGCCTTCAAGCAAAGCAAACTCAAGTAGTAGCT<br>AGGGCATGCATGTCAGCAGCTGGTCATGTGTGCCTTTTCATTCCCTTCTTCCTTCCTAGCACTAT<br>ATAAACCCCCTCCA**a**ACCAGGTGCTAAGCTCATCAAGCCTTCAAGCAAAGCAAACTCAAGTAGTA<br>GCT	WT; -1A;<br>WT<br><br>WT; +1A;<br>WT

58.1	AGGGCATGCATGTCA**a**GCAGCTGGTCATGTGTGCCTTTTCATTCCCTTCTTCCTTCCTAGCACTA<br>TATAAACCCCCTCCA-<br>CCAGGTGCTAAGCTCATCAAGCCTTCAAGCAAAGCAAACTCAAGTAGTAGCT<br>AGGGCATGCATGTCAGCA--<br>TGGTCATGTGTGCCTTTTCATTCCCTTCTTCCTTCCTAGCACTATATAAACCCCCTCCAA-<br>CAGGTGCTAAGCTCATCAAGCCTTCAAGCAAAGCAAACTCAAGTAGTAGCT	+1A; -1A; WT<br><br>-2bp; -1A; WT
62.2	AGGGCATGCATGTCAGC**t**AGCTGGTCATGTGTGCCTTTTCATTCCCTTCTTCCTTCCTAGCACTA<br>TATAAACCCCCTCCA**a**ACCAGGTGCTAAGCTCATCAAGCCTTCAAGCAAAGCAAACTCAAGTAGT<br>AGCTAGGGCATGCATGT----------------<br>GTGCCTTTTCATTCCCTTCTTCCTTCCTAGCACTATATAAACCCCCTCCA-<br>CCAGGTGCTAAGCTCATCAAGCCTTCAAGCAAAGCAAACTCAAGTAGTAGCT	+1T; +1A; WT<br><br>-16bp; -1A; WT
67	AGGGCATGCATGTCAGC**t**AGCTGGTCATGTGTGCCTTTTCATTCCCTTCTTCCTTCCTAGCACTA<br>TATAAACCCCCTCCA-<br>CCAGGTGCTAAGCTCATCAAGCCTTCAAGCAAAGCAAACTCAAGTAGTAGCT<br>AGGGCATGCATG-------<br>CTGGTCATGTGTGCCTTTTCATTCCCTTCTTCCTTCCTAGCACTATATAAACCCCCTCCA**a**ACCA<br>GGTGCTAAGCTCATCAAGCCTTCAAGCAAAGCAAACTCAAGTAGTAGCT	+1T; -1A; WT<br><br>-7bp; +1A; WT
69.2	AGGGCATGCATGTCAGCAGCTGGTCATGTGTGCCTTTTCATTCCCTTCTTCCTTCCTAGCACTAT<br>ATAAACCCCCTCC<br>AACCAGGTGCTAAGCTCATCAAGCCTTCAAGCAAAGCAAACTCAAGTAGTAGCT<br>AGGGCATGCATGT----------------<br>GTGCCTTTTCATTCCCTTCTTCCTTCCTAGCACTATATAAACCCCCTCCA**a**ACCAGGTGCTAAGC<br>TCATCAAGCCTTCAAGCAAAGCAAACTCAAGTAGTAGCT	WT; WT; WT<br><br>-16bp; +1A; WT
**T1 generation**		
**11.1-37**	AGGGCATGCATGT----<br>AGCTGGTCATGTGTGCCTTTTCATTCCCTTCTTCCTTCCTAGCACTATATAAACCCCCT--<br>AACCAGGTGCTAAGCTCATCAAGCCTTCAAGCAAAGCAAACTCAAGTAGTAGCT	-4bp homo; -2bp homo; WT
**11.1-43**	AGGGCATGCATGTCAG-----------<br>GTGTGCCTTTTCATTCCCTTCTTCCTTCCTAGCACTATATAAACCCCCTCC<br>A**t**ACCAGGTGCTAAGCTC**a**ATCAAGCCTTCAAGCAAAGCAAACTCAAGTAGTAGCT	-11bp homo; +1T homo; +1A homo
**11.1-45**	AGGGCATGCATGT----<br>AGCTGGTCATGTGTGCCTTTTCATTCCCTTCTTCCTTCCTAGCACTATATAAACCCCCT--<br>AACCAGGTGCTAAGCTCATCAAGCCTTCAAGCAAAGCAAACTCAAGTAGTAGCT	-4bp homo; -2bp homo; WT
**11.1-65**	AGGGCATGCATGTCAG-----------<br>GTGTGCCTTTTCATTCCCTTCTTCCTTCCTAGCACTATATAAACCCCCTCC<br>A**t**ACCAGGTGCTAAGCTC**a**ATCAAGCCTTCAAGCAAAGCAAACTCAAGTAGTAGCT	-11bp homo; +1T homo; +1A homo
**12.2-12**	AGGGCATGCATGTCAGC---<br>--------------<br>ACCAGGTGCTAAGCTCATCAAGCCTTCAAGCAAAGCAAACTCAAGTAGTAGCT	-62bp homo; WT
**12.2-50**	AGGGCATGCATGTCAGC**t**AGCTGGTCATGTGTGCCTTTTCATTCCCTTCTTCCTTCCTAGCACTA<br>TATAAACCCCCTCC<br>A**a**ACCAGGTGCTAAGCTCATCAAGCCTTCAAGCAAAGCAAACTCAAGTAGTAGCT	+1T homo; +1A homo; WT
**11.1.3**	AGGGCATGCATGTCAG-----------<br>GTGTGCCTTTTCATTCCCTTCTTCCTTCCTAGCACTATATAAACCCCCT--<br>AACCAGGTGCTAAGCT--TCAAGCCTTCAAGCAAAGCAAACTCAAGTAGTAGCT	-11bp homo; -2T homo; -2bp homo

Supplementary Table 5. Disease reactions of genome edited *SWEET* promoter lines and the parental Kitaake line caused by representative *Xoo* strains.

**Promoter target**	**Kitaake**	**#12.2-12**	**#12.2-50**	**#11.1-65**	**#11.1-43**	**#11.1-45**	**#11.1-37**	**#11.1.3**
SWEET11 (PthXo1 EBE)	wt	+T	+T	-3	-3	-3	-3	-3
SWEET13 (PthXo2B EBE)	3As	+AA	+AA	-38	-742	-38	-38/-742	+AA
SWEET14 (TalC EBE)	wt	-62 bp	+T	-11	-11	-4	-4	-11
SWEET14 (AvrXa7/PthXo3 EBE)	wt		+A	+T	+T	-2	-2	-2
SWEET14 (TalF EBE)	wt	wt	wt	+A	+A	wt	-2/wt*	-2
ME2(*pthXo2B*)	S	**R**	**R**	**R**	**R**	**R**	**R**	**R**
ME2(*TalC*)	S	**R**	**R**	**R**	**R**	**R**	**R**	**R**
ME2(*TalF*)	S	S	S	S	S	S	S	**R**
PXO99 (PHL)	S	MS	S	**R**	S	**R**	**R**	**R**
PXO86 (PHL)	S	**R**	**R**	**R**	**R**	**R**	**R**	**R**
MAI1 (M)	S	S	S	S	S	S	S	MR
PXO61 (PHL)	S	**R**	S	S	S	S	S	**R**
PXO404 (PHL)	S	**R**	S	S	S	S	S	**R**
PXO364 (PHL)	S	**R**	S	S	S	S	S	**R**
PXO421 (PHL)	S	**R**	S	S	S	S	S	**R**
PXO513 (PHL)	S	**R**	S	S	S	S	S	**R**
KXO85 (SK)	S	**R**	**R**	**R**	**R**	**R**	**R**	**R**
JW89011 (SK)	S	**R**	**R**	**R**	**R**	**R**	**R**	**R**
AXO1947 (C)	S	MS	MS	MS	MS	MS	MS	MR

Disease phenotypes are classified into 4 categories: S, susceptible, lesion length 15-20 cm; MS, moderately susceptible, lesion length >8 cm; MR moderately resistant, lesion length between 4 and 8 cm; **R** resistant, lesion length <3 cm.
PHL: The Philippines; SK: South Korea; M: Mali; C: Cameroon.
* heterozygous line.
Blue-colored eight strains are deviant Xoo that are virulent on the TALEN-Kitaake lines as shown in Supplementary Table 2.
The disease assay was repeated twice independently with similar results.

Supplementary Table 6. DNA sequence of EBEs in IR64 and Ciherang-sub and derived CRISPR-induced SWEET promoter mutant lines.[1]

SWEET11	GGTTAGATATGCATCTCCCCCTACTGTACACCACCAAAAGTGGAGGGTCTCCAACTATATAAAC	Genotype
**T3 IR64-IRS1132**		
IR64-5a	GGTTAGATATGCATCTCCCCCT**t**ACTGTACACCACCAAAAGTGGAGGGTCTCCAACTATATAAAC	+1T homo
IR64-5b	GGTTAGATATGCATCTCCCCCT**t**ACTGTACACCACCAAAAGTGGAGGG	+1T homo
IR64-5c	GGTTAGATATGCATCTCCCCCT**a**ACTGTACACCACCAAAAGTGGAGGG	+1A homo
IR64-5d	GGTTAGATATGCATCTCCCCCT**t**ACTGTACACCACCAAAAGTGGAGGG	+1T homo
IR64-7a	GGTTAGATATGCATCTCCCCCT**a**ACTGTACACCACCAAAAGTGGAGGG	+1A homo
IR64-7b	GGTTAGATATGCATCTCCCCCT**t**ACTGTACACCACCAAAAGTGGAGGG	+1T homo
IR64-9a	GGTTAGATATGCATCTCCCCCT**a**ACTGTACACCACCAAAAGTGGAGGG	+1A homo
IR64-9b	GGTTAGATATGCATCTCCCCCT**a**ACTGTACACCACCAAAAGTGGAGGG	+1A homo
IR64-106	GGTTAGATATGCATCTCCCCCT**a**ACTGTACACCACCAAAAGTGGAGGG	+1A homo
IR64-134a	GGTTAGATATGCATCTCCCCCT**tt**ACTGTACACCACCAAAAGTGGAGG	+2TT homo
IR64-134e	GGTTAGATATGCATCTCCCCCT**tt**ACTGTACACCACCAAAAGTGGAGG	+2TT homo
IR64-134c	GGTTAGATATGCATCTCCCCCT**tt**ACTGTACACCACCAAAAGTGGAGG	+2TT homo
IR64-136	GGTTAGATATGCATCTCCCCCT**t**ACTGTACACCACCAAAAGTGGAGGG	+1T homo
**T2 Ciherang-Sub1-IRS1132**		
CS-1a	GGTTAGATATGCATCTCCCCCT---GTACACCACCAAAAGTGGAGGG	-3bp homo
CS-1b	GGTTAGATATGCATCTCCCCCT**a**ACTGTACACCACCAAAAGTGGAGGG	+1A homo
CS-1c	GGTTAGATATGCATCTCCCCCT**a**ACTGTACACCACCAAAAGTGGAGGG	+1A homo
CS-1d	GGTTAGATATGCATCTCCCCCT**a**ACTGTACACCACCAAAAGTGGAGGG<br>GGTTAGATATGCATCTCCCCCT---GTACACCACCAAAAGTGGAGGG	Bi:+1A//-3
CS-1e	identical to CS-1d	Bi:+1A//-3
CS-1f	GGTTAGATATGCATCTCCCCCT**a**ACTGTACACCACCAAAAGTGGAGGG	+1A homo
CS-1g	identical to CS-1d	Bi:+1A//-3
CS-1h	GGTTAGATATGCATCTCCCCCT---GTACACCACCAAAAGTGGAGGG	-3 homo
CS-3a	GGTTAGATATGCATCTCCCCCT**a**ACTGTACACCACCAAAAGTGGAGGG<br>GGTTAGATATGCATCTCCCCC---------CCACCAAAAGTGGAGGG	Bi:+1A//-9
CS-3b	identical to CS-3a	Bi:+1A//-9
CS-3c	identical to CS-3a	Bi:+1A//-9
CS-4a	GGTTA----------------------- CACCACCAAAAGTGGAGGG	-23bp homo
CS-6a	GGTTAGATATGCATCTCCCCCT**a**ACTGTACACCACCAAAAGTGGAGGG	+1A homo
CS-6b	GGTTAGATATGCATCTCCCCCT**a**ACTGTACACCACCAAAAGTGGAGGG	+1A homo
CS-6c	GGTTAGATATGCATCTCCCCCT**a**ACTGTACACCACCAAAAGTGGAGGG	+1A homo
CS-6d	GGTTAGATATGCATCTCCCCCT**a**ACTGTACACCACCAAAAGTGGAGGG	+1A homo
CS-6e	GGTTAGATATGCATCTCCCCCT**a**ACTGTACACCACCAAAAGTGGAGGG	+1A homo
CS-6f	GGTTAGATATGCATCTCCCCCT**a**ACTGTACACCACCAAAAGTGGAGGG	+1A homo
CS-6g	GGTTAGATATGCATCTCCCCCT**a**ACTGTACACCACCAAAAGTGGAGGG	+1A homo
SWEET13	GTCCTATATAAAAGCACCACAACTCCCTTCGTTCCTCTCCAAGAGTTTT	
**T3 IR64-IRS1132**		
IR64-5a	GTCCTATATAAAAGCACCACAA**aa**CTCCCTTCGTTCCTCTCCAAGAGTTTT	+2AA homo
IR64-5b	GTCCTATATAAAAGCACCACAA**aa**CTCCCTTCGTTCCTCTCCAAGAGTTTT	+2AA homo
IR64-5c	GTCCTATATAAAAGCACCACAA**aa**CTCCCTTCGTTCCTCTCCAAGAGTTTT	+2AA homo
IR64-5d	GTCCTATATAAAAGCACCACAA**aa**CTCCCTTCGTTCCTCTCCAAGAGTTTT	+2AA homo
IR64-7a	GTCCTATATATAAAGCACCACAA-TCCCTTCATTCCTCTCCAAGAGTTTT	-1bp homo
IR64-7b	GTCCTATATAAAAGCACCACAA**aa**CTCCCTTCGTTCCTCTCCAAGAGTTTT	+2AA homo
IR64-9a	GTCCTATATAAAAGCACCACAA**a**CTCCCTTCATTCCTCTCCAAGAGTTTT	+1A homo
IR64-9b	GTCCTATATAAAAGCACCACAA**a**CTCCCTTCATTCCTCTCCAAGAGTTTT	+1A homo
IR64-106	GTCCTATATAAAAGCACCACAA**aa**CTCCCTTCGTTCCTCTCCAAGAGTTTT	+2AA homo
IR64-134a	GTCCTATATAAAAGCACC-------CCTTCGTTCCTCTCCAAGAGTTTT	-7bp homo
IR64-134e	GTCCTATATAAAAGCACC-------CCTTCGTTCCTCTCCAAGAGTTTT	-7bp homo
IR64-134c	GTCCTATATAAAAGC--------TCCCTTCGTTCCTCTCCAAGAGTTTT	-8bp homo
IR64-136	GTCCTATATAAAAGCACCACAA**t**CTCCCTTCATTCCTCTCCAAGAGTTTT	+1T homo
**T2 Ciherang-Sub1-IRS1132**		

CS-1a	GTCCTATATATATAAAGCACCACAA-TCCCTTCATTCCTCTCCAAGAGTTTT	-1bp homo
CS-1b	GTCCTATATAAAAGCACCACAA**a**CTCCCTTCATTCCTCTCCAAGAGTTTT	+1A homo
CS-1c	GTCCTATATAAAAGCACCACAA**aa**CTCCCTTCGTTCCTCTCCAAGAGTTTT	+2AA homo
CS-1d	GTCCTATATATATAAAGCACCACAA-TCCCTTCATTCCTCTCCAAGAGTTTT	-1bp homo
CS-1e	GTCCTATATAAAAGCACCACAA**a**CTCCCTTCATTCCTCTCCAAGAGTTTT<br>GTCCTATATATATAAAGCACCACAA-TCCCTTCATTCCTCTCCAAGAGTTTT	Bi:+1A//-1bp
CS-1f	GTCCTATATAAAAGCACCACAA**a**CTCCCTTCGTTCCTCTCCAAGAGTTTT	+1A homo
CS-1g	GTCCTATATAAAAGCACCACAA**a**CTCCCTTCGTTCCTCTCCAAGAGTTTT	+1A homo
CS-1h	GTCCTATATAAAAGCACCACAA**a**CTCCCTTCGTTCCTCTCCAAGAGTTTT<br>GTCCTATATATATAAAGCACCACAA-TCCCTTCATTCCTCTCCAAGAGTTTT	Bi:+1A//-1bp
CS-3a	GTCCTATATATATAAAGCACCACAA-TCCCTTCATTCCTCTCCAAGAGTTTT	-1bp homo
CS-3b	GTCCTATATATATAAAGCACCACAA-TCCCTTCATTCCTCTCCAAGAGTTTT	-1bp homo
CS-3c	GTCCTATATATATAAAGCACCACAA-TCCCTTCATTCCTCTCCAAGAGTTTT	-1bp homo
CS-4a	GTCCTATATAAAAGCACCACAA**a**CTCCCTTCATTCCTCTCCAAGAGTTTT	+1,A homo
CS-6a	GTCCTATATATATAAAGCACCACAA--CCCTTCATTCCTCTCCAAGAGTTTT	-2bp homo
CS-6b	GTCCTATATATATAAAGCACCACA---CCCTTCATTCCTCTCCAAGAGTTTT	-3bp homo
CS-6c	GTCCTATATATATAAAGCACCACAA--CCCTTCATTCCTCTCCAAGAGTTTT	-2bp homo
CS-6d	GTCCTATATATATAAAGCACCACAA--CCCTTCATTCCTCTCCAAGAGTTTT	-2bp homo
CS-6e	GTCCTATATATATAAAGCACCACAA--CCCTTCATTCCTCTCCAAGAGTTTT	-2bp homo
CS-6f	GTCCTATATATATAAAGCACCACAA--CCCTTCATTCCTCTCCAAGAGTTTT<br>GTCCTATATATATAAAGCACCACA---CCCTTCATTCCTCTCCAAGAGTTTT	Bi:-2//-3bp
CS-6g	identical to CS-6f	Bi:-2//-3bp
SWEET14	AGGGCATGCATGTCAGCAGC**TGG**TCATGTGTGCCTTTTCATTCCCTTCTTCCTTCCTA GCACTATATAAACCCCCTCCAACC**AGG**TGCT**AAG**CTCATCAAG**CCT**TCAAGCAAAGCA AACTCAAGTAGTAGCT	WT; WT; WT
**T3 IR64-IRS1132**		
IR64-5a	AGGGCATGCATGTCAGC-<br>GCTGGTCATGTGTGCCTTTTCATTCCCTTCTTCCTTCCTAGCACTATATAAACCCCCT<br>CCA**c**ACCAGGTGCTAAGCTCATCAAGCCTTCAAGCAAAGCAAACTCAAGTAGTAGCT<br>AGGGCATGCATGTC---<br>AGCTGGTCATGTGTGCCTTTTCATTCCCTTCTTCCTTCCTAGCACTATATAAACCCCC<br>------CAGGTGCTAAGCTCATCAAGCCTTCAAGCAAAGCAAACTCAAGTAGTAGCT	Bi:-1bp; +1C; WT//-3bp; -7bp; WT
IR64-5b	AGGGCATGCATGTCAGC-<br>GCTGGTCATGTGTGCCTTTTCATTCCCTTCTTCCTTCCTAGCACTATATAAACCCCCT<br>CCA**c**ACCAGGTGCTAAGCTCATCAAGCCTTCAAGCAAAGCAAACTCAAGTAGTAGCT	-1bp; +1C; WT homo
IR64-5c	AGGGCATGCATGTCAGCA--<br>----------------------<br>ACCAGGTGCTAAGCTCATCAAGCCTTCAAGCAAAGCAAACTCAAGTAGTAGCT	-61bp homo
IR64-5d	AGGGCATGCATGTCAGC-<br>GCTGGTCATGTGTGCCTTTTCATTCCCTTCTTCCTTCCTAGCACTATATAAACCCCCT<br>CCA**c**ACCAGGTGCTAAGCTCATCAAGCCTTCAAGCAAAGCAAACTCAAGTAGTAGCT	-1bp; +1C; WT homo
IR64-7a	AGGGCATGCATGTCAGC-<br>GCTGGTCATGTGTGCCTTTTCATTCCCTTCTTCCTTCCTAGCACTATATAAACCCCCT<br>CCA**a**ACCAGGTGCTAAGCTCATCAAGCCTTCAAGCAAAGCAAACTCAAGTAGTAGCT<br>AGGGCATGCATGTCAGCA--<br>----------------------<br>ACCAGGTGCTAAGCTCATCAAGCCTTCAAGCAAAGCAAACTCAAGTAGTAGCT	Bi:<br>-1bp; +1C; WT//-61bp; WT
IR64-7b	AGGGCATGCATGTCAGC-<br>GCTGGTCATGTGTGCCTTTTCATTCCCTTCTTCCTTCCTAGCACTATATAAACCCCCT<br>CCA**c**ACCAGGTGCTAAGCTCATCAAGCCTTCAAGCAAAGCAAACTCAAGTAGTAGCT	-1bp; +1C; WT homo
IR64-9a	AGGGCATGCATGTCAGC----------------------------CCTT**a**TTC-------<br>-----------<br>A**ag**CC**t**TC**a**AACCAGGTGCTAAGCTCATCAAGCCTTCAAGCAAAGCAAACTCAAGTAG TAGCT	-44(-58+14)bp;+1A; WT homo
IR64-9b	Identical to IR64-9a	-44(-58+14)bp;+1A; WT homo
IR64-106	AGGGCATGCATGTCAGCA--<br>-----------------------<br>CCAGGTGCTAAGCTCATCAAGCCTTCAAGCAAAGCAAACTCAAGTAGTAGCT	-62bp; WT homo

IR64-134a	AGGGCATGCATG------<br>GCTGGTCATGTGTGCCTTTTCATTCCCTTCTTCCTTCCTAGCACTATATAAACC----<br>---ACCAGGTGCTAAGCTCATCAAGCCTTCAAGCAAAGCAAACTCAAGTAGTAGCT	-6bp; -7bp; WT homo
IR64-134e	AGGGCATGCATG------<br>GCTGGTCATGTGTGCCTTTTCATTCCCTTCTTCCTTCCTAGCACTATATAAACC----<br>---ACCAGGTGCTAAGCTCATCAAGCCTTCAAGCAAAGCAAACTCAAGTAGTAGCT	-6bp; -7bp; WT homo
IR64-134c	AGGGCATGCATG------<br>GCTGGTCATGTGTGCCTTTTCATTCCCTTCTTCCTTCCTAGCACTATATAAACC----<br>---ACCAGGTGCTAAGCTCATCAAGCCTTCAAGCAAAGCAAACTCAAGTAGTAGCT	-6bp; -7bp; WT homo
IR64-136	AGGGCATGCATGTCAGCA--<br>----------------------<br>CCAGGTGCTAAGCTCATCAAGCCTTCAAGCAAAGCAAACTCAAGTAGTAGCT	-62bp; WT homo
**T2 Ciherang-Sub1-IRS1132**		
CS-1a	AGGGCATGCATGTCAGC---<br>TGGTCATGTGTGCCTTTTCATTCCCTTCTTCCTTCCTAGCACTATATAAACCCCCTCC<br>AA**a**CCAGGTGCTAAGCTCATCAAGCCTTCAAGCAAAGCAAACTCAAGTAGTAGCT	-3bp; +1A; WT homo
CS-1b	AGGGCATGCATG------<br>**a**CTGGTCATGTGTGCCTTTTCATTCCCTTCTTCCTTCCTAGCACTATATAAACCCCCT<br>CCAA**a**CCAGGTGCTAAGCTCATCAAGCCTTCAAGCAAAGCAAACTCAAGTAGTAGCT	-6bp, G>A; +1A; WT homo
CS-1c	AGGGCATGCATGTCAGC---<br>TGGTCATGTGTGCCTTTTCATTCCCTTCTTCCTTCCTAGCACTATATAAACCCCCTCC<br>AA**a**CCAGGTGCTAAGCTCATCAAGCCTTCAAGCAAAGCAAACTCAAGTAGTAGCT	-3bp; +1A; WT homo
CS-1d	AGGGCATGCATGTCAGC---<br>TGGTCATGTGTGCCTTTTCATTCCCTTCTTCCTTCCTAGCACTATATAAACCCCCTCC<br>AA**a**CCAGGTGCTAAGCTCATCAAGCCTTCAAGCAAAGCAAACTCAAGTAGTAGCT	-3bp; +1A; WT homo
CS-1e	AGGGCATGCATG------<br>**a**CTGGTCATGTGTGCCTTTTCATTCCCTTCTTCCTTCCTAGCACTATATAAACCCCCT<br>CCAA**a**CCAGGTGCTAAGCTCATCAAGCCTTCAAGCAAAGCAAACTCAAGTAGTAGCT	-6bp, G>A; +1A; WT homo
CS-1f	AGGGCATGCATG------<br>**a**CTGGTCATGTGTGCCTTTTCATTCCCTTCTTCCTTCCTAGCACTATATAAACCCCCT<br>CCAA**a**CCAGGTGCTAAGCTCATCAAGCCTTCAAGCAAAGCAAACTCAAGTAGTAGCT	-6bp, G>A; +1A; WT homo
CS-1g	AGGGCATGCATG------<br>**a**CTGGTCATGTGTGCCTTTTCATTCCCTTCTTCCTTCCTAGCACTATATAAACCCCCT<br>CCAA**a**CCAGGTGCTAAGCTCATCAAGCCTTCAAGCAAAGCAAACTCAAGTAGTAGCT	-6bp, G>A; +1A; WT homo
CS-1h	AGGGCATGCATGTCAGC---<br>TGGTCATGTGTGCCTTTTCATTCCCTTCTTCCTTCCTAGCACTATATAAACCCCCTCC<br>AA**a**CCAGGTGCTAAGCTCATCAAGCCTTCAAGCAAAGCAAACTCAAGTAGTAGCT	-3bp; +1A; WT homo
CS-3a	AGGGCATGCATGTCA-----------<br>TGTGTGCCTTTTCATTCCCTTCTTCCTTCCTAGCACTATATAAA--------------<br>-----<br>-AGCTCATCAAGCCTTCAAGCAAAGCAAACTCAAGTAGTAGCT	-11bp; -20bp; WT homo
CS-3b	AGGGCATGCATGTCA-----------<br>TGTGTGCCTTTTCATTCCCTTCTTCCTTCCTAGCACTATATAAA--------------<br>-----<br>-AGCTCATCAAGCCTTCAAGCAAAGCAAACTCAAGTAGTAGCT	-11bp; -20bp; WT homo
CS-3c	AGGGCATGCATGTCA-----------<br>TGTGTGCCTTTTCATTCCCTTCTTCCTTCCTAGCACTATATAAA--------------<br>-----<br>-AGCTCATCAAGCCTTCAAGCAAAGCAAACTCAAGTAGTAGCT	-11bp; -20bp; WT homo
CS-4a	AGGGCATGCATGTCAGCA--<br>----------------------<br>CCAGGTGCTAAGCTCATCAAGCCTTCAAGCAAAGCAAACTCAAGTAGTAGCT	-62bp; WT homo
CS-6a	AGGGCATGCATGTCAGCA--<br>---------------------<br>ACCAGGTGCTAAGCTCATCAAGCCTTCAAGCAAAGCAAACTCAAGTAGTAGCT	-61bp; WT homo
CS-6b	AGGGCATGCATGTCAGCA--<br>---------------------<br>ACCAGGTGCTAAGCTCATCAAGCCTTCAAGCAAAGCAAACTCAAGTAGTAGCT	-61bp; WT homo
CS-6c	AGGGCATGCATGTCAGCA--<br>---------------------<br>ACCAGGTGCTAAGCTCATCAAGCCTTCAAGCAAAGCAAACTCAAGTAGTAGCT	-61bp; WT homo
CS-6d	AGGGCATGCATGTCAGCA--	-61bp; WT

	---------------------<br>ACCAGGTGCTAAGCTCATCAAGCCTTCAAGCAAAGCAAACTCAAGTAGTAGCT	homo
CS-6e	AGGGCATGCATGTCAGCA--<br>---------------------<br>ACCAGGTGCTAAGCTCATCAAGCCTTCAAGCAAAGCAAACTCAAGTAGTAGCT	-61bp; WT homo
CS-6f	AGGGCATGCATG------<br>GCTGGTCATGTGTGCCTTTTCATTCCCTTCTTCCTTCCTAGCACTATATAAACCCCCT<br>CCA-CCAGGTGCTAAGCTCATCAAGCCTTCAAGCAAAGCAAACTCAAGTAGTAGCT	-6bp; -1bp; WT homo
CS-6g	AGGGCATGCATGTCAGCA--<br>---------------------<br>ACCAGGTGCTAAGCTCATCAAGCCTTCAAGCAAAGCAAACTCAAGTAGTAGCT	-61bp; WT homo

1 DNA sequences shaded in gray or yellow are SWEET EBEs for respective TALes. Letters in green are PAM for Cas9, TATA box is in red. Bold lower letters are insertion mutations, while dashed lines represent nucleotide deletions.

Supplementary Table 7. Susceptibility tests of EBE-edited variants in IR64 and Ciherang-Sub1 (also see Fig. 5) and nucleotide changes in EBEs of *SWEET* promoters.

		Xoo pathogenicity[1]			*SWEET11*	*SWEET13*	*SWEET14*	
**Variant line**		**PXO99**	**PXO339**	**PXO86**	**PthXo1**	**PthXo2**	**TalC**	**AvrXa7 PthXo3**
**IR64**	IR64-5a	MS	R	R	+1, T	+2, AA	-1, A<br>-3 bp	+1, C<br>-7 bp
	IR64-5b	MS	R	R	+1, T	+2, AA	-1, A	+1, C
	IR64-5c	MR	R	R	+1, A	+2, AA	-61 bp	
	IR64-5d	R	R	R	+1, T	+2, AA	-1, A	+1, C
	IR64-7a	MS	R	R	+1, A	-1, C	-1, A<br>-61 bp	+1, C
	IR64-7b	MR	R	R	+1, T	+2, AA	-1, A	+1, C
	IR64-9a	MS	R	R	+1, A	+1, A	-44 bp	+1, A
	IR64-9b	MS	R	R	+1, A	+1, A	-44 bp	+1, A
	IR64-106	MR	R	R	+1, A	+2, AA	-62 bp	
	IR64-134a	R	R	R	+2, TT	-7bp	-6 bp	-7 bp
	IR64-134e	R	R	R	+2, TT	-7bp	-6 bp	-7 bp
	IR64-134c	R	R	R	+2, TT	-8bp	-6 bp	-7 bp
	IR64-136	R	R	R	+1, T	+1, T	-62 bp	
**Ciherang-Sub1**	CS-1a	MR	R	R	-3 bp	-1, C	-3 bp	+1, A
	CS-1b	R	R	R	+1, A	+1, A	-6 bp	+1, A
	CS-1c	MR	R	R	+1, A	+2, AA	-3 bp	+1, A
	CS-1d	MR	R	R	+1, A<br>-3 bp	-1, C	-3 bp	+1, A
	CS-1e	MR	R	R	+1, A<br>-3 bp	+1, A<br>-1, C	-6 bp	+1, A
	CS-1f	MS	R	R	+1, A	+1, A	-6 bp	+1, A
	CS-1g	MR	R	R	+1, A<br>-3 bp	+1, A	-6 bp	+1, A
	CS-1h	MR	R	R	-3 bp	+1, A<br>-1, C	-3 bp	+1, A
	CS-3a	MR	MS	R	+1, A<br>-9 bp	-1, C	-11 bp	-20 bp
	CS-3b	R	R	R	+1, A<br>-9 bp	-1, C	-11 bp	-20 bp
	CS-4a	R	R	R	-23bp	+1, A	-62 bp	
	CS-6a	R	MR	MS	+1, A	-2 bp	-61 bp	
	CS-6b	MS	R	MS	+1, A	-3 bp	-61 bp	
	CS-6c	MR	R	R	+1, A	-2 bp	-61 bp	
	CS-6d	MR	R	R	+1, A	-2 bp	-61 bp	
	CS-6e	MR	R	R	+1, A	-2 bp	-61 bp	
	CS-6f	MR	R	R	+1, A	-2 bp<br>-3 bp	-6 bp	-1, A

	CS-6g	MR	R	R	+1, A	-2 bp<br>-3 bp	-61 bp

[1] R: resistant, MR: moderately resistant, S: susceptible. The disease assay was repeated three times independently with similar results.
>: SNP, inserted nucleotides are indicated after the comma, deleted nucleotides are not indicated.

Supplementary Table 8. Prediction of on- and potential off-target sites in various rice varieties for guide RNAs used in this study.

<table>
<tr><th colspan="2">SWEET11</th><th>Alignment</th></tr>
<tr><td colspan="2">gRNA1 (5’-3’)</td><td>GUUGGUGGUGUACACUAGG</td></tr>
<tr><td colspan="2">Genomic target sequence</td><td>CCCCCTACTGTACACCACCAA</td></tr>
<tr><td rowspan="2">Nipponbare</td><td>On-target</td><td>Query 1 CCCCCTACTGTACACCACCAA 21
|||||||||||||||||||||
Sbjct 26728867 CCCCCTACTGTACACCACCAA 26728847</td></tr>
<tr><td>Potential off-target</td><td>Query 2 CCCCTACTGTACAC 15
||||||||||||||
Sbjct 20139965 CCCCTACTGTACAC 20139978</td></tr>
<tr><td rowspan="2">Shuhui498</td><td>On-target</td><td>Query 1 CCCCCTACTGTACACCACCAA 21
|||||||||||||||||||||
Sbjct 28035825 CCCCCTACTGTACACCACCAA 28035805</td></tr>
<tr><td>Potential off-target</td><td>Query 2 CCCCTACTGTACACCAC 18
|||||||||||| ||||
Sbjct 20525144 CCCCTACTGTACCCCAC 20525128</td></tr>
<tr><td rowspan="2">RP Bio-226</td><td>On-target</td><td>Query 1 CCCCCTACTGTACACCACCAA 21
|||||||||||||||||||||
Sbjct 26242990 CCCCCTACTGTACACCACCAA 26242970</td></tr>
<tr><td>Potential off-target</td><td>Query 6 TACTGTACACCACCAA 21
|||||||| |||||||
Sbjct 4640517 TACTGTACTCCACCAA 4640532</td></tr>
<tr><td>IR64</td><td>On-target</td><td>Query 1 CCCCCTACTGTACACCACCAA 21
|||||||||||||||||||||
Sbjct 1347 CCCCCTACTGTACACCACCAA 1367</td></tr>
</table>

<table>
<tr><th colspan="2">SWEET13</th><th></th></tr>
<tr><td colspan="2">gRNA2 (5’-3’)</td><td>GAGGAACGAAGGAGUUG</td></tr>
<tr><td colspan="2">Genomic target sequence</td><td>CCACAACTCCCTTCGTTCCTC</td></tr>
<tr><td rowspan="2">Nipponbare</td><td>On-target</td><td>Query 1 CCACAACTCCCTTCGTTCCTC 21
|||||||||||||| ||||||
Sbjct 17305350 CCACAACTCCCTTCATTCCTC 17305330</td></tr>
<tr><td>Potential off-target</td><td>Query 3 ACAACTCCCTTCGTT 17
|||||||||||||||
Sbjct 15466732 ACAACTCCCTTCGTT 15466746</td></tr>
<tr><td>Shuhui498</td><td>On-target</td><td>Query 1 CCACAACTCCCTTCGTTCCTC 21
||||||| |||||| ||||||
Sbjct 16795195 CCACAACACCCTTCATTCCTC 16795175</td></tr>
</table>

<table>
<tr><th colspan="2">SWEET14</th><th></th></tr>
<tr><td colspan="2">gRNA3 (5’-3’)</td><td>GAGGGCAUGCAUGUCAGCAGC</td></tr>
<tr><td colspan="2">Genomic target sequence</td><td>AGGGCATGCATGTCAGCAGCTGG</td></tr>
<tr><td rowspan="2">Nipponbare</td><td>On-target</td><td>Query 1 AGGGCATGCATGTCAGCAGCTGG 23
|||||||||||||||||||||||
Sbjct 18174573 AGGGCATGCATGTCAGCAGCTGG 18174551</td></tr>
<tr><td>Potential off-target</td><td>Query 3 GGCATGCATGTCAGCA 18
||||||||||||||||
Sbjct 1669705 GGCATGCATGTCAGCA 1669690</td></tr>
<tr><td rowspan="2">RP Bio-226</td><td>On-target</td><td>Query 1 AGGGCATGCATGTCAGCAGCTGG 23
|||||||||||||||||||||||
Sbjct 14010709 AGGGCATGCATGTCAGCAGCTGG 14010687</td></tr>
<tr><td>Potential off-target</td><td>Query 3 GGCATGCATGTCAGCA 18
||||||||||||||||
Sbjct 1240414 GGCATGCATGTCAGCA 1240399</td></tr>
<tr><td colspan="2">gRNA4 (5’-3’)</td><td>GUAUAUAACCCCCUCCAACC</td></tr>
<tr><td colspan="2">Genomic target sequence</td><td>TATATAAACCCCCTCCAACCAGG</td></tr>
<tr><td>Nipponbare</td><td>On-target</td><td>Query 1 TATATAAACCCCCTCCAACCAGG 23
|||||||||||||||||||||||</td></tr>
</table>

<table>
<tr><td></td><td></td><td>Sbjct 18174511 TATATAAACCCCCTCCAACCAGG 18174489</td></tr>
<tr><td></td><td>Potential off-target</td><td>Query 2 ATATAAACCCCCTC 15
||||||||||||||
Sbjct 16124639 ATATAAACCCCCTC 16124626</td></tr>
<tr><td rowspan="2">Shuhui498</td><td>On-target</td><td>Query 1 TATATAAACCCCCTCCAACCAGG 23
|||||||||||||||||||||||
Sbjct 20518383 TATATAAACCCCCTCCAACCAGG 20518361</td></tr>
<tr><td>Potential off-target</td><td>Query 7 AACCCCCTCCAACCAGG 23
||||||||| |||||||
Sbjct 4209876 AACCCCCTCTAACCAGG 4209860</td></tr>
<tr><td rowspan="2">RP Bio-226</td><td>On-target</td><td>Query 1 TATATAAACCCCCTCCAACCAGG 23
|||||||||||||||||||||||
Sbjct 14010647 TATATAAACCCCCTCCAACCAGG 14010625</td></tr>
<tr><td>Potential off-target</td><td>Query 7 AACCCCCTCCAACCAGG 23
||||||||| |||||||
Sbjct 3810462 AACCCCCTCTAACCAGG 3810446</td></tr>
<tr><td colspan="2">gRNA5 (5'-3')</td><td>GCUUGAGUUUGCUUUGCUUGA</td></tr>
<tr><td colspan="2">Genomic target sequence</td><td>CCTTCAAGCAAAGCAAACTCAAG</td></tr>
<tr><td rowspan="3">Nipponbare</td><td>On-target</td><td>Query 1 CCTTCAAGCAAAGCAAACTCAAG 23
|||||||||||||||||||||||
Sbjct 18174472 CCTTCAAGCAAAGCAAACTCAAG 18174450</td></tr>
<tr><td rowspan="2">Potential off-target</td><td>Query 4 TCAAGCAAAGCAAAC 18
|||||||||||||||
Sbjct 5019438 TCAAGCAAAGCAAAC 5019452</td></tr>
<tr><td>Query 2 CTTCAAGCAAAGCAAACT 19
||||||||| ||||||||
Sbjct 16189560 CTTCAAGCATAGCAAACT 16189543</td></tr>
<tr><td rowspan="4">Shuhui498</td><td>On-target</td><td>Query 1 CCTTCAAGCAAAGCAAACTCAAG 23
|||||||||||||||||||||||
Sbjct 20518344 CCTTCAAGCAAAGCAAACTCAAG 20518322</td></tr>
<tr><td rowspan="3">Potential off-target</td><td>Query 4 TCAAGCAAAGCAAAC 18
|||||||||||||||
Sbjct 5084435 TCAAGCAAAGCAAAC 5084449</td></tr>
<tr><td>Query 2 CTTCAAGCAAAGCAAACT 19
||||||||||| ||||||
Sbjct 10689740 CTTCAAGCAAAACAAACT 10689757</td></tr>
<tr><td>Query 2 CTTCAAGCAAAGCAAACT 19
||||||||| ||||||||
Sbjct 18250033 CTTCAAGCATAGCAAACT 18250016</td></tr>
<tr><td rowspan="4">RP Bio-226</td><td>On-target</td><td>Query 1 CCTTCAAGCAAAGCAAACTCAAG 23
|||||||||||||||||||||||
Sbjct 14010607 CCTTCAAGCAAAGCAAACTCAAG 14010585</td></tr>
<tr><td rowspan="3">Potential off-target</td><td>Query 4 TCAAGCAAAGCAAAC 18
|||||||||||||||
Sbjct 4545848 TCAAGCAAAGCAAAC 4545862</td></tr>
<tr><td>Query 2 CTTCAAGCAAAGCAAACT 19
||||||||||| ||||||
Sbjct 8625223 CTTCAAGCAAAACAAACT 8625240</td></tr>
<tr><td>Query 2 CTTCAAGCAAAGCAAACT 19
||||||||| ||||||||
Sbjct 12072704 CTTCAAGCATAGCAAACT 12072687</td></tr>
<tr><td colspan="2">gRNA6 (5'-3')</td><td>AACCAGGUGCUAAGCUCAUC</td></tr>
<tr><td colspan="2">Genomic target sequence</td><td>AACCAGGTGCTAAGCTCATCAAG</td></tr>
<tr><td rowspan="2">Nipponbare</td><td>On-target</td><td>Query 1 AACCAGGTGCTAAGCTCATCAAG 23
|||||||||||||||||||||||
Sbjct 18174495 AACCAGGTGCTAAGCTCATCAAG 18174473</td></tr>
<tr><td>Potential off-target</td><td>Query 4 CAGGTGCTAAGCTC 17
||||||||||||||
Sbjct 10170629 CAGGTGCTAAGCTC 10170642</td></tr>
<tr><td>Shuhui498</td><td>On-target</td><td>Query 1 AACCAGGTGCTAAGCTCATCAAG 23
|||||||||||||||||||||||</td></tr>
</table>

		Sbjct 20518367 AACCAGGTGCTAAGCTCATCAAG 20518345
	Potential off-target	Query 2 ACCAGGTGCTAAGC 15<br>|||||||||||||| Sbjct 17431714 ACCAGGTGCTAAGC 17431727
		Query 5 AGGTGCTAAGCTCATCA 21<br>||||||| ||||||||| Sbjct 8658144 AGGTGCTCAGCTCATCA 8658128

Supplementary Table 9. List of PCR primers used and their applications.

Primer	Sequence (5' to 3')	Purpose
g8N3P-F3	GCAGTTGGTGGTGTACAGTAGG	gRNA targeting *SWEET11* for promoter mutations
g8N3P-R3	AAACCCTACTGTACACCACCAA	
g12N3P-F3i	GCAGAGAGGAACGAAGGGAGTTG	gRNA targeting *SWEET13* for promoter mutations in *indica* rice
g12N3P-R3i	AAACCAACTCCCTTCGTTCCTCT	
g12N3P-F4j	GCAGAGAGGAATGAAGGGAGTTG	gRNA targeting *SWEET13* for promoter mutations in *japonica* rice
g12N3P-R4j	AAACCAACTCCCTTCATTCCTCT	
g11N3P-F4	GCAGTATATAAACCCCCTCCAACC	gRNA targeting *SWEET14* for promoter mutations in EBEs of AvrXa7 and PthXo3
g11N3P-R4	AAACGGTTGGAGGGGGTTTATATA	
g11N3P-F5	GCAGCTTGAGTTTGCTTTGCTTGA	gRNA targeting *SWEET14* for promoter mutations in EBE of TalF
g11N3P-R5	AAACTCAAGCAAAGCAAACTCAAG	
g11N3P-F6	GCAGAACCAGGTGCTAAGCTCATC	gRNA targeting *SWEET14* for promoter mutations in EBE of TalC
g11N3P-R6	AAACGATGAGCTTAGCACCTGGTT	
pTLN-Seq-F	TGGCCCGTGTCTCAAAATCTCTG	Sequence individual tRNA-gRNA units
pTLN-Seq-R	ATCTTTTCTACGGGGTCTGACG	
U6P-F1b	CGTTGAGGGGAGACAGGTTTAG	Sequence Golden Gate assembled tRNA-gRNA units
pENTR4-R	TGGGTCTAGATATCTCGAGTG	
8NKpI-F5	TGAGTGGTCATACGTGTCATATTG	Genotype *SWEET11* promoter mutations through PCR and sequencing
8N3G-R1	CATTGCTACTGGTGATGAAGGT	
12N3P-F2c	TACCCGTGCAAACAAAGAACAGAG	Genotype *SWEET13* promoter mutations through PCR and sequencing
gSWT13-R1	AAACGGGATGCTGCAGGGACAGGC	
RGT48-F	TCCCTTAACTAGGACAACTTGGA	Genotype *SWEET14* promoter mutations through PCR and sequencing
g11N3-R1	AAACGGGATGCTGAAGAGACATGC	
g8N3-F2	TGTTGTCACCAGTAGCAATGGCAGG	gRNA targeting *SWEET11* for knockout
g8N3-R2	AAACCCTGCCATTGCTACTGGTGAC	
gSWT13-F1	TGTTGCCTGTCCCTGCAGCATCCC	gRNA targeting *SWEET13* for knockout
gSWT13-R1	AAACGGGATGCTGCAGGGACAGGC	
g11N3-F1	GTGTGCATGTCTCTTCAGCATCCC	gRNA targeting *SWEET14* for knockout
g11N3-R1	AAACGGGATGCTGAAGAGACATGC	
8NKpI-F5	TGAGTGGTCATACGTGTCATATTG	Genotype *SWEET11* knockout mutations through PCR and sequencing
8N3-R2	AAGTGACTTGTGCCCATCACTTG	
SWT13-F	CTCCACAAAGGGGGTCTAGG	Genotype *SWEET13* knockout mutations through PCR and sequencing
SWT13-R	TACCATCCATATTGCCTTCG	
RGT48-F	TCCCTTAACTAGGACAACTTGGA	Genotype *SWEET14* knockout mutations through PCR and sequencing
3D-3008-R	GGAGCCCCTTTTCTTTTGAG	
HptF	AGCCTGACCTATTGCATCT	Genotype hygromycin resistance gene
HptR	CATATGAAATCACGCCATGT	
OsCas9-F	GGGTAATGAACTCGCTCTGC	Genotype Cas9 gene
OsCas9-R	TGGCGTCAAGAACTTCCTTTG	

OsRAc1-1128-F	TCCATCTTGGCATCTCTCAG	Positive control with rice actin gene for a PCR control
OsRAc1-1128-R	GTACCCTCATCAGGCATCTG	

Supplementary Table 10. Sanger sequencing results for vector construction

Sample name	BY8660 and BY8661
**Raw sequencing**	>C05_BY8660_U6P-F1_1000329.seq | Sequence #1 of 2 downloaded on Mon Aug 8 10:50:48 CDT 2016<br>ANNNNNNNNATTTNCAANNGGTTTTATTCATGAATGGTCACGATGGATGATGG<br>GGCTCAGACTTGAGCTACGAGGCCGCAGGCGAGAGAAGCCTAGTGTGCTCT<br>CTGCTTGTTTGGGCCGTAACGGAGGATACGGCCGACGAGCGTGTACTACCG<br>CGCGGGATGCCGCTGGGCGCTGCGGGGGCCGTTGGATGGGGATCGGTGGG<br>TCGCGGGAGCGTTGAGGGGAGACAGGTTTAGTACCACCTCGCCTACCGAAC<br>AATGAAGAACCCACCTTATAACCCCGCGCGCTGCCGCTTGTGTTGAACAAAG<br>CACCAGTGGTCTAGTGGTAGAATAGTACCCTGCCACGGTACAGACCCGGGTT<br>CGATTCCCGGCTGGTGCAGTTGGTGGTGTACAGTAGGGTTTTAGAGCTAGAA<br>ATAGCAAGTTAAAATAAGGCTAGTCCGTTATCAACTTGAAAAAGTGGCACCGA<br>GTCGGTGCGGGAGCACATGAGGATCACCCATGTGCCACGAGCGACATGAGG<br>ATCACCCATGTCGCTCGTGTTCCCAACAAAGCACCAGTGGTCTAGTGGTAGA<br>ATAGTACCCTGCCACGGTACAGACCCGGGTTCGATTCCCGGCTGGTGCAGA<br>GAGGAACGAAGGGAGTTGGTTTTAGAGCTAGAAATAGCAAGTTAAAATAAGG<br>CTAGTCCGTTATCAACTTGAAAAAGTGGCACCGAGTCGGTGCGGGAGCACAT<br>GAGGATCACCCATGTGCCACGAGCGACATGAGGATCACCCATGTCGCTCGT<br>GTTCCCAACAAAGCACCAGTGGTCTAGTGGTAGAATAGTACCCTGCCACGGT<br>ACAGACCCGGGTTCGATTCCCGGCTGGTGCAGAGGGCATGCATGTCAGCAG<br>CGTTTTAGAGCTAGAAATAGCAAGTTAAAATAAGGCTAGTCCGTTATCAACTT<br>GAAAAAGTGGCACCGAGTCGGTGCGGGAGCACATGAGGATCACCCATGTGC<br>CACGAGCGACATGAGGATCACCCATGTCGCTCGTGTTCCCAACAAAGCACCA<br>GTGGTCTAGTGGTAGAATAGTACCCTGCCACGGTACAGACCCGGGTTCGATT<br>CCCGGCTGGTGCAGTATATAAACCCCCTCCCACCGTTTTAAAGCTAGAAATA<br>GCCAGGTTAAAATAAGGGCTAGTCCCGTTATCA
	>D05_BY8661_pENTR4-R_1000330.seq | Sequence #2 of 2 downloaded on Mon Aug 8 10:50:48 CDT 2016<br>NNNNNNTNNAANTNAGCTTATCGATACCGTCGACCTCGAGCGGCCGCCAGT<br>GTGATGGATATCTGCAGAATTGCCCTTCGAAGGGACAAAAAAAGCACCGACT<br>CGGTGCCACTTTTTCAAGTTGATAACGGACTAGCCTTATTTTAACTTGCTATTT<br>CTAGCTCTAAAACCGGCCGCCAGTGTGATGGATATCTGCAGAATTGCCCTTC<br>GAAGGGACAAAAAAAGGGAACACGAGCGACATGGGTGATCCTCATGTCGCT<br>CGTGGCACATGGGTGATCCTCATGTGCTCCCGCACCGACTCGGTGCCACTTT<br>TTCAAGTTGATAACGGACTAGCCTTATTTTAACTTGCTATTTCTAGCTCTAAAA<br>CGATGAGCTTAGCACCTGGTTCTGCACCAGCCGGGAATCGAACCCGGGTCT<br>GTACCGTGGCAGGGTACTATTCTACCACTAGACCACTGGTGCTTTGTTGGGA<br>ACACGAGCGACATGGGTGATCCTCATGTCGCTCGTGGCACATGGGTGATCCT<br>CATGTGCTCCCGCACCGACTCGGTGCCACTTTTTCAAGTTGATAACGGACTA<br>GCCTTATTTTAACTTGCTATTTCTAGCTCTAAAACTCAAGCAAAGCAAACTCAA<br>GCTGCACCAGCCGGGAATCGAACCCGGGTCTGTACCGTGGCAGGGTACTAT<br>TCTACCACTAGACCACTGGTGCTTTGTTGGGAACACGAGCGACATGGGTGAT<br>CCTCATGTCGCTCGTGGCACATGGGTGATCCTCATGTGCTCCCGCACCGACT<br>CGGTGCCACTTTTTCAAGTTGATAACGGACTAGCCTTATTTTAACTTGCTATTT<br>CTAGCTCTAAAACGGTTGGGAGGGGGTTTATATACTGCACCAGCCGGGAATC<br>GAACCCGGGTCTGTACCGTGGCAGGGTACTATTCTACCACTAGACCACTGGT<br>GCTTTGTTGGGAACACGAGCGACATGGGGTGATCCTCATGTCGCTCGTGGCA<br>CATGGGGTGATCCTCATGTGGCTCCCGCACCGACTCGGNGCCACTTTTTTCA<br>AGTTGANNACGGGACTAGCCCTTATTTTAACTTGGCTATTTTCTAGCTTCTAAA<br>AACGCTGGCTGAAATGGCATGCCCCTCTGGCACCAGCCGGGAAATCGAAAC<br>CCGGGGTCTGGTACCGGTGGGCAGGGTTACTAATTCTAACCACTAAAACCAC<br>CTGGGTG
**Note**	To sequence the guide RNA cassette of 6 guide RNAs in pENTR clone from both ends. This clone was used to Gateway into Cas9 binary vector for Kitaake

	transformation (see Supplementary Fig. 6)
**Sample name**	BY6968 and BY6969
**Raw sequencing**	>A03_BY6968_U6P-F1b_932385.seq | Sequence #3 of 4 downloaded on Tue Mar 24 15:21:05 CDT 2015<br>NNNNNNNNGANATGAGACCACCTTATAACCCCGCGCGCTGCCGCTTGTGTTG<br>TTGGTGGTGTACAGTAGGGTTTTAGAGCTAGGCCAACATGAGGATCACCCAT<br>GTCTGCAGGGCCTAGCAAGTTAAAATAAGGCTAGTCCGTTATCAACTTGGCC<br>AACATGAGGATCACCCATGTCTGCAGGGCCAAGTGGCACCGAGTCGGTGCT<br>TTAACAAGCACCAGTGGTCTAGTGGTAGAATAGTACCCTGCCACGGTACAGA<br>CCCGGGTTCGATTCCCGGCTGGTGCAGGAGAGGAACGAAGGGAGTTGGTTT<br>TAGAGCTAGGCCAACATGAGGATCACCCATGTCTGCAGGGCCTAGCAAGTTA<br>AAATAAGGCTAGTCCGTTATCAACTTGGCCAACATGAGGATCACCCATGTCTG<br>CAGGGCCAAGTGGCACCGAGTCGGTGCTTATAAAAGGCCTATTAGCTCAGCT<br>GGTTAGAGCGTCGTGCTAATAACGCGAAGGTCGCAGGTTCGTGACCTGCATG<br>GGCCAGAGGGCATGCATGTCAGCAGCGTTTTAGAGCTAGGCCAACATGAGG<br>ATCACCCATGTCTGCAGGGCCTAGCAAGTTAAAATAAGGCTAGTCCGTTATCA<br>ACTTGGCCAACATGAGGATCACCCATGTCTGCAGGGCCAAGTGGCACCGAG<br>TCGGTGCTTCAATAATCCGTTGTAGTCTAGCTGGTTAGGATACTCGGCTCTCA<br>CCCGAGCGACCCGGGTTCGAGTCCCGGCAACGGAAGTATATAAACCCCCTC<br>CAACCGTTTTAGAGCTAGGCCAACATGAGGATCACCCATGTCTGCAGGGCCT<br>AGCAAGTTAAAATAAGGCTAGTCCGTTATCAACTTGGCCAACATGAGGATCAC<br>CCATGTCTGCAGGGCCAAGTGGCACCGAGTCGGTGCTTTTTTTATGGTTTTA<br>GAGCTAGAAATAGCAAGTTAAAATAAGGCTAGTCCGTTATCAACTTGAAAAAG<br>TGGCACCGAGTCGGTGCTTTTTTTGTCCCTTCGAAGGGCAATTCTGCAGATAT<br>CCATCACACTGGCGGCCGCTCGAGGTCGACGGTATCGATAAGCTTGAWATC<br>GAATTCSCGGCCSCMCTCGAGAWWTCTAGACCCAGCTTTCTTKGTACAAAGT<br>TGGCAWTTATAARAAAAGCATTGCTTATCAATTTGTTGCAACGAAMAGGGTC<br>MCTWTCAGTCAAAATAAAATCATWATTTGCCATCCAGCTGSAGSTYTGGGCC<br>GGGGTYYCAAAAYYYYYGAAGKTTMMMTTGSCCAAAGATAAAAAAWWWTTYY<br>YCCTKGAAA
	>B03_BY6969_pENTR4-R_932386.seq | Sequence #4 of 4 downloaded on Tue Mar 24 15:21:05 CDT 2015<br>NNNNNTNATCAGCTTATCGATACCGTCGACCTCGAGCGGCCGCCAGTGTGAT<br>GGATATCTGCAGAATTGCCCTTCGAAGGGACAAAAAAAGCACCGACTCGGTG<br>CCACTTTTTCAAGTTGATAACGGACTAGCCTTATTTTAACTTGCTATTTCTAGC<br>TCTAAAACCATAAAAAAAGCACCGACTCGGTGCCACTTGGCCCTGCAGACAT<br>GGGTGATCCTCATGTTGGCCAAGTTGATAACGGACTAGCCTTATTTTAACTTG<br>CTAGGCCCTGCAGACATGGGTGATCCTCATGTTGGCCTAGCTCTAAAACGGT<br>TGGAGGGGGTTTATATACTTCCGTTGCCGGGACTCGAACCCGGGTCGCTCG<br>GGTGAGAGCCGAGTATCCTAACCAGCTAGACTACAACGGATTATTGAAGCAC<br>CGACTCGGTGCCACTTGGCCCTGCAGACATGGGTGATCCTCATGTTGGCCAA<br>GTTGATAACGGACTAGCCTTATTTTAACTTGCTAGGCCCTGCAGACATGGGTG<br>ATCCTCATGTTGGCCTAGCTCTAAAACGCTGCTGACATGCATGCCCTCTGGC<br>CCATGCAGGTCACGAACCTGCGACCTTCGCGTTATTAGCACGACGCTCTAAC<br>CAGCTGAGCTAATAGGCCTTTTATAAGCACCGACTCGGTGCCACTTGGCCCT<br>GCAGACATGGGTGATCCTCATGTTGGCCAAGTTGATAACGGACTAGCCTTAT<br>TTTAACTTGCTAGGCCCTGCAGACATGGGTGATCCTCATGTTGGCCTAGCTCT<br>AAAACCAACTCCCTTCGTTCCTCTCCTGCACCAGCCGGGAATCGAACCCGGG<br>TCTGTACCGTGGCAGGGTACTATTCTACCACTAGACCACTGGTGCTTGTTAAA<br>GCACCGACTCGGTGCCACTTGGCCCTGCAGACATGGGTGATCCTCATGTTG<br>GCCAAGTTGATAACGGACTAGCCTTATTTTAACTTGCTAGGCCCTGCAGACAT<br>GGGTGATCCTCATGTTGGCCTAGCTCTAAAACCCTACTGTACACCACCAACAA<br>CACAAGCGGCAGCGCGCGGGGGTTWTAAGGGGGGGTTCTTCATTGTTCGGT<br>AGGCGAGGTGGTACTAAACCTGGTCTCCCCTCAAAGCTCCCGSGRACCCAC<br>CGATTCCCAATCAAAAGGCCCCCGGAAGSGCCCAAGGGGAATCCCGCSSGG

	GWAGTAMMMGCYYCGTCGGGCCGWATCCYTCCGGTTACGGGCCCAAACAA AGCCNAAARRGCCMMCTTAGGGYTTCTCTTNGCCTGGGGGGCCTCCTAAGC YYAAAGKCTTGAAGCCCCCATCATTCCAWCGGGGGGCC
**Note**	To sequence the guide RNA cassette of 4 guide RNAs in pENTR clone from both ends. This clone was used to Gateway into Cas9 binary vector for transformation of IR64 and Ciherang-Sub1 (see Supplementary Fig. 9)

Supplementary Table 11. Access to Xoo strains.

Strain	Country	Continent	Source	to be requested from
**A3842**	India	Asia	MU	Zhongchao Yin (TLL)
**A3857**	India	Asia	MU	Zhongchao Yin (TLL)
**Aust-2013**	Australia	Oceania	MU	Zhongchao Yin (TLL)
**Aust-R3**	Australia	Oceania	MU	Zhongchao Yin (TLL)
**AXO1947**	Cameroun	Africa	MU	Zhongchao Yin (TLL)
**BAI3**	Burkina Faso	Africa	IRD	CIRM-Plant Associated Bacteria / CIRM-CFBP
**C1**	China	Asia	MU	Zhongchao Yin (TLL)
**C3**	China	Asia	MU	Zhongchao Yin (TLL)
**C4**	China	Asia	MU	Zhongchao Yin (TLL)
**C5**	China	Asia	MU	Zhongchao Yin (TLL)
**C6**	China	Asia	MU	Zhongchao Yin (TLL)
**C7**	China	Asia	MU	Zhongchao Yin (TLL)
**CFBP1948**	Cameroun	Africa	IRD	CIRM-Plant Associated Bacteria / CIRM-CFBP
**CFBP1949**	Mali	Africa	IRD	CIRM-Plant Associated Bacteria / CIRM-CFBP
**CFBP1951**	Mali	Africa	IRD	CIRM-Plant Associated Bacteria / CIRM-CFBP
**CFBP1952**	Mali	Africa	IRD	CIRM-Plant Associated Bacteria / CIRM-CFBP
**CFBP7319**	Burkina Faso	Africa	IRD	CIRM-Plant Associated Bacteria / CIRM-CFBP
**CFBP7320**	Burkina Faso	Africa	IRD	CIRM-Plant Associated Bacteria / CIRM-CFBP
**CFBP7322**	Burkina Faso	Africa	IRD	CIRM-Plant Associated Bacteria / CIRM-CFBP
**CFBP7323**	Niger	Africa	IRD	CIRM-Plant Associated Bacteria / CIRM-CFBP
**CFBP7324**	Niger	Africa	IRD	CIRM-Plant Associated Bacteria / CIRM-CFBP
**CFBP7337**	Mali	Africa	IRD	CIRM-Plant Associated Bacteria / CIRM-CFBP
**CFBP7340**	Niger	Africa	IRD	CIRM-Plant Associated Bacteria / CIRM-CFBP
**CFBP8172**	Benin	Africa	IRD	CIRM-Plant Associated Bacteria / CIRM-CFBP
**CIAT1185**	Colombia	America	MU	Zhongchao Yin (TLL)
**CIX1041**	Ivory Coast	Africa	IRD	Boris Szurek (IRD)
**CIX2374**	Senegal	Africa	IRD	Boris Szurek (IRD)
**CIX611**	Benin	Africa	IRD	Boris Szurek (IRD)
**CIX629**	Niger	Africa	IRD	Boris Szurek (IRD)

**Dak16**	Tanzania	Africa	MU	Andreas von Tiedemann (FHE)
**GD1358**	China	Asia	MU	Zhongchao Yin (TLL)
**H75373**	Japan	Asia	MU	Zhongchao Yin (TLL)
**HB17**	China	Asia	MU	Zhongchao Yin (TLL)
**HB21**	China	Asia	MU	Zhongchao Yin (TLL)
**HLJ72**	China	Asia	MU	Zhongchao Yin (TLL)
**HuN37**	China	Asia	DR	Lifang Ruan (HAU)
**IX-280**	India	Asia	MU	Zhongchao Yin (TLL)
**IX056**	Indonesia	Asia	MU	Zhongchao Yin (TLL)
**JL25**	China	Asia	DR	Lifang Ruan (HAU)
**JL28**	China	Asia	DR	Lifang Ruan (HAU)
**JL33**	China	Asia	DR	Lifang Ruan (HAU)
**JP01**	Japan	Asia	DR	Lifang Ruan (HAU)
**JW11089**	Korea	Asia	MU	Zhongchao Yin (TLL)
**JW89011**	Korea	Asia	MU	Zhongchao Yin (TLL)
**JX01**	Japan	Asia	MU	Zhongchao Yin (TLL)
**K202**	Korea	Asia	MU	Zhongchao Yin (TLL)
**KACC10331**	Korea	Asia	DR	BM Lee (NIAB)
**KXO85**	Korea	Asia	MU	Zhongchao Yin (TLL)
**LN57**	China	Asia	MU	Zhongchao Yin (TLL)
**MAFF311018**	Japan	Asia	DR	Hirokazu Ochiai (NIAS)
**MAI1**	Mali	Africa	IRD	CIRM-Plant Associated Bacteria / CIRM-CFBP
**MAI106**	Mali	Africa	IRD	Boris Szurek (IRD)
**MAI129**	Mali	Africa	IRD	Boris Szurek (IRD)
**MAI134**	Mali	Africa	IRD	Boris Szurek (IRD)
**MAI145**	Mali	Africa	IRD	Boris Szurek (IRD)
**MAI68**	Mali	Africa	IRD	Boris Szurek (IRD)
**MAI73**	Mali	Africa	IRD	Boris Szurek (IRD)
**MAI95**	Mali	Africa	IRD	Boris Szurek (IRD)
**MAI99**	Mali	Africa	IRD	Boris Szurek (IRD)
**ME2**	NA	NA	MU	Bing Yang (MU)
**ME2(*PthXo2*)**	NA	NA	MU	Bing Yang (MU)
**ME2(P*thXo2B*)**	NA	NA	MU	Bing Yang (MU)
**ME2(*TalC*)**	NA	NA	MU	Bing Yang (MU)
**NX260**	Nepal	Asia	MU	Zhongchao Yin (TLL)
**NX42**	China	Asia	MU	Zhongchao Yin (TLL)
**NXO260**	Nepal	Asia	MU	Zhongchao Yin (TLL)
**PXO101**	Philippines	Asia	IRRI	Ricardo Oliva (IRRI)

**PXO102**	Philippines	Asia	IRRI	Ricardo Oliva (IRRI)
**PXO112**	Philippines	Asia	IRRI	Ricardo Oliva (IRRI)
**PXO123**	Philippines	Asia	IRRI	Ricardo Oliva (IRRI)
**PXO124**	Philippines	Asia	IRRI	Ricardo Oliva (IRRI)
**PXO125**	Philippines	Asia	IRRI	Ricardo Oliva (IRRI)
**PXO127**	Philippines	Asia	IRRI	Ricardo Oliva (IRRI)
**PXO128**	Philippines	Asia	IRRI	Ricardo Oliva (IRRI)
**PXO129**	Philippines	Asia	IRRI	Ricardo Oliva (IRRI)
**PXO144**	Philippines	Asia	IRRI	Ricardo Oliva (IRRI)
**PXO145**	Philippines	Asia	IRRI	Ricardo Oliva (IRRI)
**PXO148**	Philippines	Asia	IRRI	Ricardo Oliva (IRRI)
**PXO163**	Philippines	Asia	IRRI	Ricardo Oliva (IRRI)
**PXO190**	Philippines	Asia	IRRI	Ricardo Oliva (IRRI)
**PXO192**	Philippines	Asia	IRRI	Ricardo Oliva (IRRI)
**PXO198**	Philippines	Asia	IRRI	Ricardo Oliva (IRRI)
**PXO200**	Philippines	Asia	IRRI	Ricardo Oliva (IRRI)
**PXO202**	Philippines	Asia	IRRI	Ricardo Oliva (IRRI)
**PXO203**	Philippines	Asia	IRRI	Ricardo Oliva (IRRI)
**PXO205**	Philippines	Asia	IRRI	Ricardo Oliva (IRRI)
**PXO208**	Philippines	Asia	IRRI	Ricardo Oliva (IRRI)
**PXO210**	Philippines	Asia	IRRI	Ricardo Oliva (IRRI)
**PXO211**	Philippines	Asia	IRRI	Ricardo Oliva (IRRI)
**PXO212**	Philippines	Asia	IRRI	Ricardo Oliva (IRRI)
**PXO220**	Philippines	Asia	IRRI	Ricardo Oliva (IRRI)
**PXO236**	Philippines	Asia	IRRI	Ricardo Oliva (IRRI)
**PXO240**	Philippines	Asia	IRRI	Ricardo Oliva (IRRI)
**PXO250**	Philippines	Asia	IRRI	Ricardo Oliva (IRRI)
**PXO280**	Philippines	Asia	IRRI	Ricardo Oliva (IRRI)
**PXO282**	Philippines	Asia	IRRI	Ricardo Oliva (IRRI)
**PXO285**	Philippines	Asia	IRRI	Ricardo Oliva (IRRI)
**PXO312**	Philippines	Asia	IRRI	Ricardo Oliva (IRRI)
**PXO324**	Philippines	Asia	IRRI	Ricardo Oliva (IRRI)
**PXO338**	Philippines	Asia	IRRI	Ricardo Oliva (IRRI)
**PXO339**	Philippines	Asia	IRRI	Ricardo Oliva (IRRI)
**PXO340**	Philippines	Asia	IRRI	Ricardo Oliva (IRRI)
**PXO341**	Philippines	Asia	IRRI	Ricardo Oliva (IRRI)
**PXO342**	Philippines	Asia	IRRI	Ricardo Oliva (IRRI)
**PXO343**	Philippines	Asia	IRRI	Ricardo Oliva (IRRI)
**PXO344**	Philippines	Asia	IRRI	Ricardo Oliva (IRRI)

**PXO345**	Philippines	Asia	IRRI	Ricardo Oliva (IRRI)
**PXO346**	Philippines	Asia	IRRI	Ricardo Oliva (IRRI)
**PXO347**	Philippines	Asia	IRRI	Ricardo Oliva (IRRI)
**PXO349**	Philippines	Asia	IRRI	Ricardo Oliva (IRRI)
**PXO363**	Philippines	Asia	IRRI	Ricardo Oliva (IRRI)
**PXO364**	Philippines	Asia	IRRI	Ricardo Oliva (IRRI)
**PXO368**	Philippines	Asia	IRRI	Ricardo Oliva (IRRI)
**PXO372**	Philippines	Asia	IRRI	Ricardo Oliva (IRRI)
**PXO404**	Philippines	Asia	IRRI	Ricardo Oliva (IRRI)
**PXO404**	Philippines	Asia	IRRI	Ricardo Oliva (IRRI)
**PXO421**	Philippines	Asia	IRRI	Ricardo Oliva (IRRI)
**PXO513**	Philippines	Asia	IRRI	Ricardo Oliva (IRRI)
**PXO516**	Philippines	Asia	IRRI	Ricardo Oliva (IRRI)
**PXO524**	Philippines	Asia	IRRI	Ricardo Oliva (IRRI)
**PXO563**	Philippines	Asia	IRRI	Ricardo Oliva (IRRI)
**PXO568**	Philippines	Asia	IRRI	Ricardo Oliva (IRRI)
**PXO572**	Philippines	Asia	IRRI	Ricardo Oliva (IRRI)
**PXO582**	Philippines	Asia	IRRI	Ricardo Oliva (IRRI)
**PXO589**	Philippines	Asia	IRRI	Ricardo Oliva (IRRI)
**PXO595**	Philippines	Asia	IRRI	Ricardo Oliva (IRRI)
**PXO599**	Philippines	Asia	IRRI	Ricardo Oliva (IRRI)
**PXO602**	Philippines	Asia	IRRI	Ricardo Oliva (IRRI)
**PXO603**	Philippines	Asia	IRRI	Ricardo Oliva (IRRI)
**PXO604**	Philippines	Asia	IRRI	Ricardo Oliva (IRRI)
**PXO605**	Philippines	Asia	IRRI	Ricardo Oliva (IRRI)
**PXO606**	Philippines	Asia	IRRI	Ricardo Oliva (IRRI)
**PXO607**	Philippines	Asia	IRRI	Ricardo Oliva (IRRI)
**PXO608**	Philippines	Asia	IRRI	Ricardo Oliva (IRRI)
**PXO609**	Philippines	Asia	IRRI	Ricardo Oliva (IRRI)
**PXO61**	Philippines	Asia	IRRI	Ricardo Oliva (IRRI)
**PXO610**	Philippines	Asia	IRRI	Ricardo Oliva (IRRI)
**PXO611**	Philippines	Asia	IRRI	Ricardo Oliva (IRRI)
**PXO612**	Philippines	Asia	IRRI	Ricardo Oliva (IRRI)
**PXO69**	Philippines	Asia	IRRI	Ricardo Oliva (IRRI)
**PXO70**	Philippines	Asia	IRRI	Ricardo Oliva (IRRI)
**PXO71**	Philippines	Asia	IRRI	Ricardo Oliva (IRRI)
**PXO79**	Philippines	Asia	IRRI	Ricardo Oliva (IRRI)
**PXO83**	Philippines	Asia	IRRI	Ricardo Oliva (IRRI)
**PXO86**	Philippines	Asia	IRRI	Ricardo Oliva (IRRI)

**PXO99**	Philippines	Asia	IRRI	Ricardo Oliva (IRRI)
**PXO99[A]**	Philippines	Asia	IRRI	Jan Leach (CSU)
**R-7**	Thailand	Asia	MU	Zhongchao Yin (TLL)
**ScYc-b**	China	Asia	DR	Lifang Ruan (HAU)
**SK2-3**	Thailand	Asia	KA	Sujin Patarapuwadol (KU)
**T19**	Tanzania	Africa	MU	Andreas von Tiedemann (FHE)
**T7174**	Japan	Asia	MU	Zhongchao Yin (TLL)
**Ug11**	Uganda	Africa	MU	Andreas von Tiedemann (FHE)
**XOO2**	Thailand	Asia	MU	Zhongchao Yin (TLL)
**XF89b**	Taiwan	Asia	DR	Pao-Jen Ann (TARI)
**XM9**	Taiwan	Asia	DR	Pao-Jen Ann (TARI)
**Xoc_BLS256**	Philippines	Asia	IRRI	Ricardo Oliva (IRRI)
**YC11**	China	Asia	DR	Lifang Ruan (HAU)
**ZHE173**	China	Asia	MU	Zhongchao Yin (TLL)

MU, University of Missouri, USA (material transfer is processed according to the US regulation of Select Agent)
IRD, Institute de Recherche pour le Développement, France
IRRI, International Rice Research Institute, The Philippines
CSU, Colorado State University, USA
KA, Kasetsart University, Thailand
TLL, TEMASEK LifeSciences Laboratory, Singapore
CIRM-CFBP, the International Center for Microbial Resources – Bacteria Associated with Plants, France
FHE, University of Applied Sciences Erfurt, Germany.
NIAB, National Institute of Agricultural Biotechnology, Republic of Korea
NIAS, National Institute of Agrobiological Sciences, Japan
HAU, Huazhong Agricultural University, China
TARI, Taiwan Agricultural Research Institute
DR, digital repository, e.g. NCBI
NA, not applicable

Supplementary Note 1: Biological Material Transfer Agreement

BIOLOGICAL MATERIAL TRANSFER AGREEMENT

THIS MATERIAL TRANSFER AGREEMENT ("AGREEMENT") is entered into as of the date of last signature of this AGREEMENT (the "EFFECTIVE DATE") between _________________ **("PROVIDER"),** whose address ___________________; and

_________________ (**"RECIPIENT")**, whose address is ___________________.

PROVIDER SCIENTIST:<br><br>Name: _______________<br><br>Address: _______________	RECIPIENT SCIENTIST:<br><br>Name: ____________________<br><br>Address: __________________

MATERIAL [Description of Biological Material to be transferred by PROVIDER to RECIPIENT; pls state in detail]:

____

The “MATERIAL” includes (a) the original MATERIAL, (b) any MATERIAL contained or incorporated in MODIFICATIONS, (c) PROGENY and (d) UNMODIFIED DERIVATIVES. The MATERIAL shall not include (a) MODIFICATIONS (except MATERIAL included therein), or (b) other substances created by the RECIPIENT through the use of the MATERIAL, which are not MODIFICATIONS, PROGENY or UNMODIFIED DERIVATIVES. As used in this AGREEMENT, "PROGENY" means an unmodified descendant from the MATERIAL, such as virus from virus, cell from cell or organism from organism; "UNMODIFIED DERIVATIVES" means substances created by the RECIPIENT which constitute an unmodified functional subunit or product expressed by the MATERIAL (e.g. subclones of unmodified cell lines, purified or fractionated subsets of the MATERIAL, proteins expressed by DNA/RNA supplied by the PROVIDER, or monoclonal antibodies secreted by a hybridoma cell line); and "MODIFICATIONS" means substances created by the RECIPIENT which contain/incorporate MATERIAL.

RESEARCH: The research to be conducted by the RECIPIENT SCIENTIST using the MATERIAL is described in Appendix A attached hereto (the "RESEARCH").

TERMS AND CONDITIONS

1. **Ownership/Use**. The MATERIAL is the property of PROVIDER. Nothing contained in this AGREEMENT shall restrict, modify or limit any ownership rights of the PROVIDER. The MATERIAL is to be used by RECIPIENT solely for the RESEARCH to be conducted by the RECIPIENT SCIENTIST. The RECIPIENT agrees that the MATERIAL will not be used in human subjects, in clinical trials, or for diagnostic purposes involving human subjects without the written consent of the PROVIDER; is to be used only at the RECIPIENT organisation and only in the RECIPIENT SCIENTIST'S laboratory under the direction of the RECIPIENT SCIENTIST or others working under RECIPIENT SCIENTIST'S direct supervision; will not be used for any commercial purpose or in connection with any commercially-sponsored research and will not be transferred to anyone else (including anyone else within the RECIPIENT'S organisation) without the prior written consent of the PROVIDER; will not be sequenced or otherwise analyzed in order to determine its structure or composition. RECIPIENT agrees to use the MATERIAL in compliance with all applicable statutes and regulations.

2. **Confidentiality**. The MATERIAL as well as all related information provided to RECIPIENT or RECIPIENT SCIENTIST shall constitute the confidential and/or proprietary information of PROVIDER (the "CONFIDENTIAL INFORMATION"). RECIPIENT and RECIPIENT SCIENTIST agree to maintain the confidentiality of the CONFIDENTIAL INFORMATION and to possess and use the CONFIDENTIAL INFORMATION solely for the purposes set forth in this AGREEMENT. As used in this AGREEMENT, CONFIDENTIAL INFORMATION shall not mean or include any information which (a) was known to RECIPIENT or RECIPIENT SCIENTIST prior to the receipt of the MATERIAL or the information from PROVIDER; (b) becomes known to the public without any breach of this AGREEMENT by RECIPIENT or RECIPIENT SCIENTIST; (c) is acquired from a third party without breach of any obligation of confidentiality; (d) is developed independently by RECIPIENT or RECIPIENT SCIENTIST without reference to or reliance on any CONFIDENTIAL INFORMATION; or (e) is required to be disclosed pursuant to subpoena or other judicial or administrative order, or pursuant to applicable law. The obligations of confidentiality contained in this section shall continue, as to any item of CONFIDENTIAL INFORMATION for a period of five (5) years from the date of receipt of such CONFIDENTIAL INFORMATION and shall survive the termination or expiration of this AGREEMENT for any reason.

3. **Availability.** RECIPIENT agrees to refer to PROVIDER any requests for the MATERIAL from other scientists. At its sole discretion, RECIPIENT will make the MATERIAL available under a separate AGREEMENT to other scientists for teaching or not-for-profit research purposes only.

4. **Report**. RECIPIENT and RECIPIENT SCIENTIST agree to submit to PROVIDER a written report describing the data generated in the course of the RESEARCH and setting out the results of the RESEARCH (together with the data, the "RESULTS") within six (6) months after the expiration or the termination of this AGREEMENT, whichever is the first to occur. PROVIDER is hereby granted a non-exclusive, royalty-free, perpetual right and license to use the RESULTS for internal academic and research purposes only.

5. **Publication**. In case of joint research, research results shall be published jointly and in mutual consent. If the RESEARCH conducted solely by the RECIPIENT SCIENTIST results in publication, the RECIPIENT SCIENTIST agrees to provide appropriate acknowledgment of the source of the MATERIAL and/or to give credit to PROVIDER or PROVIDER SCIENTIST, as

scientifically appropriate, for any contribution which PROVIDER or PROVIDER SCIENTIST may make to the subject matter of the publication. The RECIPIENT SCIENTIST shall give due consideration to the concerns of the PROVIDER when making scientific publications that relate to the subject matter of the AGREEMENT and, as such, will send the text of the intended publication to PROVIDER before it is published to give the PROVIDER an opportunity to review and comment on it in order to avoid having any applications for IP rights jeopardised by any prior publications that would compromise the novelty thereof or reveal any trade secrets. No later than four weeks after submitting the intended publication to the PROVIDER, and to the extent the PROVIDER has not expressed any objection thereto, the RECIPIENT may publish the findings in accordance with sentence 2. Should the PROVIDER raise any objections, the Parties shall endeavour to arrive at an amicable solution to enable publication.

6. **No Warranty**. The MATERIAL is understood to be experimental in nature and may have hazardous properties. The PROVIDER MAKES NO REPRESENTATIONS AND EXTENDS NO WARRANTIES OF ANY KIND, EITHER EXPRESSED OR IMPLIED, WITH RESPECT TO THE MATERIAL. THERE ARE NO EXPRESS OR IMPLIED WARRANTIES OF MERCHANTABILITY OR FITNESS FOR A PARTICULAR PURPOSE, OR THAT THE USE OF THE MATERIAL WILL NOT INFRINGE ANY PATENT, COPYRIGHT, TRADEMARK, OR OTHER PROPRIETARY RIGHTS.

7. **Liability**. Except to the extent prohibited by law, the RECIPIENT assumes all liability for damages which may arise from its use, storage or disposal of the MATERIAL. The PROVIDER will not be liable to the RECIPIENT for any loss, claim or demand made by the RECIPIENT, or made against the RECIPIENT by any other party, due to or arising from the use of the MATERIAL by the RECIPIENT.

8. **Inventions**. Any patentable invention which relates to new uses of the MATERIAL or which could not have been made but for the contribution of the MATERIAL (an "INVENTION"), will be jointly owned by PROVIDER and RECIPIENT. Any revenues arising from any use or implementation of such INVENTION will be shared by the PROVIDER and the RECIPIENT, with their respective shares to be negotiated in good faith and based on the relative contribution made by the MATERIAL to the INVENTION.

9. **Rights and Licenses**. The RECIPIENT acknowledges that the MATERIAL is or may be the subject of a patent application. Except as provided in this AGREEMENT, no express or implied licenses or other rights are provided to the RECIPIENT under any patents, patent applications, trade secrets or other proprietary rights of the PROVIDER, including any altered forms of the MATERIAL made by the PROVIDER. In particular, no express or implied licenses or other rights are provided to use the MATERIAL or any related patents of the PROVIDER for sale, lease, license, contract research or any other activities directly or indirectly resulting in commercial exploitation (“COMMERCIAL PURPOSES”). If the RECIPIENT desires to use or license the MATERIAL for COMMERCIAL PURPOSES, the RECIPIENT agrees, in advance of such use, to negotiate in good faith with the PROVIDER to establish the terms of a commercial license. It is understood by the RECIPIENT that the PROVIDER shall have no obligation to grant such a license to the RECIPIENT, and may grant exclusive or non-exclusive commercial licenses to others, or sell or assign all or part of the rights in the MATERIAL to any third parties, subject to any pre-existing rights held by others.

10. **Costs**. The MATERIAL is provided at no cost (if no amount is stated below) or for the optional transmittal fee stated below solely to reimburse the PROVIDER for preparation and distribution costs. Costs: _____; Transmittal Fee: _____.

11. **Termination**. Either PROVIDER or RECIPIENT may terminate this AGREEMENT upon the giving of at least thirty (30) days advance written notice. If not sooner terminated, this AGREEMENT shall expire one (1) year after the EFFECTIVE DATE. Upon expiration or termination of this AGREEMENT, RECIPIENT shall, if so directed by PROVIDER, return any unused portions of the MATERIAL or destroy the MATERIAL. Sections 2, 4, 5, 6, 7, 8 and 9 shall survive termination or expiration.

12. **No Assignment**. RECIPIENT shall have no right to assign or otherwise transfer any of its rights in this AGREEMENT or delegate any of its duties under this AGREEMENT without the written consent of PROVIDER.

13. **Governing Law**. General principles of international law shall apply including the UNIDROIT Principles of International Commercial Contracts of 2016. All disputes, controversy or claims arising in connection with this Agreement or its breach, termination or invalidity shall upon mutual agreement, be finally settled in accordance with the Arbitration Rules of UNCITRAL as at present in force, without recourse to the ordinary courts of law.

14. **Miscellaneous**. Any amendments to this AGREEMENT must be executed in writing. The foregoing shall also apply to any waiver of the writing requirement. Should any provision of this AGREEMENT be or become invalid, this shall not affect the validity of the remaining provisions hereof; instead PROVIDER and RECIPIENT agree to replace the invalid provision with a valid provision that most closely reflects the economic intent of the invalid provision.

IN WITNESS THEREOF, the PROVIDER and RECIPIENT have entered into this AGREEMENT as of the EFFECTIVE DATE.

PROVIDER<br><br>________________<br><br>By: ______________________________<br>[Signature]<br><br>Name: ____________________________<br>[Printed name]<br><br>Title: _____________________________<br><br>Date: _____________________________	RECIPIENT<br><br>____________________<br><br>By: ______________________________<br>[Signature]<br><br>Name: ____________________________<br>[Printed name]<br><br>Title: _____________________________<br><br>Date: _____________________________
As PROVIDER SCIENTIST, I acknowledge and accept the terms and provisions of this AGREEMENT:	As RECIPIENT SCIENTIST, I acknowledge and accept the terms and provisions of this AGREEMENT:

______________________________<br>[Signature]	______________________________<br>[Signature]

APPENDIX A

The RESEARCH

[Please state in detail]

Supplementary Note 2: Standard Material Transfer Agreement (SMTA) / Additional Terms and Conditions to SMTA

STANDARD MATERIAL TRANSFER AGREEMENT*

PREAMBLE

WHEREAS

The International Treaty on Plant Genetic Resources for Food and Agriculture (hereinafter referred to as "the **Treaty**")[1] was adopted by the Thirty-first session of the FAO Conference on 3 November 2001 and entered into force on 29 June 2004;

The objectives of the **Treaty** are the conservation and sustainable use of **Plant Genetic Resources for Food and Agriculture** and the fair and equitable sharing of the benefits arising out of their use, in harmony with the Convention on Biological Diversity, for sustainable agriculture and food security*;*

The Contracting Parties to the **Treaty**, in the exercise of their sovereign rights over their **Plant Genetic Resources for Food and Agriculture**, have established a **Multilateral System** both to facilitate access to **Plant Genetic Resources for Food and Agriculture** and to share, in a fair and equitable way, the benefits arising from the utilization of these resources, on a complementary and mutually reinforcing basis;

Articles 4, 11, 12.4 and 12.5 of the **Treaty** are borne in mind;

The diversity of the legal systems of the Contracting Parties with respect to their national procedural rules governing access to courts and to arbitration, and the obligations arising from international and

regional conventions applicable to these procedural rules, are recognized;

Article 12.4 of the **Treaty** provides that facilitated access under the **Multilateral System** shall be provided pursuant to a Standard Material Transfer Agreement, and the **Governing Body** of the **Treaty**, in its Resolution 1/2006 of 16 June 2006, adopted the Standard Material Transfer Agreement.

---

[1] *Note by the Secretariat*: as suggested by the Legal Working Group during the Contact Group for the Drafting of the Standard Material Transfer Agreement, defined terms have, for clarity, been put in bold throughout.

* In the event that the SMTA is used for the transfer of Plant Genetic Resources for Food and Agriculture other than those listed in *Annex I* of the Treaty:
The references in the SMTA to the "Multilateral System" shall not be interpreted as limiting the application of the SMTA to *Annex I* Plant Genetic Resources for Food and Agriculture, and in the case of Article 6.2 of the SMTA shall mean "under this Agreement";
The reference in Article 6.11 and Annex 3 of the SMTA to "Plant Genetic Resources for Food and Agriculture belonging to the same crop, as set out in *Annex I* to the Treaty" shall be taken to mean "Plant Genetic Resources for Food and Agriculture belonging to the same crop".

ARTICLE 1 — PARTIES TO THE AGREEMENT

1.1 The present Material Transfer Agreement (hereinafter referred to as "**this Agreement**") is the Standard Material Transfer Agreement referred to in Article 12.4 of the **Treaty**.

1.2 **This Agreement** is:

BETWEEN:

………
………………………………………………………………… (*Name and address of the provider or providing institution, name of authorized official, contact information for authorized official*) (hereinafter referred to as "the **Provider**"),

AND:

………...
……………………………………………………………………… (*Name and address of the recipient or recipient institution, name of authorized official, contact information for authorized official*[*]) (hereinafter referred to as "the **Recipient**").

1.3 The parties to **this Agreement** hereby agree as follows:

ARTICLE 2 — DEFINITIONS

In **this Agreement** the expressions set out below shall have the following meaning:

"***Available without restriction***": a **Product** is considered to be available without restriction to others for further research and breeding when it is available for research and breeding without any legal or contractual obligations, or technological restrictions, that would preclude using it in the manner specified in the **Treaty**.

"***Genetic material***" means any material of plant origin, including reproductive and vegetative propagating material, containing functional units of heredity.

"***Governing Body***" means the **Governing Body** of the **Treaty**.

"***Multilateral System***" means the **Multilateral System** established under Article 10.2 of the **Treaty**.

"***Plant Genetic Resources for Food and Agriculture***" means any **genetic material** of plant origin of actual or potential value for food and agriculture.

"Plant Genetic Resources for Food and Agriculture under Development" means material derived from the **Material**, and hence distinct from it, that is not yet ready for **commercialization** and which the developer intends to further develop or to transfer to another person or entity for further development. The period of development for the **Plant Genetic Resources for Food and Agriculture under Development** shall be deemed to have ceased when those resources are **commercialized** as a **Product**.

"***Product***" means **Plant Genetic Resources for Food and Agriculture** that incorporate[2] the **Material** or any of its genetic parts or components that are ready for **commercialization**, excluding commodities and other products used for food, feed and processing.

"***Sales***" means the gross income resulting from the **commercialization** of a **Product** or **Products**, by the **Recipient**, its affiliates, contractors, licensees and lessees.

---

[*] *Insert as necessary. Not applicable for shrink-wrap and click-wrap Standard Material Transfer Agreements.*

A "shrink-wrap" Standard Material Transfer Agreement is where a copy of the Standard Material Transfer Agreement is included in the packaging of the **Material**, and the **Recipient's** acceptance of the **Material** constitutes acceptance of the terms and conditions of the Standard Material Transfer Agreement.

A "click-wrap" Standard Material Transfer Agreement is where the agreement is concluded on the internet and the **Recipient** accepts the terms and conditions of the Standard Material Transfer Agreement by clicking on the appropriate icon on the website or in the electronic version of the Standard Material Transfer Agreement, as appropriate.

[2] As evidenced, for example, by pedigree or notation of gene insertion.

"***Sales***" means the gross income resulting from the **commercialization** of a **Product** or **Products**, by the **Recipient**, its affiliates, contractors, licensees and lessees.

"***To commercialize***" means to sell a **Product** or **Products** for monetary consideration on the open market, and "**commercialization**" has a corresponding meaning. **Commercialization** shall not include any form of transfer of **Plant Genetic Resources for Food and Agriculture under Development**.

ARTICLE 3 — SUBJECT MATTER OF THE MATERIAL TRANSFER AGREEMENT

The **Plant Genetic Resources for Food and Agriculture** specified in *Annex 1* to **this Agreement** (hereinafter referred to as the "**Material**") and the available related information referred to in Article 5b and in *Annex 1* are hereby transferred from the **Provider** to the **Recipient** subject to the terms and conditions set out in **this Agreement**.

ARTICLE 4 — GENERAL PROVISIONS

4.1 **This Agreement** is entered into within the framework of the **Multilateral System** and shall be implemented and interpreted in accordance with the objectives and provisions of the **Treaty**.

4.2 The parties recognize that they are subject to the applicable legal measures and procedures, that have been adopted by the Contracting Parties to the **Treaty**, in conformity with the **Treaty**, in particular those taken in conformity with Articles 4, 12.2 and 12.5 of the **Treaty**.[3]

4.3 The parties to **this Agreement** agree that (*the entity designated by the **Governing Body***),[4] acting on behalf of the **Governing Body** of the **Treaty** and its **Multilateral System**, is the third party beneficiary under **this Agreement**.

4.4 The third party beneficiary has the right to request the appropriate information as required in Articles 5e, 6.5c, 8.3 and *Annex, 2 paragraph 3,* to **this Agreement**.

4.5 The rights granted to the (*the entity designated by the **Governing Body***) above do not prevent the **Provider** and the **Recipient** from exercising their rights under **this Agreement**.

ARTICLE 5 — RIGHTS AND OBLIGATIONS OF THE PROVIDER

The **Provider** undertakes that the **Material** is transferred in accordance with the following provisions of the **Treaty**:

a) Access shall be accorded expeditiously, without the need to track individual accessions and free of charge, or, when a fee is charged, it shall not exceed the minimal cost involved;

[3] In the case of the International Agricultural Research Centres of the Consultative Group on International Agricultural Research (CGIAR) and other international institutions, the Agreement between the Governing Body and the CGIAR Centres and other relevant institutions will be applicable.

[4] *Note by the Secretariat:* by Resolution 2/2006, the Governing Body "invite[d] the Food and Agriculture Organization of the United Nations, as the Third Party Beneficiary, to carry out the roles and responsibilities as identified and prescribed in the Standard Material Transfer Agreement, under the direction of the Governing Body, in accordance with the procedures to be established by the Governing Body at its next session". Upon acceptance by the FAO of this invitation, the term, "the entity designated by the Governing Body", will be replaced throughout the document by the term, "the Food and Agriculture Organization of the United Nations".

b) All available passport data and, subject to applicable law, any other associated available non-confidential descriptive information, shall be made available with the **Plant Genetic Resources for Food and Agriculture** provided;

c) Access to **Plant Genetic Resources for Food and Agriculture under Development**, including material being developed by farmers, shall be at the discretion of its developer, during the period of its development;

d) Access to **Plant Genetic Resources for Food and Agriculture** protected by intellectual and other property rights shall be consistent with relevant international agreements, and with relevant national laws;

e) The **Provider** shall periodically inform the **Governing Body** about the Material Transfer Agreements entered into, according to a schedule to be established by the **Governing Body**. This information shall be made available by the **Governing Body** to the third party beneficiary.[5]

ARTICLE 6 — RIGHTS AND OBLIGATIONS OF THE RECIPIENT

6.1 The **Recipient** undertakes that the **Material** shall be used or conserved only for the purposes of research, breeding and training for food and agriculture. Such purposes shall not include chemical, pharmaceutical and/or other non-food/feed industrial uses.

6.2 The **Recipient** shall not claim any intellectual property or other rights that limit the facilitated access to the **Material** provided under **this Agreement**, or its genetic parts or components, in the form received from the **Multilateral System**.

6.3 In the case that the **Recipient** conserves the **Material** supplied, the **Recipient** shall make the **Material**, and the related information referred to in Article 5b, available to the **Multilateral System** using the Standard Material Transfer Agreement.

6.4 In the case that the **Recipient** transfers the **Material** supplied under **this Agreement** to another person or entity (hereinafter referred to as "the **subsequent recipient**"), the **Recipient** shall

a) do so under the terms and conditions of the Standard Material Transfer Agreement, through a new material transfer agreement; and

b) notify the **Governing Body**, in accordance with Article 5e.

On compliance with the above, the **Recipient** shall have no further obligations regarding the actions of the **subsequent recipient**.

6.5 In the case that the **Recipient** transfers a **Plant Genetic Resource for Food and Agriculture under Development** to another person or entity, the **Recipient** shall:

[5] *Note by the Secretraiat:* The Standard Material Transfer Agreement makes provision for information to be provided to the **Governing Body**, in the following Articles: 5e, 6.4b, 6.5c and 6.11h, as well as in *Annex 2*, paragraph 3, *Annex 3*, paragraph 4, and in *Annex 4*. Such informationshould be submitted to:

The Secretary
International Treaty on Plant Genetic Resources for Food and Agriculture Food and
Agriculture Organization of the United Nations
I-00100 Rome, Italy

a) do so under the terms and conditions of the Standard Material Transfer Agreement, through a new material transfer agreement, provided that Article 5a of the Standard Material Transfer Agreement shall not apply;

b) identify, in *Annex 1* to the new material transfer agreement, the **Material** received from the **Multilateral System**, and specify that the **Plant Genetic Resources for Food and Agriculture under Development** being transferred are derived from the **Material**;

c) notify the **Governing Body**, in accordance with Article 5e; and

d) have no further obligations regarding the actions of any **subsequent recipient**.

6.6 Entering into a material transfer agreement under paragraph 6.5 shall be without prejudice to the right of the parties to attach additional conditions, relating to further product development, including, as appropriate, the payment of monetary consideration.

6.7 In the case that the **Recipient commercializes** a **Product** that is a **Plant Genetic Resource for Food and Agriculture** and that incorporates **Material** as referred to in Article 3 of **this Agreement**, and where such **Product** is not **available without restriction** to others for further research and breeding, the **Recipient** shall pay a fixed percentage of the **Sales** of the **commercialized Product** into the mechanism established by the **Governing Body** for this purpose, in accordance with *Annex 2* to **this Agreement**.

6.8 In the case that the **Recipient commercializes** a **Product** that is a **Plant Genetic Resource for Food and Agriculture** and that incorporates **Material** as referred to in Article 3 of **this Agreement** and where that **Product** is **available without restriction** to others for further research and breeding, the **Recipient** is encouraged to make voluntary payments into the mechanism established by the **Governing Body** for this purpose in accordance with *Annex 2* to **this Agreement**.

6.9 The **Recipient** shall make available to the **Multilateral System**, through the information system provided for in Article 17 of the **Treaty**, all non-confidential information that results from research and development carried out on the **Material**, and is encouraged to share through the **Multilateral System** non-monetary benefits expressly identified in Article 13.2 of the **Treaty** that result from such research and development. After the expiry or abandonment of the protection period of an intellectual property right on a **Product** that incorporates the **Material**, the **Recipient** is encouraged to place a sample of this **Product** into a collection that is part of the **Multilateral System**, for research and breeding.

6.10 A **Recipient** who obtains intellectual property rights on any **Products** developed from the **Material** or its components, obtained from the **Multilateral System**, and assigns such intellectual property rights to a third party, shall transfer the benefit-sharing obligations of **this Agreement** to that third party.

6.11 The **Recipient** may opt as per *Annex 4*, as an alternative to payments under Article 6.7, for the following system of payments:

a) The **Recipient** shall make payments at a discounted rate during the period of validity of the option;
b) The period of validity of the option shall be ten years renewable in accordance with *Annex 3* to **this Agreement**;
c) The payments shall be based on the **Sales** of any **Products** and of the sales of any other products that are **Plant Genetic Resources for Food and Agriculture** belonging to the same crop, as set out in Annex 1 to the **Treaty**, to which the **Material** referred to in *Annex 1* to **this Agreement** belongs;
d) The payments to be made are independent of whether or not the **Product** is **available without restriction**;
e) The rates of payment and other terms and conditions applicable to this option, including the discounted rates are set out in *Annex 3* to **this Agreement**;
f) The **Recipient** shall be relieved of any obligation to make payments under Article 6.7 of **this Agreement** or any previous or subsequent Standard Material Transfer Agreements entered into in respect of the same crop;
g) After the end of the period of validity of this option the **Recipient** shall make payments on any **Products** that incorporate **Material** received during the period in which this Article was in force, and where such **Products** are not **available without restriction**. These payments will be calculated at the same rate as in paragraph (a) above;
h) The **Recipient** shall notify the **Governing Body** that he has opted for this modality of payment. If no notification is provided the alternative modality of payment specified in Article 6.7 will apply.

ARTICLE 7 — APPLICABLE LAW

The applicable law shall be General Principles of Law, including the UNIDROIT Principles of International Commercial Contracts 2004, the objectives and the relevant provisions of the **Treaty**, and, when necessary for interpretation, the decisions of the **Governing Body**.

ARTICLE 8 — DISPUTE SETTLEMENT

8.1 Dispute settlement may be initiated by the **Provider** or the **Recipient** or the (*the entity designated by the **Governing Body***), acting on behalf of the **Governing Body** of the **Treaty** and its **Multilateral System**.

8.2 The parties to **this Agreement** agree that the (*the entity designated by the **Governing Body***), representing the **Governing Body** and the **Multilateral System**, has the right, as a third party beneficiary, to initiate dispute settlement procedures regarding rights and

obligations of the **Provider** and the **Recipient** under **this Agreement**.

8.3 The third party beneficiary has the right to request that the appropriate information, including samples as necessary, be made available by the **Provider** and the **Recipient**, regarding their obligations in the context of **this Agreement**. Any information or samples so requested shall be provided by the **Provider** and the **Recipient**, as the case may be.

8.4 Any dispute arising from **this Agreement** shall be resolved in the following manner:

a) Amicable dispute settlement: The parties shall attempt in good faith to resolve the dispute by negotiation.
b) Mediation: If the dispute is not resolved by negotiation, the parties may choose mediation through a neutral third party mediator, to be mutually agreed.
c) Arbitration: If the dispute has not been settled by negotiation or mediation, any party may submit the dispute for arbitration under the Arbitration Rules of an international body as agreed by the parties to the dispute. Failing such agreement, the dispute shall be finally settled under the Rules of Arbitration of the International Chamber of Commerce, by one or more arbitrators appointed in accordance with the said Rules. Either party to the dispute may, if it so chooses, appoint its arbitrator from such list of experts as the Governing Body may establish for this purpose; both parties, or the arbitrators appointed by them, may agree to appoint a sole arbitrator, or presiding arbitrator as the case may be, from such list of experts. The result of such arbitration shall be binding.

ARTICLE 9 — ADDITIONAL ITEMS

Warranty

9.1 The **Provider** makes no warranties as to the safety of or title to the **Material**, nor as to the accuracy or correctness of any passport or other data provided with the **Material**. Neither does it make any warranties as to the quality, viability, or purity (genetic or mechanical) of the **Material** being furnished. The phytosanitary condition of the **Material** is warranted only as described in any attached phytosanitary certificate. The **Recipient** assumes full responsibility for complying with the recipient nation's quarantine and biosafety regulations and rules as to import or release of **genetic material**.

Duration of Agreement

9.2 **This Agreement** shall remain in force so long as the **Treaty** remains in force.

ARTICLE 10 — SIGNATURE/ACCEPTANCE

The **Provider** and the **Recipient** may choose the method of acceptance unless either party requires
this Agreement to be signed.

Option 1 –Signature*

I, ..., represent and warrant that I have the authority to execute **this Agreement** on behalf of the **Provider** and acknowledge my institution's responsibility and obligation to abide by the provisions of **this Agreement**, both by letter and

in principle, in order to promote the conservation and sustainable use of **Plant Genetic Resources for Food and Agriculture**.

Signature.. Date...

Name of the **Provider:** ....

I, ..., represent and warrant that I have the authority to execute **this Agreement** on behalf of the **Recipient** and acknowledge my institution's responsibility and obligation to abide by the provisions of **this Agreement**, both by letter and in principle, in order to promote the conservation and sustainable use of **Plant Genetic Resources for Food and Agriculture**.

Signature.. Date...

Name of the **Recipient:** ....

<u>Option 2 – Shrink-wrap Standard Material Transfer Agreements*</u>

The **Material** is provided conditional on acceptance of the terms of **this Agreement**. The provision of the **Material** by the **Provider** and the **Recipient's** acceptance and use of the **Material** constitutes acceptance of the terms of **this Agreement**.

<u>Option 3 – Click-wrap Standard Material Transfer Agreement*</u>

☐ I hereby agree to the above conditions.

* Where the **Provider** chooses signature, only the wording in Option 1 will appear in the Standard Material Transfer Agreement. Similarly where the **Provider** chooses either shrink-wrap or click-wrap, only the wording in Option 2 or Option 3, as appropriate, will appear in the Standard Material Transfer Agreement. Where the "click-wrap" form is chosen, the **Material** should also be accompanied by a written copy of the Standard Material Transfer Agreement.

LIST OF MATERIALS PROVIDED

This *Annex* contains a list of the **Material** provided under **this Agreement**, including the associated information referred to in Article 5b.

This information is either provided below or can be obtained at the following website: (*URL*).

The following information is included for each **Material** listed: all available passport data and, subject to applicable law, any other associated, available, non-confidential descriptive information.

(*List*)

<u>RATE AND MODALITIES OF PAYMENT UNDER ARTICLE 6.7 OF THIS</u>

AGREEMENT

1. If a **Recipient**, its affiliates, contractors, licensees, and lessees, **commercializes** a **Product** or **Products**, then the **Recipient** shall pay one point-one percent (1.1 %) of the **Sales** of the **Product** or **Products** less thirty percent (30%); except that no payment shall be due on any **Product** or **Products** that:

(a) are **available without restriction** to others for further research and breeding in accordance with Article 2 of **this Agreement**;

(b) have been purchased or otherwise obtained from another person or entity who either has already made payment on the **Product** or **Products** or is exempt from the obligation to make payment pursuant to subparagraph (a) above;

(c) are sold or traded as a commodity.

2. Where a **Product** contains a **Plant Genetic Resource for Food and Agriculture** accessed from the **Multilateral System** under two or more material transfer agreements based on the Standard Material Transfer Agreement only one payment shall be required under paragraph 1 above.

3. The **Recipient** shall submit to the **Governing Body**, within sixty (60) days after each calendar year ending December 31st, an annual report setting forth:

(a) the **Sales** of the **Product** or **Products** by the **Recipient**, its affiliates, contractors, licensees and lessees, for the twelve (12) month period ending on December 31st;

(b) the amount of the payment due; and

(c) information that allows for the identification of any restrictions that have given rise to the benefit-sharing payment.

4. Payment shall be due and payable upon submission of each annual report. All payments due to the **Governing Body** shall be payable in (*specified currency*)[6] for the account of (*the Trust Account or other mechanism established by the **Governing Body** in accordance with Article 19.3f of the **Treaty***).[7]

[6] *Note by the Secretariat:* The Governing Body has not yet considered the question of currency of payment. Until it does so, Standard Material Transfer Agreements should specify United States dollars (US$).

[7] *Note by the Secretariat:* This is the Trust Account provided for in Article 6.3 of the Financial Rules, as approved by the Governing Body (*Appendix E* to this Report). The details of the Trust Account when established, will be intoduced here, and communicated to Contract Parties.

TERMS AND CONDITIONS OF THE ALTERNATIVE PAYMENTS SCHEME UNDER ARTICLE 6.11 OF THIS AGREEMENT

1. The discounted rate for payments made under Article 6.11 shall be zero point five percent (0.5
%) of the **Sales** of any **Products** and of the sales of any other products that are **Plant Genetic Resources for Food and Agriculture** belonging to the same crop, as set out in

Annex 1 to the **Treaty**, to which the **Material** referred to in *Annex 1* to **this Agreement** belong.

2. Payment shall be made in accordance with the banking instructions set out in paragraph 4 of
Annex 2 to this Agreement.

3. When the **Recipient** transfers **Plant Genetic Resources for Food and Agriculture under Development**, the transfer shall be made on the condition that the **subsequent recipient** shall pay into the mechanism established by the **Governing Body** under Article 19.3f of the **Treaty** zero point five percent (0.5 %) of the **Sales** of any **Product** derived from such **Plant Genetic Resources for Food and Agriculture under Development**, whether the **Product** is **available or not without restriction**.

4. At least six months before the expiry of a period of ten years counted from the date of signature of **this Agreement** and, thereafter, six months before the expiry of subsequent periods of five years, the **Recipient** may notify the **Governing Body** of his decision to opt out from the application of this Article as of the end of any of those periods. In the case the **Recipient** has entered into other Standard Material Transfer Agreements, the ten years period will commence on the date of signature of the first Standard Material Transfer Agreement where an option for this Article has been made.

5. Where the **Recipient** has entered or enters in the future into other Standard Material Transfer Agreements in relation to material belonging to the same crop[s], the **Recipient** shall only pay into the referred mechanism the percentage of sales as determined in accordance with this Article or the same Article of any other Standard Material Transfer Agreement. No cumulative payments will be required.

OPTION FOR CROP-BASED PAYMENTS UNDER THE ALTERNATIVE PAYMENTS SCHEME UNDER ARTICLE 6.11 OF THIS AGREEMENT

I …………………………………………………………………… declare to opt for payment in accordance with Article 6.11 of **this Agreement**.

Signature.. Date...[8]

[8] In accordance with Article 6.11h of the Standard Material Transfer Agreement, the option for this modality of payment will become operative only once notification has been provided by the **Recipient** to the **Governing Body**. The signed declaration opting for this modality of payment must be sent by the **Recipient** to the **Governing Body** at the following address, whichever method of acceptance of **this Agreement** (signature, shrink-wrap or click-wrap) has been chosen by the parties to **this Agreement**, and whether or not the **Recipient** has already indicated his acceptance of this option in accepting **this Agreement** itself:

The Secretary,
International Treaty on Plant Genetic Resources for Food and Agriculture Food and Agriculture Organization of the United Nations
I-00100 Rome, Italy

The signed declaration must be accompanied by the following:

- The date on which **this Agreement** was entered into;
- The name and address of the **Recipient** and of the **Provider**;
- A copy of Annex 1 to **this Agreement**.

Additional Terms and Conditions to the

Standard Material Transfer Agreement (SMTA) for

Transgenic or Biotechnology Engineered (BE) Materials

SMTA ID No. ______________ **SMTA Date: ____________**

Article 1. Legal basis

The Provider breeds and develops crop materials such as beans, cassava, forages and rice as part of its breeding and biotechnology programs. These materials, including identified or characterized genes or sequences, inserted genes or sequences, DNA or RNA sequences, and plant parts together with associated information constitute **Plant Genetic Resources for Food and Agriculture under Development** (hereinafter referred to as "**PUD materials**") as per articles 5 c) and 6.5 of the SMTA.

The Provider provides access to **PUD materials**, including those *modified or engineered* through the use of *biotechnological* tools and methods resulting in the insertion or not of foreign genes in the plant materials (hereinafter referred to as "**PUD BE materials**") under these **Additional Terms and Conditions** to the SMTA, in accordance with the discretionary right granted to the developer set forth in article 5 c) of the SMTA and article 12 (e) of the International Treaty on Plant Genetic Resources for Food and Agriculture.

Article 2. PUD BE materials

The **PUD BE materials,** governed and provided under the present **Additional Terms and Conditions** and the associated SMTA are listed in *Annex 1* of the SMTA.

Article 3. Definitions

In addition to the definitions contained in Article 2 of the SMTA, the expressions set out below shall have the following meaning:

"***Commercial Purposes***" means the sale, lease, license or transfer of the **PUD BE materials** to any third party. Commercial Purposes shall also include, without any limitation, any use of the **PUD BE materials** aimed at generating an income; producing or manufacturing products for sale; carrying out research or development activities that result in any sale, license, lease or transfer of the **PUD BE materials** to a third party; or to provide a service for third parties incorporating, derived from or using the **PUD BE materials**.

"***Confidential Information***" means all non-public data, knowledge, information and materials of whatever nature and in whatever form, shape or media (including, but not limited to, written, oral and digital form) designated as "Confidential", "Restricted", "Proprietary", "Proprietary and Confidential", "Secret", or with any other similar term, provided by the disclosing party to the receiving party, or declared as such by the parties during the term of this agreement.

"***Derived BE materials***" means any tangible or physical biological material and/or genetic materials and information other than the **PUD BE materials**, which contain or incorporate the **PUD BE materials**, or are derived from the **PUD BE materials**.

"***Intellectual Property Rights***" means any legal right that protects, among other things, inventions, discoveries, ideas, improvements, works of authorship, data, materials, methods, know-how and processes, and includes patents, designs, trademarks, plant variety protection certificates, semiconductor layouts and corresponding applications, as well as copyright, databases, know how, trade secrets, and any similar right including all applications (or rights to apply) for, and renewals or extensions of, such rights recognized from time to time in any jurisdiction of the world, together with all rights of action in relation to the infringement of the above.

"***PUD BE materials***" means **Plant Genetic Resources for Food and Agriculture under Development** that have been *genetically modified through biotechnology tools, transgenic or biotechnology engineered*, including using genome editing techniques, or through technologies that could be subject of regulation in different jurisdictions and contain a trait or traits, or exhibit characteristics or properties due to such processes.

"***Resulting Information***" means any data, information, know how, techniques, practices, methods, processes, testing procedures, software, and any other non-biological materials regardless of the form or media in which they are generated, developed, disclosed, whether or not patentable or susceptible of any other form of legal protection, that are created, discovered, generated or developed as a result of activities carried out with the **PUD BE materials**.

Article 4. Additional Terms and Conditions

The present Additional Terms and Conditions to the SMTA as well as the associated SMTA are used for the transfer of **PUD BE materials** as listed in *Annex 1* in the context and under the project "*Transformative strategy for controlling rice disease in developing countries Phase II*" (the "Project") (Global Development Grant # OPP1155704) lead by the Heinrich Heine Universität (HHU) (Düsseldorf) in collaboration with Centro Internacional de Agricultura Tropical (CIAT), the International Rice Research Institute (IRRI), University of Florida (UF), University of Missouri (UM), and the Institut de Recherche pour le Développement (IRD) (collectively referred to as "Collaborators"). The collaborator's work under the Project is regulated by the Subaward No. F-2016/1166 and the Agreement on a Global Access Strategy for the Project under the grant # OPP1155704.

The **Recipient** accepts to be bound and to respect the following **Additional Terms and Conditions**:

1. The **Recipient** is authorized to use the **PUD BE materials** under a free of charge, non-exclusive, non-transferable, non-commercial, non-sublicensable license for research, test and evaluation, and educational purposes under and for the purposes of the Project. For any other purposes the **Recipient** shall previously consult with the **Provider** and seek a separate agreement.
2. The **Recipient** shall ensure that the **PUD BE materials** are only used for the authorized purposes and under its own premises.
3. Transfer of **PUD BE materials** to any third party is not authorized. Any third party requesting a sample of **PUD BE materials** shall be referred to the **Provider**. For the avoidance of doubt, in case the **Recipient** wishes to transfer the **PUD BE materials** to any of the Collaborators under and for the purposes of the Project, the **Recipient** is entitled to do it by issuing a new Additional Terms and Conditions and associated SMTA and the subsequent recipient shall be bound by the same terms as the original Recipient. The **Recipient** shall notify the **Provider** about such subsequent transfer.
4. Use of the **PUD BE materials** for **Commercial Purposes** or for post-Project development is not authorized. For **Commercial Purposes** or for post-Project development, the **Recipient** must first contact the **Provider** and seek a separate agreement.
5. The **Recipient** shall not claim ownership, neither seek **Intellectual Property Rights** over the **PUD BE materials**, its genetic parts or components, and/or its associated provided information, in the form received from the **Provider**. Further, as applicable to the PUD BE materials, its parts and components and associated provided information, IP protection and the management of Funded Developments under the Project shall be carried out according to the terms agreed upon under the Global Access Strategy Agreement.
6. Any **Resulting Information** and **Derived BE materials** generated or developed by the **Recipient** alone as Funded Developments under the Project shall be property of the **Recipient**, except to the extent of the property on the **PUD BE materials** that lies with the **Provider**. In case the **Provider** is involved in the generation or development of **Resulting Information** and/or **Derived BE materials**, including any invention, ownership shall be joint between the parties and the parties shall proceed as per terms agreed upon under the Global Access Strategy Agreement.
7. The **Recipient** is requested to share all **Resulting Information** with the **Provider**, regardless of ownership, under a non-exclusive, worldwide, free of charge, irrevocable license, for research,

evaluation, development, educational and breeding purposes. Following institutional Open Access policies, the **Provider** shall make accessible and available **Resulting Information** through its institutional repositories. If there were **Confidential Information** of the **Provider** or of the **Recipient** linked to or contained in the **Resulting Information**, the **Provider** shall respect this and take it into account for information availability. Further, the parties agree that management of data and publication rights shall be governed by the terms agreed upon under the Global Access Strategy Agreement

8. Regardless of ownership, the **Recipient** shall grant the **Provider** a license to access and use any **Derived BE materials** under free of charge, non-exclusive, non-transferable, non-commercial, non-sublicensable terms for further research, test and evaluation purposes, and for any other purposes contemplated under and for the purposes of the Project. In case of dissemination, post-Project use or **Commercial Purposes**, the parties agree to enter into a separate agreement negotiated under fair and reasonable terms agreed upon by the parties.
9. In addition to the above terms, the **Recipient** shall:
    a. Use the **PUD BE materials** and **Derived BE materials** only in confined environments (e.g., laboratory, growth chamber, and green/glasshouse).
    b. Understand that the term "under its own premises" mentioned in numeral 2 rules out export of **PUD BE materials**.
    c. Use and dispose of the **PUD BE materials** and **Derived BE materials** in compliance with government issued regulations applicable in the geographical area of the **Recipient's** operations and according to institutional regulations/norms applicable to the handling and use of such materials, to transport of such materials and for protection of the environment.
    d. Not use **PUD BE materials** and **Derived BE materials** for any testing in humans or animals, including feeding to or consumption by humans or animals. The **Recipient** shall take all reasonable precautions to prevent **PUD BE materials** and **Derived BE materials** or components thereof from entering food, food products, feed or feed products.
    e. Notify and provide a copy of any proposed disclosure or publication of **Resulting Information** on the **PUD BE materials** and/or **Derived BE materials**, and apply publication rights according to the terms agreed upon under the Global Access Strategy Agreement.
    f. The **Recipient** shall acknowledge the **Provider** as the source of the **PUD BE materials** in any authorized disclosure or publication citing the said materials.
    g. In case of a peer-reviewed publication of **Resulting Information** based on the use of the **PUD BE materials**, the **Recipient** shall give to the **Provider** a copy of a final accepted authors' manuscript or the final printed publication, in print or in electronic form for its inclusion in the **Provider**'s institutional repository to make it available as soon as possible at the time of publication or according to the Open Access policy of the Funding organization of the Project.
    h. Provide an annual written report to the **Provider** on the activities carried out under this agreement or at any other frequency and reporting mode as agreed upon the Global Access Strategy Agreement.
10. The **Recipient** accepts that the **PUD BE materials** are experimental in nature and provided "as is". In no event shall the **Provider** be liable for any use by the **Recipient** of the **PUD BE materials** or any loss, claim, damage, or liability, of whatsoever kind of nature, which may arise in connection to this agreement or the use, handling or storage of the **PUD BE material**.
11. The **Recipient** will hold the **Provider** harmless and indemnify the **Provider** for any claim made against the **Provider** by a third party arising from or connected with (i) the **Recipient**'s use, handling, storage, or any other activity connected to the **PUD BE materials**; (ii) the **Derived BE materials**; (iii) any breach of this agreement by the **Recipient**; and (iv) any gross negligence or willful misconduct by the **Recipient**.
12. Neither of the parties shall disclose **Confidential Information** to any third party nor use such **Confidential Information** for any other purpose than the expressly authorized by the disclosing party. The receiving party shall use the same degree of care to protect **Confidential Information** as it uses to protect its own information of similar nature, and in any event not less than reasonable

care under the circumstances. Confidentiality shall no longer apply to **Confidential Information** if the recipient party can demonstrate that: (i) it had possession of the information prior to disclosure; or (ii) the information is available to the public at the time of disclosure, or becomes available after disclosure through no fault of the receiving party; or (iii) the receiving party receives the information from a third party having the right to such information and who does not impose confidentiality.

13. The **Recipient** shall not assign this agreement to any third party without the prior written consent of the **Provider**. Any purported assignment in violation of the foregoing shall be null and void.
14. All other conditions as outlined in the associated SMTA and agreed to by the **Recipient**, apply except Article 5(a), as provided for in Article 6.5(a) of the SMTA.
15. The present **Additional Terms and Conditions** and the associated SMTA, including all its annexes, contain the whole agreement between the parties in respect of the subject matter and constitute this agreement. Nevertheless, as the parties have entered into a Subaward agreement and associated Global Access Strategy Agreement as above mentioned, the parties agree that if a conflict of terms exists between these Additional Terms and Conditions and the previous Subaward and Global Access Strategy Agrements, the latter shall prevail only to the extent of such conflicting term and only to the extent applicable to the PUD BE materials transferred under the present Additional Terms and Conditions.
16. The present **Additional Terms and Conditions** shall be in force and be binding upon the **Recipient** for the period of validity of the associated SMTA, unless the **Provider** agrees otherwise in writing.

I, the undersigned, hereby represent that I am legally authorized to sign these Additional Terms and Conditions on behalf of the Recipient legal entity

Name of Recipient legal entity: ______________________________________

Name of Recipient’s authorized representative: _________________________

Position of authorized representative: _________________________________

Signature: ____

Date: ____
